# The Murine Oral Metatranscriptome Reveals Microbial and Host Signatures of Periodontal Disease

**DOI:** 10.1177/00220345221149675

**Published:** 2023-03-08

**Authors:** S. Joseph, M. Carda-Diéguez, J. Aduse-Opoku, A. Alsam, A. Mira, M.A. Curtis

**Affiliations:** 1 Centre for Host-Microbiome Interactions, Faculty of Dentistry, Oral & Craniofacial Sciences, King’s College London, London, UK; 2 Oral Microbiome Lab, Department of Health and Genomics, FISABIO foundation, Valencia, Spain; 3 CIBER of Epidemiology and Public Health, Madrid, Spain

**Keywords:** metatranscriptomics, periodontitis, oral microbiome, mouse models, dysbiosis, bacterial metabolism, *P. gingivalis*, oral inflammation, oral biofilm

## Abstract

Periodontal disease is accompanied by alterations to cellular profiles and biological activities of both the subgingival microbiome and host tissues. Although significant progress has been made in describing the molecular basis of the homeostatic balance of host–commensal microbe interactions in health compared to the destructive imbalance in disease, particularly with respect to immune and inflammatory systems, few studies have attempted a comprehensive analysis in diverse host models. Here, we describe the development and application of a metatranscriptomic approach to analysis of host–microbe gene transcription in a murine periodontal disease model, based on oral gavage infection using *Porphyromonas gingivalis* in C57BL6/J mice. We generated 24 metatranscriptomic libraries from individual mouse oral swabs, representing health and disease. On average, 76% ± 11.7% reads in each sample belonged to the murine host genome and the remainder to the microbes. We found 3,468 (2.4% of the total) murine host transcripts differentially expressed between health and disease, of which 76% were overexpressed in periodontitis. Predictably, there were prominent alterations to genes and pathways linked with the host immune compartment in disease—the CD40 signaling pathway being the top enriched biological process in this data set. However, in addition, we observed significant alterations to other biological processes in disease, particularly cellular/metabolic processes and biological regulation. The number of differentially expressed microbial genes particularly indicated shifts in carbon metabolism pathways in disease with potential consequences for metabolic end-product formation. Together, these metatranscriptome data reveal marked changes between the gene expression patterns in both the murine host and microbiota, which may represent signatures of health and disease, providing the basis for future functional studies of prokaryotic and eukaryotic cellular responses in periodontal disease. In addition, the noninvasive protocol developed in this study will enable further longitudinal and interventionist studies of host–microbe gene expression networks.

## Introduction

Periodontal disease is characterized by a deregulated immune and inflammatory response and shift in the microbial population structure of subgingival biofilms, eventually leading to irreversible tissue destruction and ultimately tooth loss (Hajishengallis et al. 2011; Hajishengallis 2015; Joseph and Curtis 2021). Several studies on the oral microbiome have shown that the disturbed homeostasis represents not just an altered microbial population but also a shift in the gene expression patterns of these microbes (Duran-Pinedo et al. 2014; Ram-Mohan and Meyer 2020), including a potentially pathogenic contribution by the commensal bacterial population during disease progression (Yost et al. 2015). Furthermore, recent single-cell transcriptomic studies have helped identify the differentiating roles of stromal and epithelial cells of the oral mucosa in immune responses of periodontal disease (Williams et al. 2021), as well as in severity of disease progression (Caetano et al. 2021), indicative of broad gene expression changes across multiple cell types in human disease. Hence, it appears that the transcriptional landscape of both the microbiota and the host undergoes significant change in the development of disease.

Our understanding of the role of the oral microbiome and etiology of periodontal disease has particularly improved with the use of laboratory mouse models—including wild-type specific pathogen-free (SPF), germ-free (GF) mice, as well as gene-knockout mice (Hajishengallis et al. 2011; Payne et al. 2019; Hashim et al. 2021). We recently published a murine-specific oral microbiome database encompassing multiple laboratory SPF mouse strains, as well as 2 species of wild mice (Joseph et al. 2021), and representative draft bacterial genomes for the candidate species of the mouse oral microbiome. The availability of this mouse oral microbiome database (MOMD) resource enables analysis of the transcriptional changes in the microbiota in health versus disease in a well-defined model system. Since the mouse oral microbiota is known to be less diverse with low levels of uncultured organisms (Joseph et al. 2021), the application of this targeted reference database makes it possible to map transcripts to the source bacterial genomes with increased accuracy in this simple system.

We present here a murine oral metatranscriptomic study comparing health and periodontal disease using an oral gavage periodontitis mouse model (Baker et al. 2000). The *Porphyromonas gingivalis* oral gavage model has been used extensively in periodontal disease studies, particularly as it generates a chronic form of alveolar bone loss and inflammation, like that observed in human disease (Payne et al. 2019; Barel et al. 2022).

Moreover, we have developed a sample collection protocol using oral swabs as the starting material, thus demonstrating a convenient, noninvasive, and reproducible method of obtaining samples for nucleic acid extraction, despite low yields. The procedure of swabbing the oral cavity of the mouse enables collection of material derived from not only the microbial biofilms colonizing dental and soft tissue surfaces but also whole saliva and potentially also low levels of gingival crevicular fluid (GCF). It is recognized that saliva has the potential to act as a diagnostic fluid for oral diseases (Zhang et al. 2016; Chojnowska et al. 2018), although there have been few salivary transcriptome studies in humans (Spielmann et al. 2012; Hidayat et al. 2018). This method therefore has the potential benefit of sampling a wide range of host cells, including those of epithelial, stromal, and immune origin in the mouth. The results reveal a strong impact of disease on the host gene expression in multiple biological categories and oral microbial metabolism. This data set should help identify novel gene targets of interest that should benefit researchers in both laboratory models and also as potential therapeutic targets for clinical applications.

## Materials and Methods

The materials and methods are described in the Appendix.

## Results

**Figure 1.**
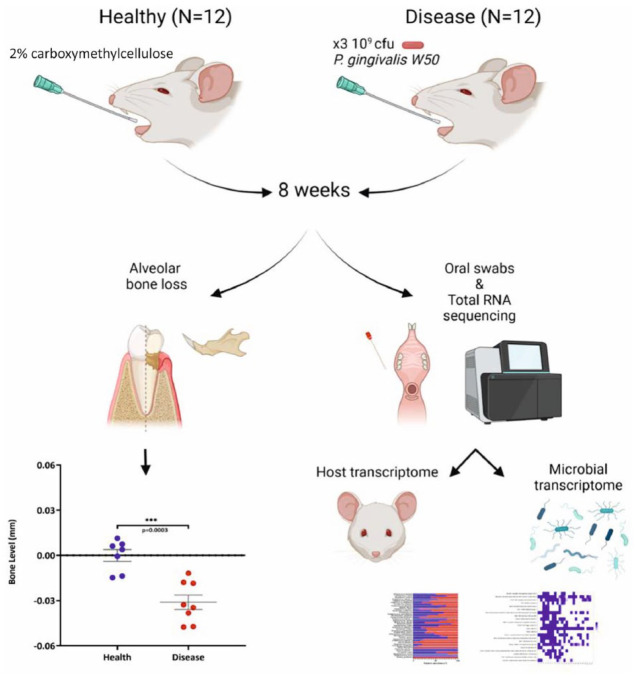
Flowchart describing the experimental plan adopted for this mouse oral metatranscriptomic study. Alveolar bone levels have been reported 8 wk after oral gavage in control (blue) and *Porphyromonas gingivalis*–treated mice (red). Bone loss was expressed as negative values relative to the baseline. Each point represents the mean ± SD bone level for an individual mouse, with horizontal lines representing the mean bone levels per group (***P < 0.0005). The graphic was generated using BioRender.

The flowchart in Figure 1 summarizes the experimental plan of this project. Compared to the healthy control group, the mice in the periodontal disease group that received 3 doses of *P. gingivalis* oral gavage showed significant loss of alveolar bone levels (Appendix Fig. 1) with a mean bone loss of 0.03 ± 0.01 mm over 8 wk (*P* = 0.0003) (Fig. 1).

### Periodontal Disease in Laboratory SPF Mice Reveals a Significant Difference in the Host Oral Metatranscriptomic Profile Compared to Healthy SPF Mice

In the Illumina Next-Seq transcriptome sequencing data from mouse oral swabs, 76% ± 11.7% of the reads per sample belonged to the murine host genome. The remaining messenger RNA (mRNA) reads selected for bacterial gene annotation represented 5% ± 1.6% of total microbial reads.

With filters of a minimum mean fold change (FC) threshold of 2 (FC >2), *P* < 0.05, and effect size >0.5, a total of 3,468 transcripts were found to be differentially represented between health and disease in the host gene transcripts. The top 100 genes in each condition, based on fold change, are presented in Appendix Table 1. The full list of 3,468 transcripts is available at https://doi.org/10.6084/m9.figshare.20364114.

**Figure 2.**
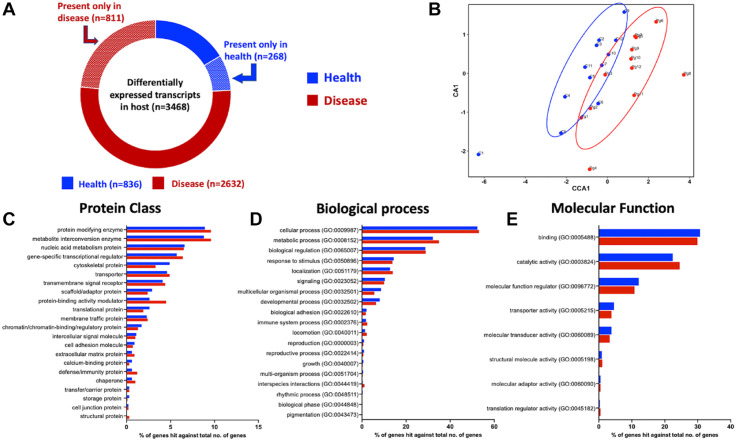
Snapshot of the differentially expressed genes between health (blue) and periodontal disease (red) in the mouse host oral metatranscriptome. (**A**) Breakdown of differentially expressed genes based on the presence and/or absence in health and disease with fold change >2. (**B**) Canonical coordinate analysis (CCA) plots showing the distribution of the 24 mouse samples based on host gene expression. (**C**) Percentage breakdown of the differentially expressed genes in health and disease based on protein class. (**D**) Percentage breakdown of the differentially expressed genes in health and disease based on biological process gene ontologies. (**E**) Percentage breakdown of the differentially expressed genes in health and disease based on molecular function gene ontologies.

Of these, 836 (24%) transcripts were overrepresented in the control mice, of which 268 (32%) were absent in all the diseased mice samples. The remaining 2,632 transcripts (76%) exhibited overexpression in the diseased group, of which 811 transcripts (30%) were absent in all the control mice samples (Fig. 2A). Canonical correlation analyses (CCAs) of the host transcripts revealed separation of the control and disease samples, with most of the variance being explained along coordinate axis 1 (Fig. 2B), albeit not statistically significant using the Adonis test (*P* = 0.76).

At the gene level, these differentially expressed transcripts represent 3,067 unique genes with similar distribution between health (24.9%) and disease (75.1%). Using the Panther Classification system, these genes were classified into 22 different classes of proteins (Fig. 2C).

In addition to the differential expression between health and disease, the healthy mouse oral metatranscriptome in this study generated 108,807 transcripts, the top 500 of which, based on gene expression levels, have been listed in Appendix Table 2 for the reader. The extensive list of transcripts, which can serve as a useful reference data set, is available at https://doi.org/10.6084/m9.figshare.20364117.

### The Host Oral Metatranscriptomic Profile in Periodontal Disease Is Characterized by Major Differences in Immune-Related Functions and Genes

**Figure 3.**
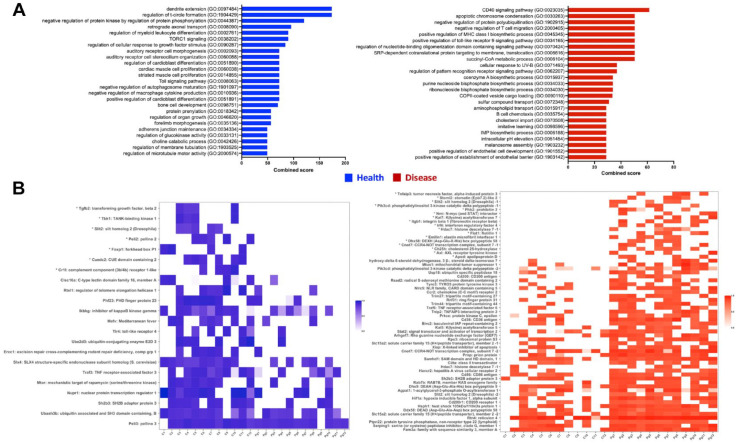
Gene enrichment analysis of the differentially expressed genes in the mouse oral host metatranscriptome using Enrichr. (**A**) Top 25 biological process gene ontologies ranked according to Enrichr calculated combined score in health (blue) and disease (red). (**B**) Heatmaps depicting relative gene expression levels for the genes linked with immune-associated biological processes that were enriched in health (blue) and disease (red) according to Enrichr analysis. Samples C1 to C12 on the x-axis are related to health, and samples Pg1 to Pg12 are related to disease. The genes have been listed in decreasing order of fold change, and the genes marked with asterisks refer to ones that were present only in the indicated condition and completely absent otherwise.

The Panther Classification system was used to categorize the differentially expressed gene list into gene ontologies based on both biological processes and molecular functions. The mouse host genes were classified into 20 different biological processes, the most abundant of which were cellular process, metabolic process, biological regulation, localization, and response to stimulus (Fig. 2D). Among molecular functions, the top categories were binding, catalysis, regulation of molecular functions, and transport (Fig. 2E). Gene enrichment analysis using Enrichr further characterized these gene ontology categories into subgroups. Based on this, among the biological processes, 114 gene ontology groups were enriched in the periodontal disease condition and 150 groups enriched in health (Appendix Table 3). The top 25 ontologies in each condition ranked according to combined score are presented in Figure 3A. In comparison, among molecular functions, using the same filtering criteria, the final list comprised only 34 gene ontology groups enriched in health and 26 in disease (Appendix Fig. 2).

The top candidate in the list of enriched biological processes in the disease category (Fig. 3A) was the CD40 signaling pathway driven by 5 genes overexpressed in disease (*CD86*, *ITGB1*, *RNF31*, *TNIP2*, and *PHB2*). We then investigated other enriched immune-related pathways in disease to obtain a list of 18 functional gene ontologies associated with immune signaling and regulation, driven by 56 genes in the mouse host genome that were differentially represented in disease (Fig. 3B; Appendix Table 4). In contrast, 12 enriched immune-associated pathways linked with only 22 genes were overrepresented in health (Fig. 3B; Appendix Table 4).

We also generated a list of immune-related genes from the differentially expressed gene set using keywords such as chemokine, interleukin, B-cell, T-cell, neutrophil, TLRs, and fibroblasts to generate a list of 64 genes—44 of which (69%) were overrepresented in disease (Appendix Fig. 3).

### The Oral Bacterial Metatranscriptomic Profile in SPF Mice Corresponds with the Simple Population Structure Previously Observed in the Oral Microbiome of SPF Mice

**Figure 4.**
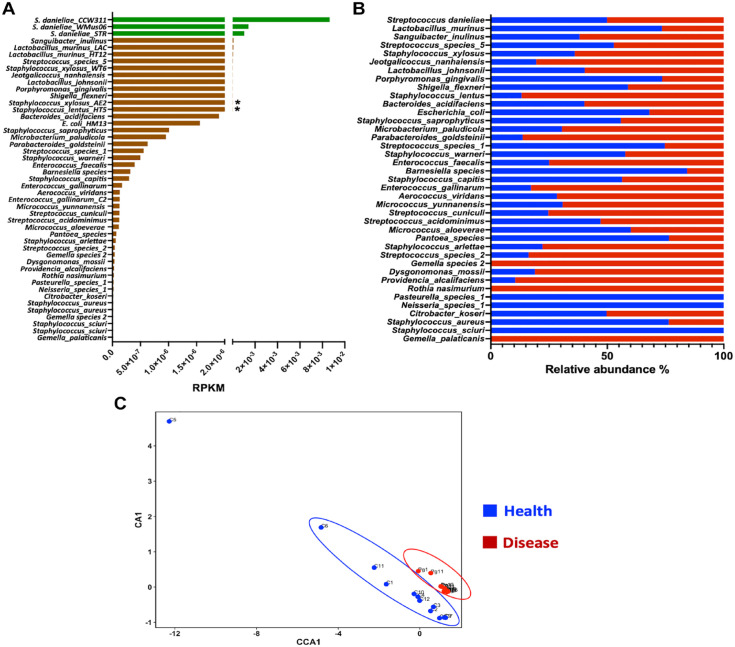
Taxonomic profile of the mouse oral bacterial metatranscriptome. (**A**) Mouse oral bacterial taxa plotted based on decreasing levels of abundance in the entire oral metatranscriptome. The most dominant taxon, *Streptococcus danieliae*, is indicated in green, and the bars with asterisks refer to the taxa that showed significant differences in gene expression between the health and disease conditions. RPKM, reads per Kbp of transcript per million (Mbp) of mapped reads. (**B**) Individual mouse oral bacterial taxa expressed as relative abundances in health (blue) and disease (red) conditions. (**C**) Canonical coordinate analysis (CCA) plots showing the distribution of the mouse samples based on bacterial species-level taxonomic profiles.

Of the total bacterial transcripts in this oral metatranscriptomic data set, 61% ± 17 % were annotated using the reference genomes from the Mouse Oral Microbiome Database (Joseph et al. 2021). Based on total mean abundance levels, the largest number of transcripts found matches against the 3 *Streptococcus danieliae* genomes, followed by *Sanguibacter inulinus*, *Lactobacillus murinus*, and *Staphylococcus xylosus* (Fig. 4A). Comparing mean species abundances between health and disease, a statistically significant difference was observed only for the genomes of 2 *Staphylococcus* species (*Staphylococcus xylosus AE2* and *Staphylococcus lentus HT5*) (Fig. 4A). Some of the least abundant species, such as *Gemella* species 2, *Rothia nasimurium*, *Pasteurella* species 1, *Neisseria* species 1, *Staphylococcus sciuri*, and *Gemella palaticanis*, were the only members that showed complete absence or presence in either condition (Fig. 4B). A plot of the CCA analyses at the species level showed separation of health and disease samples, with a tighter clustering of the diseased samples and also a strong outlier (sample C5) among the dispersed control samples (Fig. 4C).

### The Oral Bacterial Metatranscriptomic Profile in Periodontally Diseased SPF Mice Reveals Functional Differences in Metal Binding and Metabolic Processes

**Figure 5.**
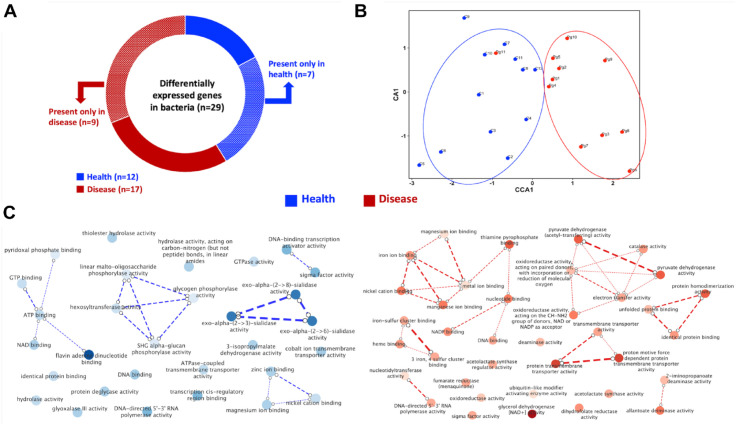
Differential gene expression profile in the mouse oral bacterial metatranscriptome. (**A**) Breakdown of the 29 differentially expressed genes in health (blue) and disease (red) with fold change >2. (**B**) Canonical coordinate analysis (CCA) plots showing the distribution of the mouse samples based on bacterial gene expression. (**C**) Revigo bacterial gene ontology (GO) networks of the molecular functions in health (blue) and disease (red) linked to the differentially expressed bacterial genes. The light to dark shades of the bubble colors correspond to the effect size of the fold change in gene expression. Highly similar GO terms are linked by dashed edges, where the line width indicates the degree of similarity.

Among the oral bacterial transcripts in this data set, using the same filtering criteria as the host gene set, 29 transcripts were differentially expressed between health and disease (Appendix Table 5). Of these, 12 were overrepresented in the control samples and 17 in disease (Fig. 5A).

CCA analyses of the bacterial transcripts also revealed separation of the health and disease samples; 67.2% of the variance explained along coordinate axis 1 (Appendix Fig. 4A), although the difference was not statistically significant. The graph was replotted after excluding the extreme outlier sample (Pg12), and the separation between health and disease groups was more clearly observed (Fig. 5B). These 23 samples were used for further analyses of the bacterial data set. The differentially expressed genes were characterized based on gene ontology network analysis for molecular functions in the bacterial population between health and disease (Fig. 5C). In health, prominent overrepresented networks related to sialidase activities and oligosaccharide/polysaccharide metabolism, while the notable molecular functions in the disease group were networks of functions related to the binding of metal ions (including heme binding, iron–sulfur binding) and pyruvate dehydrogenase–linked activities.

## Discussion

We demonstrate here a noninvasive protocol to obtain a global snapshot of the oral gene expression profile in murine models comprising both the host and the resident microbiota. Oral swabs are known to present challenges with nucleic acid yields (Adamowicz et al. 2014), more so in murine samples with low biomass (unpublished data). We have optimized a protocol to maximize individual RNA yields by repeated sampling of the same mouse, thus incorporating interindividual variations into our analyses by avoiding the pooling of samples. This, along with the inclusion of multiple experimental batches, has helped demonstrate the reproducibility and robustness of this protocol (Appendix Fig. 5).

In addition to the health and disease comparison, the gene expression atlas of the mouse oral transcripts generated from healthy SPF mice can serve as a reference database for future studies using murine models. The most abundant transcripts were largely of epithelial origin, including, for example, oral keratins (Appendix Table 2), which is to be expected given the sampling methodology, although differential analysis also demonstrated significant contributions from alternative host cell sources.

A predictable observation from this study has been the significant impact of periodontal disease on expression of host immune genes: gene enrichment data analysis revealed 30 enriched gene ontologies associated with 78 differentially expressed genes with immune function between health and disease (Fig. 3B). The top candidate among enriched immune gene ontologies, in disease, was the CD40 signaling pathway—an immune pathway previously associated with human periodontal disease (Chaturvedi et al. 2015; Su et al. 2020), as well as other related systemic conditions such as rheumatoid arthritis, inflammatory bowel disease (IBD), and atherosclerosis (Schonbeck et al. 2000; Criswell 2010; Senhaji et al. 2015). *CD86*, one of the costimulatory molecules reported to be upregulated in this pathway (Orima et al. 1999), was also overrepresented in the disease samples. In addition, there were 55 other genes associated with immune signaling and regulation involved with the 18 functional gene ontologies that were enriched in the disease condition, including the *CD200* and *CD200R1* gene set involved with leukocyte and macrophage migration. The CD200 molecule and its receptor have been linked with immunomodulation in response to pathogenic stimuli (Ngwa and Liu 2019) and also with periodontal disease and bone density levels (Björnfot Holmström et al. 2017). Also overrepresented in disease was the chemokine receptor gene *CCR2*, which, along with its corresponding ligand CCL2, is known to be involved in monocyte recruitment and tissue destruction in various oral diseases, including periodontitis (Silva et al. 2007; Barros et al. 2011). Other genes of relevance in this list have been discussed in the Appendix.

The immune-related pathways apart, the major biological processes in this data set were linked with cellular/metabolic processes and biological regulation, including enrichment of certain gene groups related to processes such as coenzyme A biosynthesis, protein translocation, and actin filament regulation. This enrichment of host metabolic and biochemical pathways in disease along with differentially expressed microbial genes associated with carbon metabolism pathways demonstrates the potential impact of metabolism in the mechanism of disease, an aspect that warrants further investigation.

In recent times, 2 separate studies (Caetano et al. 2021; Williams et al. 2021) generated reference human oral gene data sets via single-cell transcriptomics in health and periodontal disease, which revealed enhanced immune and inflammatory gene expression across multiple cell types, including epithelial and stromal cells, in disease. Despite differences in the specificity of sample collection, in the current study, we observed overlaps in gene groups linked with lymphocyte recruitment and adhesion, stromal and epithelial gingival proteins, and ligand–receptor interactions.

In a mixed microbial population, a successful metagenomic/metatranscriptomic study relies on the use of a targeted reference database for accurate taxonomic analysis. We have used our recently published murine-specific oral microbiome database as a reference for analyzing these data sets. In terms of microbial profile, we observe a composition corresponding to the population structure we have reported for the same mouse colony by culture as well as amplicon sequencing (Joseph et al. 2021), wherein the same 4 to 5 major taxa comprise 98% of the population. Of particular interest is the bacterium *S. danieliae*—a murine-specific streptococcus reported in a variety of SPF laboratory mouse backgrounds (Payne et al. 2019; Abusleme et al. 2020; Joseph et al. 2021). In this study, the organism was also the most predominant, thus showing that the most abundant organism was also the most functionally active member of the mouse oral microbiome.

Among the 29 differentially expressed bacterial genes, patterns of functional relevance were observed wherein number of the genes overrepresented in disease involved metabolic enzymes and transport activities—also observed in the gene ontology network analysis (Fig. 5B). KEGG (Kyoto Encyclopedia of Genes and Genomes) pathway analysis identified a cluster of genes overrepresented in disease, functionally linked with each other via the pyruvate metabolism and valine, leucine, and isoleucine biosynthesis pathways. Pyruvate metabolism was also one of the pathways differentially expressed in human oral microbial metatranscriptomic analysis (Ram-Mohan and Meyer 2020), with one of the genes involved being the same as that identified in our data set (k01653—*ilvH*, *ilvN*; acetolactate synthase I/III small subunit; Appendix Table 5).

The overexpression of bacterial metabolism genes could be an indicator of the pathogenic role of bacterial products such as short-chain fatty acids (SCFAs) in both homeostasis and driving periodontal disease. Consistent with this hypothesis, a saliva metabonomic analysis comparing health and periodontitis, reported by Aimetti et al. (2012), described bacterial metabolites, including differential levels of pyruvate, valine, succinate, and other SCFAs, as potential disease biomarkers.

Among molecular functions of the gene ontology network analysis in health, the overrepresentation of sialidase activities and oligosaccharide/polysaccharide metabolism potentially reflects the breakdown/utilization of salivary glycoproteins as the primary source of oral bacterial nutrition in health. In disease, a large network related to metal binding functions is observed—a trait observed among Gram-positive and Gram-negative pathogens as part of the infection process of disease (Mashruwala et al. 2015; Palmer and Skaar 2016). Notably, the periodontal pathogen *P. gingivalis* has a well-characterized heme acquisition mechanism via hemagglutinins and gingipains, key virulence factors in the pathogenicity of the organism (Curtis et al. 2002; Smalley et al. 2004). Among the biological processes observed in the gene ontology network analysis (Appendix Fig. 6), the main candidates in health were housekeeping activities related to repair and maintenance, while biggest networks in disease were linked with metabolic and enzymatic processes. This may reflect an increased rate of metabolism and rapid growth of microbes triggered in periodontal disease, potentially reflecting the emergence of GCF as a major source of microbial nutrition in disease as opposed to saliva-based nutrition in health.

Despite differences in the individual oral microbiomes of humans and mice, there are a number of similarities in the differential expression of the bacterial metatranscriptome in this study to those described in investigations in human periodontal disease. We observed a significant change in the metabolic properties in disease of the otherwise commensal microbial population. Similarly, oral metatranscriptomic studies in humans showed increased transcriptional activity by commensal organisms such as streptococci and *Veillonella* in expressing putative virulence factors in periodontal disease (Yost et al. 2015). Furthermore, a metatranscriptomic study of the subgingival microbiome in human health versus periodontitis reported that iron acquisition functions were upregulated in the disease (Duran-Pinedo et al. 2014), similar to the results in the current work.

In summary, we present a global snapshot of the impact of periodontal disease on the mouse oral host transcriptome and microbiome. Although conducted using an animal model, our study complements the cell atlas work conducted with human samples (Williams et al. 2021). We observe very significant shifts in host gene expression profiles when comparing health and disease, as well as a strong impact of disease development on immune and inflammatory gene function profiles and other biological processes, and importantly, we are able to reveal these changes in an oral swab metatranscriptome analysis, opening up the potential for rapid diagnostic strategies. In addition to the differentially expressed genes in this data set, whose products have previously been described to be of significance in studies of human periodontal disease, this investigation has revealed several candidate genes of interest not yet examined in humans, particularly those involved in cellular metabolism and biological regulation. We suggest that these may offer opportunities to investigate novel pathways/networks relevant to human disease.

We acknowledge that a limitation of our study is the inability of bulk RNA sequencing to identify local changes at the specific host cell/tissue level, particularly when obtained from an oral swab. Despite that, we provide the basis for future murine periodontitis model studies—through 1) the optimization of a low-yield RNA extraction and sequencing protocol, 2) the first use of a targeted murine microbiome reference database, and 3) generation of a murine oral host gene expression resource for the first time. Thus, this data set and methodology should offer potential for further in vitro and in vivo studies to investigate the pathogenesis of periodontal disease as well as mechanisms of host immunity in health and disease.

## Author Contributions

S. Joseph, M. Carda-Diéguez, contributed to conception and design, data acquisition, analysis and interpretation drafted and critically revised the manuscript; J. Aduse-Opoku, contributed to conception and data design, drafted and critically revised the manuscript; A. Alsam, contributed to data acquisition and data analysis, critically revised the manuscript; A. Mira, contributed to conception and design, data interpretation, critically revised the manuscript; M.A. Curtis, contributed to conception and design, data interpretation, drafted and critically revised the manuscript. All authors gave final approval and agreed to be accountable for all aspects of the work.

## Supplemental Material

sj-docx-1-jdr-10.1177_00220345221149675 – Supplemental material for The Murine Oral Metatranscriptome Reveals Microbial and Host Signatures of Periodontal Disease Click here for additional data file. Supplemental material, sj-docx-1-jdr-10.1177_00220345221149675 for The Murine Oral Metatranscriptome Reveals Microbial and Host Signatures of Periodontal Disease by S. Joseph, M. Carda-Diéguez, J. Aduse-Opoku, A. Alsam, A. Mira and M.A. Curtis in Journal of Dental Research
