# Supplementary material for: The Murine Oral Metatranscriptome Reveals Microbial and Host Signatures of Periodontal Disease
Source: J Dent Res. 2023 Mar 8;102(5):565–73. doi: 10.1177/00220345221149675 (PMC10152569; doi:10.1177/00220345221149675)
Supplement: sj-docx-1-jdr-10.1177_00220345221149675 – Supplemental material for The Murine Oral Metatranscriptome Reveals Microbial and Host Signatures of Periodontal Disease [file sj-docx-1-jdr-10.1177_00220345221149675.docx]

**The murine oral metatranscriptome reveals microbial and host signatures of periodontal disease.**

Susan Joseph^1*^, Miguel Carda-Diéguez^2^, Joseph Aduse-Opoku^1^, Asil Alsam^1^, Alex Mira^2,3^, Michael A Curtis^1*^

^1^ Centre for Host-Microbiome Interactions, Faculty of Dentistry, Oral & Craniofacial Sciences, King’s College London, London SE1 9RT, UK

^2^ Oral Microbiome Lab, Department of Health and Genomics, FISABIO foundation, 46020 Valencia, Spain

^3^ CIBER of Epidemiology and Public Health, Madrid, Spain

**APPENDIX MATERIALS AND METHODS**

***Mouse model of periodontal disease***

All animal experiments were conducted in accredited facilities in accordance with the UK Animals (Scientific Procedures) Act 1986 (Home Office license number P5DB2B893) and complies with the ARRIVE 2.0 guidelines. Conventional specific pathogen-free (SPF) C57/BL6J mice, derived from an original germ-free (GF) mouse colony, were maintained in individually ventilated cages (IVC) as an in-house colony at the animal care facilities of King’s College London.

The entire dataset for this study included 24 mice - 12 in the sham treated control group (Health) and 12 in the *Porphyromonas gingivalis* treated group (Disease). Each individual mouse was treated independently and was considered an experimental unit for this study. Power calculations for our proposed experiments were performed using the statistical program G*power (Faul et al. 2007). When based on an unpaired two-tailed t-test, in order to obtain an expected significance level of 0.05 and effect size of 2, it was shown that a sample size of 6 should be sufficient for these studies, as has also been used in most other published studies of this nature. In order to account for any unexpected experimental losses, we have used 12 mice in each group for this study. Only one of the authors (S.J) was aware of the group allocation at the different stages of the animal experiment.

The mice were in the age range of 9-13 weeks at the beginning of the experiment; a mix of males and females, which were randomly assigned to each group using the randomising function in MS Excel and were maintained in IVC cages for the entire duration of the study.

*P. gingivalis* strain W50 was grown on blood agar plates containing 5% defibrinated horse blood or in Brain Heart Infusion broth (BHI) (Oxoid) supplemented with haemin (5 μg/mL) in an anaerobic atmosphere of 80% N_2_, 10% H_2_ and 10% CO_2_ at 37^o^C (Don Whitley Scientific).

Oral gavage with *Porphyromonas gingivalis* was performed as described previously (Baker et al. 2000; Payne et al. 2019). Mice were orally inoculated by means of a ball-ended feeding needle three times at two-day intervals with 10^9^ colony forming units (CFU) of *P. gingivalis* W50 suspended in 100 µl of 2% carboxy-methylcellulose (Sigma) vehicle. Sham controls received vehicle alone. The control mice were handled first every time to avoid the possibility of any carry-over bacterial contamination. Adequate care was taken during the procedure to ensure correct dosing and the animals were monitored daily for any signs of discomfort. If any animal would show evidence of adverse effects that was greater than minor and transient or in any way compromises its natural behaviour, then it would immediately be killed to prevent suffering, but this did not occur. At eight weeks post-gavage, the experiment was terminated by euthanizing the mice by the CO_2_ inhalation method. The outcomes measured thereafter were alveolar bone loss differences and differentially expressed genes in host and microbial transcriptome.

***Oral swab collection & RNA extraction***

After culling, the oral cavity of each mouse was sampled thrice for 30 seconds each, using sterile fine tip rayon swabs (VWR International). Each swab was placed in a tube containing 100μl of RNA Protect (Qiagen) for transportation to the laboratory. The tubes containing the murine oral swabs were centrifuged at 13,300 g for 10 minutes at room temperature. The swabs were aseptically removed with sterile forceps and the solution was recentrifuged as before for a further 10 mins. Following complete removal of the preservative, the pellets from the three swabs of each mouse were combined using the RNA lysis reagent and total RNA was isolated using *Quick*-RNA™ Fungal/Bacterial Microprep Kit from Zymo Research (Cambridge Biosciences). This incorporated bead beating (with FastPrep 24 MP Biomedicals; set 6 for 40 seconds), initial depletion of chromosomal DNA, and on-column DNAase I treatment steps. Total RNA was eluted in a final volume of 15 μl nuclease-free water and stored at -70^o^C. Negative controls included a kit extraction blank and an environmental control, where a swab was held open in the environment of the animal facility for 30 seconds. RNA concentrations were measured using the Qubit RNA Broad Range Assay Kit (Thermofisher Scientific).

***Metatranscriptomic sequencing & bioinformatic analysis***

Metatranscriptomic library preparation and RNA sequencing were performed by the sequencing service at FISABIO (Valencia, Spain) using NextSeq Illumina Technology (single pair, High input x150 bp) as previously described (Carda-Diéguez et al. 2021). Sequencing reads were trimmed by quality and length using the PRINSEQ program (Schmieder and Edwards 2011). Mice host and microbial ribosomal sequences were identified and separated by aligning the sequence dataset to the *Mus musculus* genome GRCm38.p6 (Accession No. GCA_000001635.9) and SILVA database (Quast et al. 2012), respectively, using Bowtie2 (Langmead and Salzberg 2012). Remaining reads were considered to be mRNA reads, and host and microbial mRNA reads were used separately for further annotation and analyses. In order to properly annotate mRNA reads from the mouse oral bacterial population, we constructed a reference database using the genomes from the Mouse Oral Microbiome Database (MOMD) (<https://momd.org/>) (Joseph et al. 2021). This MOMD genome database comprised of 53 mouse oral bacterial genomes. We assigned a KEGG model to open reading frames (ORFs) by aligning the amino acid dataset to the KEGG database (Kanehisa 2004) with HMMER (Eddy 2011). The abundance of a fragment was equal to the number of sequences aligned into this fragment using Bowtie2 (Langmead and Salzberg 2012). A table of RPKM (Reads per Kbp of transcript per million [Mbp] of mapped reads) values was then generated by normalizing the abundance based on the length of the gene (Kbp) and the size of the dataset (Mbp). Comparative analyses were performed with the significance level of p<0.05 (p-value). Genes of interest that showed a tendency (p<0.1) were also taken into consideration. Only those genes that had a fold change >2 were considered for discussion. Due to the nature of the data and the sample groupings, sample blinding was not possible for this statistical analysis.

For the downstream analysis of the differentially expressed genes, all 24 samples were analyzed for the host gene set while only 23 samples (11 controls and 12 diseased) were used for the analysis of the bacterial gene set, after the detection of an extreme outlier in the CCA analysis. The host genes that were differentially expressed between health and disease were classified into protein classes and gene ontologies of molecular functions and biological processes using the “Gene List Analysis” option in the Panther Classification System (v16.0) (Thomas 2003). Gene ontology enrichment analysis on the list of differentially expressed genes was carried out using the Enrichr database (Chen et al. 2013). Enrichr uses a combination of deviation z-scores and p-values to calculate a combined score value for each gene ontology based on which they are ranked, and we applied an arbitrary threshold of 12 for the combined scores to generate a final list of highly enriched ontologies and related genes. Bacterial gene ontology network analysis was performed using Revigo (Supek et al. 2011) and visualized in Cytoscape v.3.9.0 (Shannon et al. 2003)

Principal component and canonical correspondence correlation analyses were performed in order to display differences between the groups using the R package vegan (Oksanen et al. 2015). DESeq2 test was used to calculate the significance of differences in the transcripts between the health and disease groups (Love et al. 2014). Rarefaction curves, diversity and richness analyses were performed with the same number of reads per sample and results were plotted using vegan.

The raw RNA sequencing reads have been uploaded to the SRA database (Accession number PRJNA807134) and will be publicly available as of the date of publication.

***Periodontal bone loss determination***

Mice were euthanized, mandibles and maxillae were dissected, defleshed and assessed under a Stemi SV11 dissecting microscope (Zeiss) at 25x magnification. The captured images were analysed by ImageJ software (National Institute of Health, USA). The distance from the cemento-enamel junction (CEJ) to the alveolar bone crest (ABC) was measured at 6 pre-determined points (mesio-, mid- and distal-) on both buccal and lingual/palatal surfaces of mandibular and maxillary molar teeth. We have used 2-D imaging for determining the alveolar bone loss levels here which we have used in our previous works as well (Hajishengallis et al. 2011; Payne et al. 2019; Hashim et al. 2021), though we acknowledge that micro computed tomography (micro-CT) is now the more preferred and gold standard technique being used.

In order to calculate bone loss, the mean CEJ-ABC distance from each test mouse was subtracted from the mean CEJ-ABC distance of the control mice, which was used a zero baseline (Baker et al. 2000). The raw mean bone loss values for each animal have been presented in Appendix Table 8. Bone levels between different experimental groups were compared using one-way analysis of variance (ANOVA) and unpaired Student’s t-test between 2 comparison groups. Significance was expressed at the p<0.0005 level. The statistical analyses and graphical visualization of the bone levels were performed using Graphpad Prism 9 (Graphpad Software Inc.). This analysis included eight randomly selected samples from each group. The researcher (A.A) who conducted the periodontal bone loss assessment was not involved in the randomisation of mice for group allocation or in the experimental procedure.

**APPENDIX RESULTS**

The Illumina Next-Seq sequencing runs of the total RNA samples from the mice oral swabs generated an average of 4.2x10^7^ ± 3.2x10^7^ reads for the 24 metatranscriptomic libraries sequenced in this study. After adapter removal and quality control filtering, a mean of 4.1x10^7^ ± 3.1x10^7^ reads per sample were obtained.

Rarefaction curves showed that the murine transcriptome sequencing depth was adequate for all the samples, although the rarefaction curves did not flatten after 3 million reads (Appendix Figure 7A). One of the samples covered 16% of the sequenced transcripts whereas the remaining 23 samples covered 37% suggesting an insufficient sequencing coverage for that sample. All samples showed a complete coverage for the bacterial genes, shown by asymptotic curves after 12,000 reads (Appendix Figure 7B). Neither murine transcripts nor bacterial genes showed significant differences in diversity or richness indexes between health and disease.

Since the 24 samples were obtained from two rounds of experiments and sequencing runs, we also performed the CCA analysis to check for the possible influence of a batch effect on our results. The first batch consisted of eight samples (4 control and 4 disease) while the second batch comprised of the remaining 16 samples (8 control and 8 disease). CCA analysis confirmed the absence of the batch effect in the separation of the samples (Appendix Figure 8A) while the clear separation of health and diseased samples could be observed in the individual batch groups as well (Appendix Figures 8B & C). As with the host gene dataset, we also confirmed the absence of a batch effect in the bacterial gene dataset (Appendix Figure 4B) and the separation of the health and disease groups in individual batches of samples (Appendix Figure 4C & 4D).

We also further characterized the top three most prevalent biological processes listed in Figure 2, namely, cellular process, metabolic process and biological regulation. The top twenty most enriched gene ontologies in each type, in both health and disease, have been listed in Appendix Table 6.

There was a significantly greater impact of the development of periodontal disease on the mouse host transcriptome compared to bacterial gene population, with a more than ten-fold greater number of genes differentially expressed in the host compared to the microbiome (Figures 2 & 6). This likely reflects the simplicity of the mouse oral microbiome with only 4-5 major component bacterial species (Joseph et al. 2021) which should therefore result in a far smaller gene pool compared to the *Mus musculus* host genome.

**APPENDIX DISCUSSION**

Another gene of interest in the list of genes associated with enriched immune-related pathways is *Itgbl1* (integrin, beta-like 1), also over-represented in disease with the highest fold change of 630.84 in comparison to healthy samples – its encoded protein is involved in functions such as cell adhesion, proliferation and differentiation (Miranti and Brugge 2002) and the gene has also been identified as a potential candidate biomarker in an in silico study investigating the association of periodontal disease with Alzheimer’s disease (Jiang et al. 2021).

The list also includes two genes previously reported to have been involved in inflammatory regulation in periodontal disease - *Tnfaip3* (tumor necrosis factor, alpha-induced protein 3) and *Xiap* (X-linked inhibitor of apoptosis), the former through its interactions with Th17 cell differentiation activity (Huang et al. 2021) while the latter encodes for a cleaved caspase-3 inhibitor involved in the inhibition of apoptosis in disease and thus aiding the extended survival of inflammatory cells (Lucas et al. 2010).

Other genes of relevance included *Irf4* (interferon regulatory factor 4) which is involved in regulation of IL-2 production and has been identified as a candidate gene in expression studies of periodontal tissues as well as associated with other systemic disorders such as rheumatoid arthritis and inflammatory bowel disease (Davanian et al. 2012). The gene *Ciita* which encodes for a major histocompatibility complex class II transactivator, was also over-represented in disease and has been previously associated with periodontal bone resorption by influencing the expression and activity of osteoblasts (Lee et al. 2010) – it was also identified to be differentially expressed in periodontal disease in the single-cell gene expression study of the human oral mucosa by (Williams et al. 2021).

The top candidate in the list of enriched molecular function gene ontologies (Appendix Table 7) was – “neuroligin family protein binding” associated with the neurexin gene cluster, *NRXN1, NRXN2* and *NRXN3*. These genes were also associated with some of the enriched biological processes in our dataset including postsynaptic density assembly, protein localization to synapse, imitative and vocal learning (Appendix Table 3). They have also been reported in genome-wide studies to be linked with oral inflammatory conditions such as gingivitis and periodontitis (Garzón et al. 2012; Zhang et al. 2016).

**APPENDIX REFERENCES**

Baker PJ, Dixon M, Evans RT, Roopenian DC. 2000. Heterogeneity of Porphyromonas gingivalis strains in the induction of alveolar bone loss in mice. Oral Microbiol Immunol. 15(1):27–32.

Carda-Diéguez M, Rosier BT, Lloret S, Llena C, Mira A. 2021. The tongue biofilm metatranscriptome identifies metabolic pathways associated with halitosis and its prevention. Dentistry and Oral Medicine. [accessed 2022 Jun 23]. http://medrxiv.org/lookup/doi/10.1101/2021.11.09.21266067.

Chen EY, Tan CM, Kou Y, Duan Q, Wang Z, Meirelles GV, Clark NR, Ma’ayan A. 2013. Enrichr: interactive and collaborative HTML5 gene list enrichment analysis tool. BMC Bioinformatics. 14(1):128.

Davanian H, Stranneheim H, Båge T, Lagervall M, Jansson L, Lundeberg J, Yucel-Lindberg T. 2012. Gene expression profiles in paired gingival biopsies from periodontitis-affected and healthy tissues revealed by massively parallel sequencing. PLoS One. 7(9):e46440.

Eddy SR. 2011. Accelerated Profile HMM Searches. Pearson WR, editor. PLoS Comput Biol. 7(10):e1002195.

Faul F, Erdfelder E, Lang A-G, Buchner A. 2007. G*Power 3: A flexible statistical power analysis program for the social, behavioral, and biomedical sciences. Behavior Research Methods. 39(2):175–191.

Garzón I, Roa A, Moreu G, Oliveira AC, Roda O, Alfonso-Rodríguez CA, González-Jaranay M, Sánchez-Quevedo M del C, Alaminos M. 2012. Development of a diagnostic algorithm in periodontal disease and identification of genetic expression patterns: A preliminary report. Journal of Dental Sciences. 7(1):48–56.

Hajishengallis G, Liang S, Payne MA, Hashim A, Jotwani R, Eskan MA, McIntosh ML, Alsam A, Kirkwood KL, Lambris JD, et al. 2011. Low-Abundance Biofilm Species Orchestrates Inflammatory Periodontal Disease through the Commensal Microbiota and Complement. Cell Host & Microbe. 10(5):497–506.

Hashim A, Alsam A, Payne MA, Aduse-Opoku J, Curtis MA, Joseph S. 2021. Loss of Neutrophil Homing to the Periodontal Tissues Modulates the Composition and Disease Potential of the Oral Microbiota. Whiteley M, editor. Infect Immun. 89(12):e00309-21.

Huang N, Dong H, Luo Y, Shao B. 2021. Th17 Cells in Periodontitis and Its Regulation by A20. Front Immunol. 12:742925.

Jiang Z, Shi Y, Zhao W, Zhou L, Zhang B, Xie Y, Zhang Y, Tan G, Wang Z. 2021. Association between chronic periodontitis and the risk of Alzheimer’s disease: combination of text mining and GEO dataset. BMC Oral Health. 21(1):466.

Joseph S, Aduse-Opoku J, Hashim A, Hanski E, Streich R, Knowles SCL, Pedersen AB, Wade WG, Curtis MA. 2021. A 16S rRNA Gene and Draft Genome Database for the Murine Oral Bacterial Community. Segata N, editor. mSystems. 6(1). [accessed 2021 Oct 4]. https://journals.asm.org/doi/10.1128/mSystems.01222-20.

Kanehisa M. 2004. The KEGG resource for deciphering the genome. Nucleic Acids Research. 32(90001):277D – 280.

Langmead B, Salzberg SL. 2012. Fast gapped-read alignment with Bowtie 2. Nat Methods. 9(4):357–359.

Lee Y-L, Lin S-K, Hong C-Y, Wang J-S, Yang H, Lai EH-H, Chen M-H, Kok S-H. 2010. Major histocompatibility complex class II transactivator inhibits cysteine-rich 61 expression in osteoblastic cells and its implication in the pathogenesis of periapical lesions. J Endod. 36(6):1021–1025.

Love MI, Huber W, Anders S. 2014. Moderated estimation of fold change and dispersion for RNA-seq data with DESeq2. Genome Biol. 15(12):550.

Lucas H, Bartold PM, Dharmapatni A a. SSK, Holding CA, Haynes DR. 2010. Inhibition of apoptosis in periodontitis. J Dent Res. 89(1):29–33.

Miranti CK, Brugge JS. 2002. Sensing the environment: a historical perspective on integrin signal transduction. Nat Cell Biol. 4(4):E83-90.

Oksanen J, Blanchet FG, Kindt R, Legendre P, Minchin PR, O’Hara RB, Simpson GL, Solymos P, Stevens MHH, Wagner H. 2015. vegan: Community Ecology Package.

Payne MA, Hashim A, Alsam A, Joseph S, Aduse-Opoku J, Wade WG, Curtis MA. 2019. Horizontal and Vertical Transfer of Oral Microbial Dysbiosis and Periodontal Disease. J Dent Res. 98(13):1503–1510.

Quast C, Pruesse E, Yilmaz P, Gerken J, Schweer T, Yarza P, Peplies J, Glöckner FO. 2012. The SILVA ribosomal RNA gene database project: improved data processing and web-based tools. Nucleic Acids Research. 41(D1):D590–D596.

Schmieder R, Edwards R. 2011. Quality control and preprocessing of metagenomic datasets. Bioinformatics. 27(6):863–864.

Shannon P, Markiel A, Ozier O, Baliga NS, Wang JT, Ramage D, Amin N, Schwikowski B, Ideker T. 2003. Cytoscape: A Software Environment for Integrated Models of Biomolecular Interaction Networks. Genome Res. 13(11):2498–2504.

Supek F, Bošnjak M, Škunca N, Šmuc T. 2011. REVIGO Summarizes and Visualizes Long Lists of Gene Ontology Terms. Gibas C, editor. PLoS ONE. 6(7):e21800.

Thomas PD. 2003. PANTHER: A Library of Protein Families and Subfamilies Indexed by Function. Genome Research. 13(9):2129–2141.

Williams DW, Greenwell-Wild T, Brenchley L, Dutzan N, Overmiller A, Sawaya AP, Webb S, Martin D, Hajishengallis G, Divaris K, et al. 2021. Human oral mucosa cell atlas reveals a stromal-neutrophil axis regulating tissue immunity. Cell. 184(15):4090-4104.e15.

Zhang S, Divaris K, Moss K, Yu N, Barros S, Marchesan J, Morelli T, Agler C, Kim SJ, Wu D, et al. 2016. The Novel ASIC2 Locus is Associated with Severe Gingival Inflammation. JDR Clin Trans Res. 1(2):163–170.

**APPENDIX FIGURES**

**
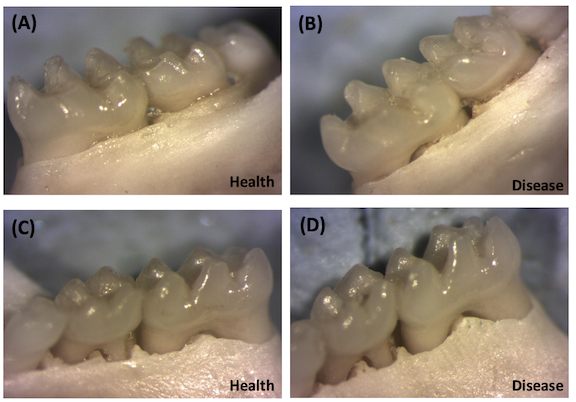
**

**Appendix Figure 1** Representative 2-D section images of the murine teeth used for measuring alveolar bone levels captured under a Stemi SV11 dissecting microscope (Zeiss) at 25x magnification. The distance from the cemento-enamel junction (CEJ) to the alveolar bone crest (ABC) was measured at 6 pre-determined points (mesio-, mid- and distal-) on both buccal and lingual/palatal surfaces of mandibular and maxillary molar teeth. For bone loss calculations, the mean CEJ-ABC distance from each test mouse was subtracted from the mean CEJ-ABC distance of the control mice, which was used a zero baseline

**
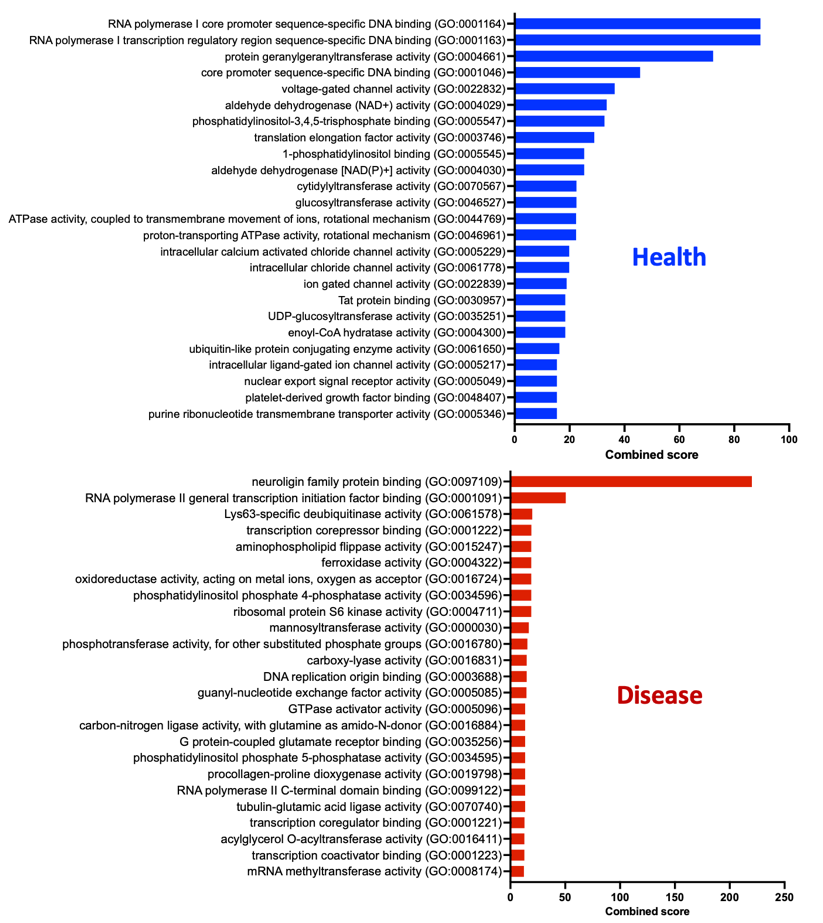
**

**Appendix Figure 2** Top 25 molecular function gene ontologies ranked according to Enrichr calculated combined score in health (blue) and disease (red), derived from the differentially expressed genes in the mouse host oral metatranscriptome

**
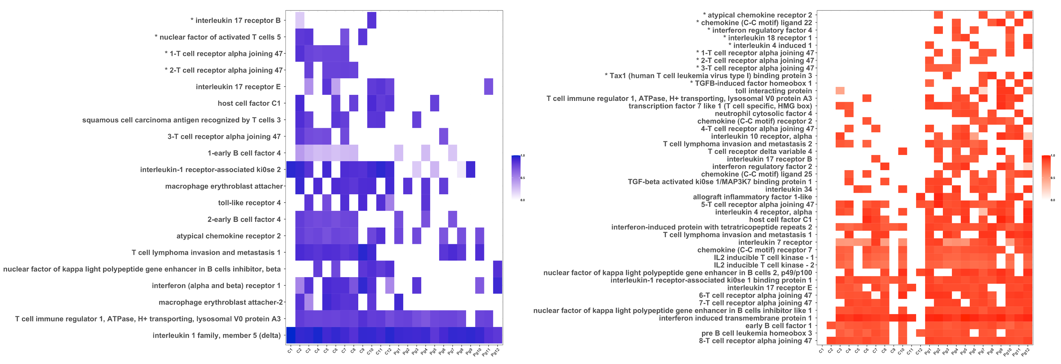
**

**Appendix Figure 3** Heatmap depicting relative gene expression level for a list of immune-related genes from the differentially expressed gene set using keywords such as chemokine, interleukin, B-cell, T-cell, neutrophil and TLRs in health (blue) and disease (red). Samples C1-C12 on the X-axis are related to health, and samples Pg1-12 related to disease. The genes have been listed in decreasing order of fold change and the genes marked with asterisks refer to ones that were present only in the indicated condition and completely absent otherwise.

**
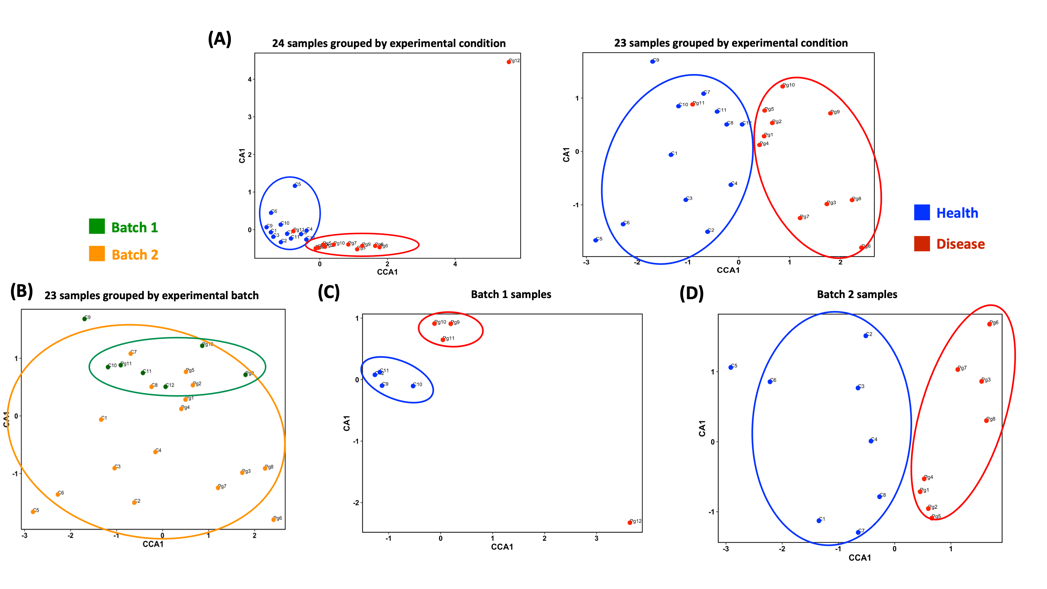
**

**Appendix Figure 4** Canonical coordinate analysis (CCA) plots showing the distribution of bacterial gene expression of the (a) 24 mouse oral samples in health and disease conditions (b) 23 samples excluding the outlier sample Pg12 in health and disease conditions (C) 23 samples grouped by experimental batches (D) Batch 1 mouse samples in health and disease conditions (E) Batch 2 mouse samples in health and disease conditions

**
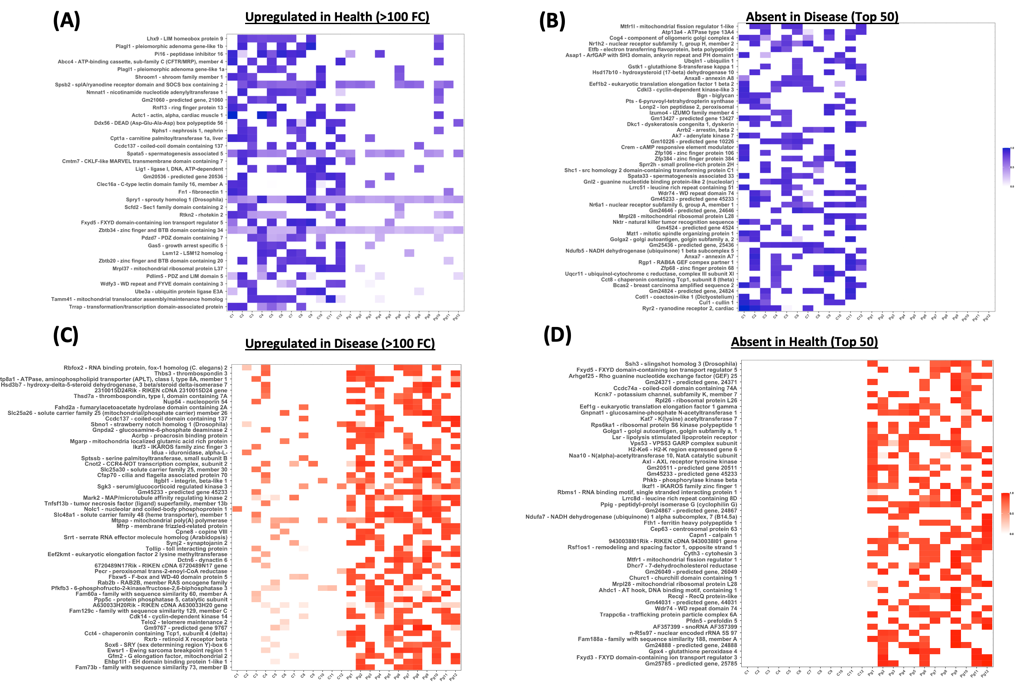
**

**Appendix Figure 5** Heatmaps depicting relative gene expression levels between the 24 mouse oral samples for (A) genes upregulated in health with a FC>100 (B) top 50 genes that were completely absent in disease and only expressed in the health condition (C) genes upregulated in disease condition with a FC>100 (D) top 50 genes that were completely absent in health and only expressed in the disease condition. Samples C1-C12 on the X-axis are related to health, and samples Pg1-12 related to disease.

**
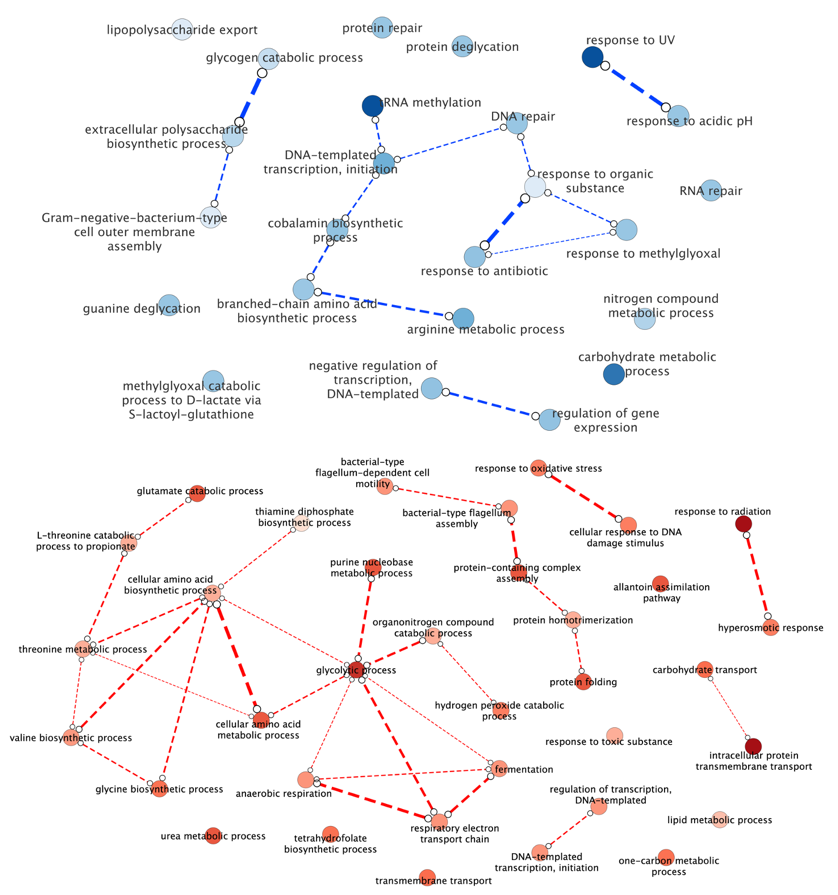
**

**Appendix Figure 6** Revigo bacterial gene ontology networks of the biological processes in health (blue) and disease (red) linked to the differentially expressed bacterial genes. The light to dark shades of the bubble colours correspond to the effect size of the fold change in gene expression. Highly similar GO terms are linked by dashed edges, where the line width indicates the degree of similarity.

**
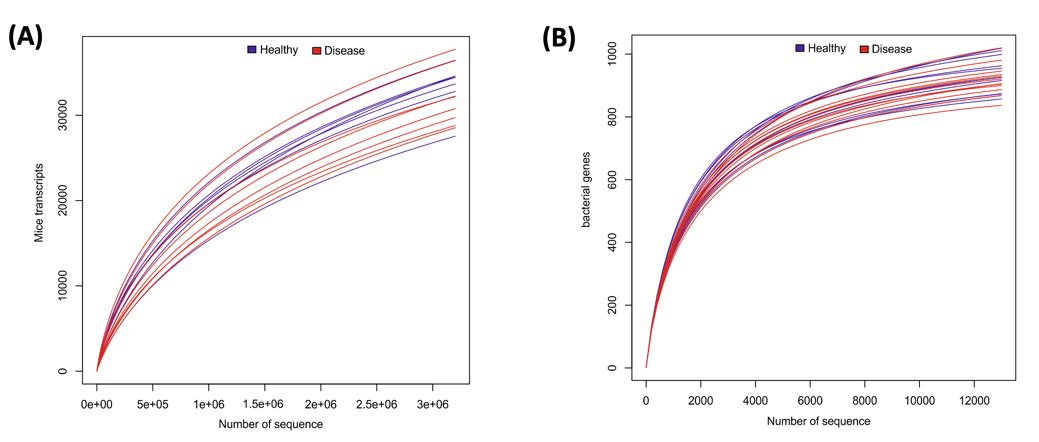
**

**Appendix Figure 7** Rarefaction curves demonstrating the extent of sequencing depths obtained for (A) the murine host transcripts and (B) murine oral bacterial genes in health (blue) and disease (red)

**
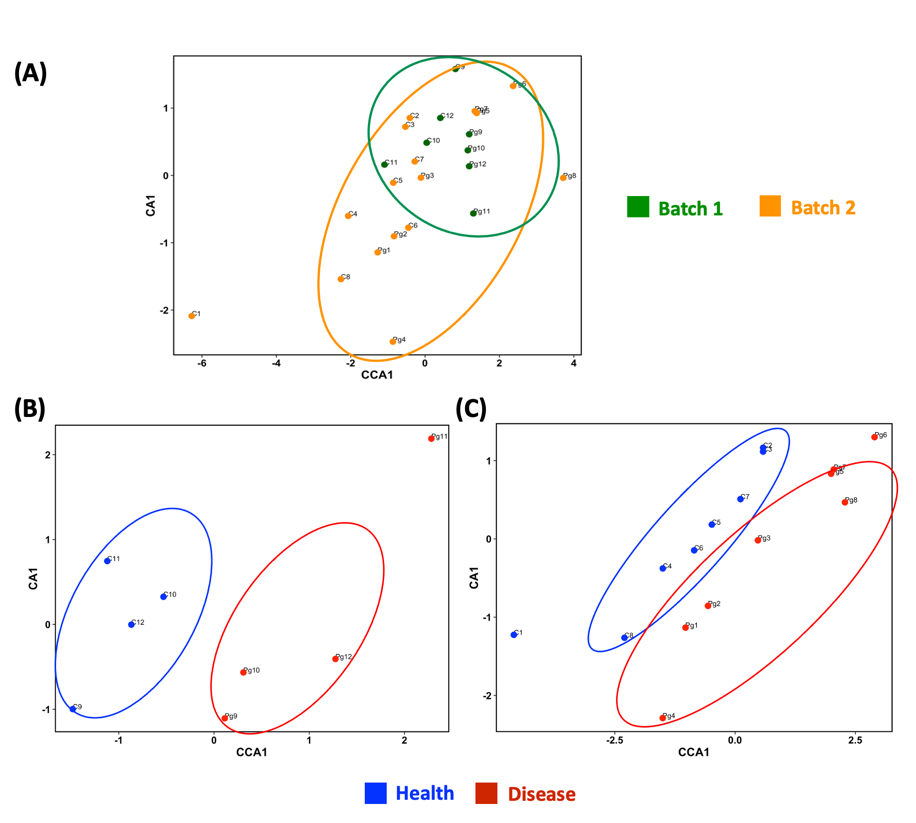
**

**Appendix Figure 8** Canonical coordinate analysis (CCA) plots showing the distribution of the 24 mouse host oral samples gene expression based on (A) experimental batch (B) health and disease in batch 1 (C) health and disease in batch 2

**Appendix Table 1** Top 100 most differentially expressed genes (based on fold change) in health (blue) and disease (red) respectively in the mouse host oral metatranscriptome with a FC>2 (log2FC>1), p<0.01 and effect size > 0.5

| **Gene name** | **Gene description** | **log2FC** | **Upregulated Condition** |
| --- | --- | --- | --- |
| Ryr2 | ryanodine receptor 2, cardiac | 38.37 | Health |
| Cul1 | cullin 1 | 36.71 | Health |
| Cotl1 | coactosin-like 1 (Dictyostelium) | 36.58 | Health |
| Gm24824 | predicted gene, 24824 | 36.50 | Health |
| Bcas2 | breast carcinoma amplified sequence 2 | 36.40 | Health |
| Cct8 | chaperonin containing Tcp1, subunit 8 (theta) | 36.35 | Health |
| Uqcr11 | ubiquinol-cytochrome c reductase, complex III subunit XI | 36.15 | Health |
| Zfp68 | zinc finger protein 68 | 36.14 | Health |
| Rgp1 | RAB6A GEF compex partner 1 | 36.13 | Health |
| Anxa7 | annexin A7 | 36.08 | Health |
| Ndufb5 | NADH dehydrogenase (ubiquinone) 1 beta subcomplex, 5 | 35.98 | Health |
| Gm25436 | predicted gene, 25436 | 35.91 | Health |
| Golga2 | golgi autoantigen, golgin subfamily a, 2 | 35.88 | Health |
| Mzt1 | mitotic spindle organizing protein 1 | 35.83 | Health |
| Gm4524 | predicted gene 4524 | 35.80 | Health |
| Nktr | natural killer tumor recognition sequence | 35.71 | Health |
| Mrpl28 | mitochondrial ribosomal protein L28 | 35.70 | Health |
| Gm24646 | predicted gene, 24646 | 35.69 | Health |
| Nr6a1 | nuclear receptor subfamily 6, group A, member 1 | 35.64 | Health |
| Gm45233 | predicted gene 45233 | 35.63 | Health |
| Wdr74 | WD repeat domain 74 | 35.59 | Health |
| Lrrc51 | leucine rich repeat containing 51 | 35.54 | Health |
| Gnl2 | guanine nucleotide binding protein-like 2 (nucleolar) | 35.53 | Health |
| Spata33 | spermatogenesis associated 33 | 35.47 | Health |
| Shc1 | src homology 2 domain-containing transforming protein C1 | 35.45 | Health |
| Sprr2h | small proline-rich protein 2H | 35.40 | Health |
| Zfp384 | zinc finger protein 384 | 35.40 | Health |
| Zfp106 | zinc finger protein 106 | 35.35 | Health |
| Crem | cAMP responsive element modulator | 35.31 | Health |
| Gm10226 | predicted gene 10226 | 35.25 | Health |
| Ak7 | adenylate kinase 7 | 35.20 | Health |
| Arrb2 | arrestin, beta 2 | 35.19 | Health |
| Dkc1 | dyskeratosis congenita 1, dyskerin | 35.19 | Health |
| Gm13427 | predicted gene 13427 | 35.16 | Health |
| Izumo4 | IZUMO family member 4 | 35.14 | Health |
| Lonp2 | lon peptidase 2, peroxisomal | 35.13 | Health |
| Pts | 6-pyruvoyl-tetrahydropterin synthase | 35.10 | Health |
| Bgn | biglycan | 35.08 | Health |
| Cdkl3 | cyclin-dependent kinase-like 3 | 35.05 | Health |
| Eef1b2 | eukaryotic translation elongation factor 1 beta 2 | 35.02 | Health |
| Anxa8 | annexin A8 | 35.02 | Health |
| Hsd17b10 | hydroxysteroid (17-beta) dehydrogenase 10 | 34.97 | Health |
| Gstk1 | glutathione S-transferase kappa 1 | 34.95 | Health |
| Ubqln1 | ubiquilin 1 | 34.83 | Health |
| Asap1 | ArfGAP with SH3 domain, ankyrin repeat and PH domain1 | 34.81 | Health |
| Etfb | electron transferring flavoprotein, beta polypeptide | 34.76 | Health |
| Nr1h2 | nuclear receptor subfamily 1, group H, member 2 | 34.76 | Health |
| Cog4 | component of oligomeric golgi complex 4 | 34.75 | Health |
| Atp13a4 | ATPase type 13A4 | 34.74 | Health |
| Mtfr1l | mitochondrial fission regulator 1-like | 34.74 | Health |
| Plgrkt | plasminogen receptor, C-terminal lysine transmembrane protein | 34.73 | Health |
| Aldh9a1 | aldehyde dehydrogenase 9, subfamily A1 | 34.69 | Health |
| Accs | 1-aminocyclopropane-1-carboxylate synthase (non-functional) | 34.69 | Health |
| Lysmd3 | LysM, putative peptidoglycan-binding, domain containing 3 | 34.68 | Health |
| Gria1 | glutamate receptor, ionotropic, AMPA1 (alpha 1) | 34.68 | Health |
| Prr3 | proline-rich polypeptide 3 | 34.65 | Health |
| Foxp1 | forkhead box P1 | 34.62 | Health |
| Tfap2b | transcription factor AP-2 beta | 34.62 | Health |
| Lmbr1 | limb region 1 | 34.61 | Health |
| Epg5 | ectopic P-granules autophagy protein 5 homolog (C. elegans) | 34.55 | Health |
| Epg5 | ectopic P-granules autophagy protein 5 homolog (C. elegans) | 34.55 | Health |
| Wdr45 | WD repeat domain 45 | 34.55 | Health |
| Olfr1445 | olfactory receptor 1445 | 34.55 | Health |
| Mrpl22 | mitochondrial ribosomal protein L22 | 34.52 | Health |
| Adrbk1 | adrenergic receptor kinase, beta 1 | 34.50 | Health |
| Slc35b2 | solute carrier family 35, member B2 | 34.47 | Health |
| Gm36520 | predicted gene, 36520 | 34.45 | Health |
| Dda1 | DET1 and DDB1 associated 1 | 34.39 | Health |
| Lsm5 | LSM5 homolog, U6 small nuclear RNA and mRNA degradation associated | 34.37 | Health |
| Cbx1 | chromobox 1 | 34.37 | Health |
| Cuedc2 | CUE domain containing 2 | 34.37 | Health |
| Peli2 | pellino 2 | 34.35 | Health |
| Gm15706 | predicted gene 15706 | 34.32 | Health |
| Efcab14 | EF-hand calcium binding domain 14 | 34.26 | Health |
| Cr1l | complement component (3b/4b) receptor 1-like | 34.25 | Health |
| Kxd1 | KxDL motif containing 1 | 34.23 | Health |
| Klc2 | kinesin light chain 2 | 34.21 | Health |
| Igkv20-101-2 | immunoglobulin kappa chain variable 20-101-2 | 34.20 | Health |
| AW551984 | expressed sequence AW551984 | 34.19 | Health |
| Dram2 | DNA-damage regulated autophagy modulator 2 | 34.18 | Health |
| Gm20492 | predicted gene 20492 | 34.18 | Health |
| Suv420h2 | suppressor of variegation 4-20 homolog 2 (Drosophila) | 34.15 | Health |
| Sugt1 | SGT1, suppressor of G2 allele of SKP1 (S. cerevisiae) | 34.13 | Health |
| Rps6kb2 | ribosomal protein S6 kinase, polypeptide 2 | 34.13 | Health |
| Rps6kb2 | ribosomal protein S6 kinase, polypeptide 2 | 34.13 | Health |
| Gm3409 | predicted gene 3409 | 34.09 | Health |
| Gm45218 | predicted gene 45218 | 34.09 | Health |
| Ftx | Ftx transcript, Xist regulator (non-protein coding) | 34.08 | Health |
| Gm45020 | predicted gene 45020 | 34.05 | Health |
| Pantr1 | POU domain, class 3, transcription factor 3 adjacent noncoding transcript 1 | 34.04 | Health |
| Tbk1 | TANK-binding kinase 1 | 34.04 | Health |
| Gm9991 | predicted gene 9991 | 34.04 | Health |
| Dda1 | DET1 and DDB1 associated 1 | 34.03 | Health |
| Thpo | thrombopoietin | 34.02 | Health |
| BC026585 | cDNA sequence BC026585 | 33.99 | Health |
| Ubr5 | ubiquitin protein ligase E3 component n-recognin 5 | 33.96 | Health |
| Fn1 | fibronectin 1 | 33.95 | Health |
| Pggt1b | protein geranylgeranyltransferase type I, beta subunit | 33.93 | Health |
| Plaur | plasminogen activator, urokinase receptor | 33.92 | Health |
| Tmbim1 | transmembrane BAX inhibitor motif containing 1 | 33.91 | Health |
| Gm7325 | predicted gene 7325 | 33.88 | Health |
| Cd164l2 | CD164 sialomucin-like 2 | -34.07 | Disease |
| Gm13727 | predicted gene 13727 | -34.07 | Disease |
| Tfg | Trk-fused gene | -34.08 | Disease |
| Tpr | translocated promoter region, nuclear basket protein | -34.09 | Disease |
| Trmt6 | tRNA methyltransferase 6 | -34.09 | Disease |
| Sipa1l1 | signal-induced proliferation-associated 1 like 1 | -34.11 | Disease |
| Nat6 | N-acetyltransferase 6 | -34.11 | Disease |
| 4930523C07Rik | RIKEN cDNA 4930523C07 gene | -34.11 | Disease |
| Gm26569 | predicted gene, 26569 | -34.11 | Disease |
| Cox6b2 | cytochrome c oxidase subunit VIb polypeptide 2 | -34.13 | Disease |
| Snw1 | SNW domain containing 1 | -34.14 | Disease |
| Zbtb8os | zinc finger and BTB domain containing 8 opposite strand | -34.18 | Disease |
| Nipsnap1 | nipsnap homolog 1 (C. elegans) | -34.18 | Disease |
| Ppp2r1b | protein phosphatase 2, regulatory subunit A, beta | -34.19 | Disease |
| Mrpl46 | mitochondrial ribosomal protein L46 | -34.20 | Disease |
| Itgb1 | integrin beta 1 (fibronectin receptor beta) | -34.21 | Disease |
| Eng | endoglin | -34.21 | Disease |
| Map4 | microtubule-associated protein 4 | -34.22 | Disease |
| Stxbp2 | syntaxin binding protein 2 | -34.22 | Disease |
| Tpd52l2 | tumor protein D52-like 2 | -34.25 | Disease |
| Fam189b | family with sequence similarity 189, member B | -34.25 | Disease |
| Rpl13a-ps1 | ribosomal protein 13A, pseudogene 1 | -34.25 | Disease |
| Ccdc40 | coiled-coil domain containing 40 | -34.26 | Disease |
| Cnot7 | CCR4-NOT transcription complex, subunit 7 | -34.26 | Disease |
| Ube2s | ubiquitin-conjugating enzyme E2S | -34.27 | Disease |
| Carkd | carbohydrate kinase domain containing | -34.28 | Disease |
| Rps6ka3 | ribosomal protein S6 kinase polypeptide 3 | -34.30 | Disease |
| 0610010K14Rik | RIKEN cDNA 0610010K14 gene | -34.30 | Disease |
| Scrn2 | secernin 2 | -34.31 | Disease |
| Mthfd2 | methylenetetrahydrofolate dehydrogenase (NAD+ dependent), methenyltetrahydrofolate cyclohydrolase | -34.33 | Disease |
| Dpp4 | dipeptidylpeptidase 4 | -34.34 | Disease |
| Arl5a | ADP-ribosylation factor-like 5A | -34.35 | Disease |
| Uqcrc2 | ubiquinol cytochrome c reductase core protein 2 | -34.39 | Disease |
| Dctd | dCMP deaminase | -34.40 | Disease |
| Timm9 | translocase of inner mitochondrial membrane 9 | -34.40 | Disease |
| Pot1a | protection of telomeres 1A | -34.42 | Disease |
| Sdhaf2 | succinate dehydrogenase complex assembly factor 2 | -34.43 | Disease |
| Tpra1 | transmembrane protein, adipocyte asscociated 1 | -34.43 | Disease |
| Pex12 | peroxisomal biogenesis factor 12 | -34.45 | Disease |
| Tax1bp3 | Tax1 (human T cell leukemia virus type I) binding protein 3 | -34.46 | Disease |
| Pgm1 | phosphoglucomutase 1 | -34.47 | Disease |
| Gtf2ird1 | general transcription factor II I repeat domain-containing 1 | -34.48 | Disease |
| Zfp236 | zinc finger protein 236 | -34.51 | Disease |
| Spag5 | sperm associated antigen 5 | -34.52 | Disease |
| Ciz1 | CDKN1A interacting zinc finger protein 1 | -34.55 | Disease |
| Arl6ip4 | ADP-ribosylation factor-like 6 interacting protein 4 | -34.56 | Disease |
| Churc1 | churchill domain containing 1 | -34.56 | Disease |
| Scnm1 | sodium channel modifier 1 | -34.57 | Disease |
| Fbxw2 | F-box and WD-40 domain protein 2 | -34.58 | Disease |
| Atxn7l1 | ataxin 7-like 1 | -34.58 | Disease |
| Syngr2 | synaptogyrin 2 | -34.63 | Disease |
| Ssh3 | slingshot homolog 3 (Drosophila) | -34.63 | Disease |
| Fxyd5 | FXYD domain-containing ion transport regulator 5 | -34.66 | Disease |
| Arhgef25 | Rho guanine nucleotide exchange factor (GEF) 25 | -34.67 | Disease |
| Gm24371 | predicted gene, 24371 | -34.68 | Disease |
| Ccdc74a | coiled-coil domain containing 74A | -34.69 | Disease |
| Kcnk7 | potassium channel, subfamily K, member 7 | -34.72 | Disease |
| Rpl26 | ribosomal protein L26 | -34.75 | Disease |
| Eef1g | eukaryotic translation elongation factor 1 gamma | -34.75 | Disease |
| Gnpnat1 | glucosamine-phosphate N-acetyltransferase 1 | -34.75 | Disease |
| Kat7 | K(lysine) acetyltransferase 7 | -34.78 | Disease |
| Rps6ka1 | ribosomal protein S6 kinase polypeptide 1 | -34.80 | Disease |
| Golga1 | golgi autoantigen, golgin subfamily a, 1 | -34.83 | Disease |
| Lsr | lipolysis stimulated lipoprotein receptor | -34.87 | Disease |
| Vps53 | VPS53 GARP complex subunit | -34.88 | Disease |
| H2-Ke6 | H2-K region expressed gene 6 | -34.90 | Disease |
| Naa10 | N(alpha)-acetyltransferase 10, NatA catalytic subunit | -34.93 | Disease |
| Axl | AXL receptor tyrosine kinase | -34.96 | Disease |
| Gm20511 | predicted gene 20511 | -34.97 | Disease |
| Gm45233 | predicted gene 45233 | -34.98 | Disease |
| Phkb | phosphorylase kinase beta | -35.00 | Disease |
| Ikzf1 | IKAROS family zinc finger 1 | -35.04 | Disease |
| Rbms1 | RNA binding motif, single stranded interacting protein 1 | -35.06 | Disease |
| Lrrc8d | leucine rich repeat containing 8D | -35.07 | Disease |
| Ppig | peptidyl-prolyl isomerase G (cyclophilin G) | -35.07 | Disease |
| Gm24867 | predicted gene, 24867 | -35.19 | Disease |
| Ndufa7 | NADH dehydrogenase (ubiquinone) 1 alpha subcomplex, 7 (B14.5a) | -35.20 | Disease |
| Fth1 | ferritin heavy polypeptide 1 | -35.21 | Disease |
| Cep63 | centrosomal protein 63 | -35.27 | Disease |
| Capn1 | calpain 1 | -35.27 | Disease |
| 9430038I01Rik | RIKEN cDNA 9430038I01 gene | -35.29 | Disease |
| Rsf1os1 | remodeling and spacing factor 1, opposite strand 1 | -35.30 | Disease |
| Cyth3 | cytohesin 3 | -35.42 | Disease |
| Mtfr1 | mitochondrial fission regulator 1 | -35.43 | Disease |
| Dhcr7 | 7-dehydrocholesterol reductase | -35.49 | Disease |
| Gm26049 | predicted gene, 26049 | -35.54 | Disease |
| Churc1 | churchill domain containing 1 | -35.56 | Disease |
| Mrpl28 | mitochondrial ribosomal protein L28 | -35.56 | Disease |
| Ahdc1 | AT hook, DNA binding motif, containing 1 | -35.60 | Disease |
| Recql | RecQ protein-like | -35.68 | Disease |
| Gm44031 | predicted gene, 44031 | -35.70 | Disease |
| Wdr74 | WD repeat domain 74 | -35.73 | Disease |
| Trappc6a | trafficking protein particle complex 6A | -35.75 | Disease |
| Pfdn5 | prefoldin 5 | -35.83 | Disease |
| AF357399 | snoRNA AF357399 | -35.95 | Disease |
| n-R5s97 | nuclear encoded rRNA 5S 97 | -35.98 | Disease |
| Fam188a | family with sequence similarity 188, member A | -36.00 | Disease |
| Gm24888 | predicted gene, 24888 | -36.39 | Disease |
| Gpx4 | glutathione peroxidase 4 | -36.55 | Disease |
| Fxyd3 | FXYD domain-containing ion transport regulator 3 | -36.89 | Disease |
| Gm25785 | predicted gene, 25785 | -36.92 | Disease |

**Appendix Table 2** Top 500 most expressed oral transcripts in healthy SPF mice, ranked in descending order of gene expression levels.

| **target_id** | **Gene name** | **Gene description** |
| --- | --- | --- |
| ENSMUST00000198477.1 | Unclassified | Unclassified |
| ENSMUST00000217116.1 | Lars2 | leucyl-tRNA synthetase, mitochondrial |
| ENSMUST00000200021.1 | Rtkn2 | rhotekin 2 |
| ENSMUST00000082390.1 | mt-Td | mitochondrially encoded tRNA aspartic acid |
| ENSMUST00000082388.1 | mt-Td | mitochondrially encoded tRNA aspartic acid |
| ENSMUST00000099683.1 | Gm9945 | predicted gene 9945 |
| ENSMUST00000174924.2 | Rn7s1 | 7S RNA 1 |
| ENSMUST00000175032.2 | Rn7s2 | 7S RNA 2 |
| ENSMUST00000045912.2 | Rptn | repetin |
| ENSMUST00000007275.2 | Krt13 | keratin 13 |
| ENSMUST00000023797.7 | Krt4 | keratin 4 |
| ENSMUST00000172812.2 | Malat1 | metastasis associated lung adenocarcinoma transcript 1 (non-coding RNA) |
| ENSMUST00000090856.9 | Fam19a3 | family with sequence similarity 19, member A3 |
| ENSMUST00000083103.1 | Rn7sk | RNA, 7SK, nuclear |
| ENSMUST00000100179.1 | Krt76 | keratin 76 |
| ENSMUST00000182010.1 | Gm42413 | predicted gene, 42413 |
| ENSMUST00000192833.1 | Gm26916 | predicted gene, 26916 |
| ENSMUST00000093209.3 | Hba-a1 | hemoglobin alpha, adult chain 1 |
| ENSMUST00000181242.1 | Gm26868 | predicted gene, 26868 |
| ENSMUST00000092956.3 | Dhrs3 | dehydrogenase/reductase (SDR family) member 3 |
| ENSMUST00000023934.7 | Gm26505 | predicted gene, 26505 |
| ENSMUST00000026499.5 | Crisp3 | cysteine-rich secretory protein 3 |
| ENSMUST00000093207.3 | Hba-a1 | hemoglobin alpha, adult chain 1 |
| ENSMUST00000103131.4 | Krt10 | keratin 10 |
| ENSMUST00000173672.1 | Neat1 | nuclear paraspeckle assembly transcript 1 (non-protein coding) |
| ENSMUST00000081399.3 | Klk1b9 | kallikrein 1-related peptidase b9 |
| ENSMUST00000023788.7 | Krt6a | keratin 6A |
| ENSMUST00000124830.2 | Enc1 | ectodermal-neural cortex 1 |
| ENSMUST00000048945.5 | Klk1b27 | kallikrein 1-related peptidase b27 |
| ENSMUST00000175096.1 | Rpph1 | ribonuclease P RNA component H1 |
| ENSMUST00000042235.14 | Phlda1 | pleckstrin homology like domain, family A, member 1 |
| ENSMUST00000181957.1 | Gm26868 | predicted gene, 26868 |
| ENSMUST00000023786.6 | Epn2 | epsin 2 |
| ENSMUST00000007280.8 | Krt16 | keratin 16 |
| ENSMUST00000082402.1 | mt-Nd6 | mitochondrially encoded NADH dehydrogenase 6 |
| ENSMUST00000099046.3 | Gm10837 | predicted gene 10837 |
| ENSMUST00000099684.3 | Gm9945 | predicted gene 9945 |
| ENSMUST00000077528.6 | Klk1b22 | kallikrein 1-related peptidase b22 |
| ENSMUST00000077146.3 | Drd5 | dopamine receptor D5 |
| ENSMUST00000035672.4 | Ppl | periplakin |
| ENSMUST00000043400.7 | Asprv1 | aspartic peptidase, retroviral-like 1 |
| ENSMUST00000085450.3 | Klk1b3 | kallikrein 1-related peptidase b3 |
| ENSMUST00000164932.2 | Krt78 | keratin 78 |
| ENSMUST00000110299.2 | Tgm3 | transglutaminase 3, E polypeptide |
| ENSMUST00000058150.7 | Lor | loricrin |
| ENSMUST00000064257.5 | Tchh | trichohyalin |
| ENSMUST00000044804.7 | Cdsn | corneodesmosin |
| ENSMUST00000082421.1 | mt-Nd6 | mitochondrially encoded NADH dehydrogenase 6 |
| ENSMUST00000069245.7 | Spink5 | serine peptidase inhibitor, Kazal type 5 |
| ENSMUST00000180798.1 | Gm26903 | predicted gene, 26903 |
| ENSMUST00000005933.3 | Klk1b16 | kallikrein 1-related peptidase b16 |
| ENSMUST00000017255.3 | Krt24 | keratin 24 |
| ENSMUST00000058142.3 | Sprr3 | small proline-rich protein 3 |
| ENSMUST00000112717.2 | Serpinb3a | serine (or cysteine) peptidase inhibitor, clade B (ovalbumin), member 3A |
| ENSMUST00000100497.10 | Actb | actin, beta |
| ENSMUST00000082408.1 | Mybl1 | myeloblastosis oncogene-like 1 |
| ENSMUST00000072363.4 | Kprp | keratinocyte expressed, proline-rich |
| ENSMUST00000042219.5 | Calm4 | calmodulin 4 |
| ENSMUST00000188231.1 | Gm28653 | predicted gene 28653 |
| ENSMUST00000020488.8 | D10Wsu102e | DNA segment, Chr 10, Wayne State University 102, expressed |
| ENSMUST00000047864.10 | Eef2 | eukaryotic translation elongation factor 2 |
| ENSMUST00000027667.12 | Pkp1 | plakophilin 1 |
| ENSMUST00000082414.1 | mt-Nd6 | mitochondrially encoded NADH dehydrogenase 6 |
| ENSMUST00000007272.7 | Krt14 | keratin 14 |
| ENSMUST00000082418.1 | mt-Nd6 | mitochondrially encoded NADH dehydrogenase 6 |
| ENSMUST00000109367.9 | Slpi | secretory leukocyte peptidase inhibitor |
| ENSMUST00000086519.11 | Rplp0 | ribosomal protein, large, P0 |
| ENSMUST00000019998.8 | Perp | PERP, TP53 apoptosis effector |
| ENSMUST00000007156.4 | Klk1b11 | kallikrein 1-related peptidase b11 |
| ENSMUST00000098192.3 | Gm26505 | predicted gene, 26505 |
| ENSMUST00000187117.1 | Gm29208 | predicted gene 29208 |
| ENSMUST00000142555.1 | Hba-a1 | hemoglobin alpha, adult chain 1 |
| ENSMUST00000025563.7 | Fth1 | ferritin heavy polypeptide 1 |
| ENSMUST00000076737.6 | Zfp457 | zinc finger protein 457 |
| ENSMUST00000165887.7 | Dmkn | dermokine |
| ENSMUST00000174287.1 | Neat1 | nuclear paraspeckle assembly transcript 1 (non-protein coding) |
| ENSMUST00000193521.1 | 2310046K23Rik | RIKEN cDNA 2310046K23 gene |
| ENSMUST00000017841.3 | Ada | adenosine deaminase |
| ENSMUST00000023709.6 | Krt4 | keratin 4 |
| ENSMUST00000170805.8 | Fetub | fetuin beta |
| ENSMUST00000052126.5 | Fam25c | family with sequence similarity 25, member C |
| ENSMUST00000029046.8 | Fabp5 | fatty acid binding protein 5, epidermal |
| ENSMUST00000085455.5 | Klk1b5 | kallikrein 1-related peptidase b5 |
| ENSMUST00000181572.1 | Gm26868 | predicted gene, 26868 |
| ENSMUST00000157463.1 | Rmrp | RNA component of mitochondrial RNAase P |
| ENSMUST00000127906.7 | Enc1 | ectodermal-neural cortex 1 |
| ENSMUST00000143083.2 | Gm10115 | predicted gene 10115 |
| ENSMUST00000226445.1 | Unclassified | Unclassified |
| ENSMUST00000128934.1 | Krt13 | keratin 13 |
| ENSMUST00000082392.1 | Mtf1 | metal response element binding transcription factor 1 |
| ENSMUST00000072204.4 | Klk1b8 | kallikrein 1-related peptidase b8 |
| ENSMUST00000226781.2 | Pars2 | prolyl-tRNA synthetase (mitochondrial)(putative) |
| ENSMUST00000112724.2 | Serpinb12 | serine (or cysteine) peptidase inhibitor, clade B (ovalbumin), member 12 |
| ENSMUST00000074359.3 | Klk1b5 | kallikrein 1-related peptidase b5 |
| ENSMUST00000045902.12 | Fmo2 | flavin containing monooxygenase 2 |
| ENSMUST00000107300.6 | Crnn | cornulin |
| ENSMUST00000190277.1 | Gm28434 | predicted gene 28434 |
| ENSMUST00000134040.1 | Ubp1 | upstream binding protein 1 |
| ENSMUST00000077698.4 | Calml3 | calmodulin-like 3 |
| ENSMUST00000077354.4 | Klk1b4 | kallikrein 1-related pepidase b4 |
| ENSMUST00000177817.1 | Gm21726 | predicted gene, 21726 |
| ENSMUST00000034966.8 | Rpl4 | ribosomal protein L4 |
| ENSMUST00000038710.5 | Dsc1 | desmocollin 1 |
| ENSMUST00000099049.3 | Dnmt3aos | DNA methyltransferase 3A, opposite strand |
| ENSMUST00000080518.13 | Sik3 | SIK family kinase 3 |
| ENSMUST00000195515.1 | Crnn | cornulin |
| ENSMUST00000001809.14 | Pabpc1 | poly(A) binding protein, cytoplasmic 1 |
| ENSMUST00000123995.7 | Eif4a1 | eukaryotic translation initiation factor 4A1 |
| ENSMUST00000040442.5 | Aox4 | aldehyde oxidase 4 |
| ENSMUST00000077196.5 | Krt80 | keratin 80 |
| ENSMUST00000200624.1 | Gm42921 | predicted gene 42921 |
| ENSMUST00000094014.9 | Fam57a | family with sequence similarity 57, member A |
| ENSMUST00000037007.3 | Evpl | envoplakin |
| ENSMUST00000217356.1 | Rpsa | ribosomal protein SA |
| ENSMUST00000039887.3 | Pof1b | premature ovarian failure 1B |
| ENSMUST00000001592.14 | Jup | junction plakoglobin |
| ENSMUST00000214837.1 | Flg | filaggrin |
| ENSMUST00000024739.13 | Hsp90ab1 | heat shock protein 90 alpha (cytosolic), class B member 1 |
| ENSMUST00000082396.1 | mt-Nd6 | mitochondrially encoded NADH dehydrogenase 6 |
| ENSMUST00000106215.10 | Actc1 | actin, alpha, cardiac muscle 1 |
| ENSMUST00000008036.8 | Cyp39a1 | cytochrome P450, family 39, subfamily a, polypeptide 1 |
| ENSMUST00000236902.1 | Gsto1 | glutathione S-transferase omega 1 |
| ENSMUST00000079859.6 | Klk1b27 | kallikrein 1-related peptidase b27 |
| ENSMUST00000029722.6 | Rps3a1 | ribosomal protein S3A1 |
| ENSMUST00000033683.7 | Rps4x | ribosomal protein S4, X-linked |
| ENSMUST00000022082.7 | Glrx | glutaredoxin |
| ENSMUST00000108716.7 | Aldh3a1 | aldehyde dehydrogenase family 3, subfamily A1 |
| ENSMUST00000022894.13 | Rnf103 | ring finger protein 103 |
| ENSMUST00000082409.1 | mt-Nd6 | mitochondrially encoded NADH dehydrogenase 6 |
| ENSMUST00000068581.8 | Gja1 | gap junction protein, alpha 1 |
| ENSMUST00000199958.1 | Gm43303 | predicted gene 43303 |
| ENSMUST00000176683.7 | Tex13a | testis expressed 13A |
| ENSMUST00000224432.1 | Dsc1 | desmocollin 1 |
| ENSMUST00000222978.1 | Gm45233 | predicted gene 45233 |
| ENSMUST00000099047.3 | Dnmt3aos | DNA methyltransferase 3A, opposite strand |
| ENSMUST00000082419.1 | mt-Nd6 | mitochondrially encoded NADH dehydrogenase 6 |
| ENSMUST00000042052.8 | Hectd1 | HECT domain containing 1 |
| ENSMUST00000049813.5 | Yod1 | YOD1 deubiquitinase |
| ENSMUST00000058714.9 | Cd24a | CD24a antigen |
| ENSMUST00000112384.9 | Rps24 | ribosomal protein S24 |
| ENSMUST00000114894.1 | Lyg1 | lysozyme G-like 1 |
| ENSMUST00000103172.3 | Dstn | destrin |
| ENSMUST00000037023.8 | Rps29 | ribosomal protein S29 |
| ENSMUST00000081650.14 | Mmel1 | membrane metallo-endopeptidase-like 1 |
| ENSMUST00000102483.4 | Rpl23a | ribosomal protein L23A |
| ENSMUST00000060738.8 | S100a1 | S100 calcium binding protein A1 |
| ENSMUST00000075453.8 | Rpl21 | ribosomal protein L21 |
| ENSMUST00000054599.7 | Sprr1a | small proline-rich protein 1A |
| ENSMUST00000079716.5 | Rpl17 | ribosomal protein L17 |
| ENSMUST00000020217.6 | Nudt4 | nudix (nucleoside diphosphate linked moiety X)-type motif 4 |
| ENSMUST00000008826.13 | Hcfc1 | host cell factor C1 |
| ENSMUST00000071641.4 | Gm15499 | predicted gene 15499 |
| ENSMUST00000180863.1 | Gm26807 | predicted gene, 26807 |
| ENSMUST00000092620.5 | Gm9767 | predicted gene 9767 |
| ENSMUST00000034511.6 | Trim29 | tripartite motif-containing 29 |
| ENSMUST00000057311.3 | Pkp3 | plakophilin 3 |
| ENSMUST00000032683.5 | Lypd5 | Ly6/Plaur domain containing 5 |
| ENSMUST00000026292.14 | Huwe1 | HECT, UBA and WWE domain containing 1 |
| ENSMUST00000026122.10 | P4hb | prolyl 4-hydroxylase, beta polypeptide |
| ENSMUST00000231768.1 | Fetub | fetuin beta |
| ENSMUST00000026050.7 | Gsto1 | glutathione S-transferase omega 1 |
| ENSMUST00000160758.7 | Mycbp2 | MYC binding protein 2 |
| ENSMUST00000182229.7 | Sik3 | SIK family kinase 3 |
| ENSMUST00000107619.2 | Klf4 | Kruppel-like factor 4 (gut) |
| ENSMUST00000156988.1 | Phlda1 | pleckstrin homology like domain, family A, member 1 |
| ENSMUST00000151376.2 | Gm10631 | predicted gene 10631 |
| ENSMUST00000027567.13 | Serpinb3a | serine (or cysteine) peptidase inhibitor, clade B (ovalbumin), member 3A |
| ENSMUST00000151120.8 | Ctsd | cathepsin D |
| ENSMUST00000028881.13 | Il1b | interleukin 1 beta |
| ENSMUST00000019649.3 | U2af2 | U2 small nuclear ribonucleoprotein auxiliary factor (U2AF) 2 |
| ENSMUST00000006496.14 | Rps9 | ribosomal protein S9 |
| ENSMUST00000098559.2 | Krtdap | keratinocyte differentiation associated protein |
| ENSMUST00000082219.5 | Fhl3 | four and a half LIM domains 3 |
| ENSMUST00000117507.8 | Ecm1 | extracellular matrix protein 1 |
| ENSMUST00000075573.6 | Gm10837 | predicted gene 10837 |
| ENSMUST00000185789.2 | Kcnq1ot1 | KCNQ1 overlapping transcript 1 |
| ENSMUST00000102814.4 | Rps6 | ribosomal protein S6 |
| ENSMUST00000034207.7 | Mt4 | metallothionein 4 |
| ENSMUST00000032330.15 | Emp1 | epithelial membrane protein 1 |
| ENSMUST00000073471.12 | Rpl26 | ribosomal protein L26 |
| ENSMUST00000081457.4 | Lgals7 | lectin, galactose binding, soluble 7 |
| ENSMUST00000026045.13 | Col17a1 | collagen, type XVII, alpha 1 |
| ENSMUST00000150819.2 | Ift140 | intraflagellar transport 140 |
| ENSMUST00000180308.2 | Flg | filaggrin |
| ENSMUST00000014058.10 | Klk10 | kallikrein related-peptidase 10 |
| ENSMUST00000110894.8 | Ubp1 | upstream binding protein 1 |
| ENSMUST00000094434.12 | Fth1 | ferritin heavy polypeptide 1 |
| ENSMUST00000019246.3 | Aldh3a1 | aldehyde dehydrogenase family 3, subfamily A1 |
| ENSMUST00000189941.1 | Gm10912 | predicted gene 10912 |
| ENSMUST00000039247.10 | Dsc2 | desmocollin 2 |
| ENSMUST00000225023.1 | Rps24 | ribosomal protein S24 |
| ENSMUST00000087268.6 | Abca12 | ATP-binding cassette, sub-family A (ABC1), member 12 |
| ENSMUST00000081840.5 | Rpl32 | ribosomal protein L32 |
| ENSMUST00000200401.1 | Aim1 | absent in melanoma 1 |
| ENSMUST00000092425.10 | Rpl19 | ribosomal protein L19 |
| ENSMUST00000221219.1 | Gm45233 | predicted gene 45233 |
| ENSMUST00000151118.1 | Rpl23 | ribosomal protein L23 |
| ENSMUST00000036424.2 | Alox12b | arachidonate 12-lipoxygenase, 12R type |
| ENSMUST00000020640.7 | Rack1 | receptor for activated C kinase 1 |
| ENSMUST00000018851.13 | Dync1h1 | dynein cytoplasmic 1 heavy chain 1 |
| ENSMUST00000101073.2 | Tmprss11a | transmembrane protease, serine 11a |
| ENSMUST00000167591.7 | Cnfn | cornifelin |
| ENSMUST00000012028.13 | Gltp | glycolipid transfer protein |
| ENSMUST00000107977.3 | Vsig10l | V-set and immunoglobulin domain containing 10 like |
| ENSMUST00000040404.7 | Ly6d | lymphocyte antigen 6 complex, locus D |
| ENSMUST00000102898.4 | Rpl7a | ribosomal protein L7A |
| ENSMUST00000055698.7 | Gjb2 | gap junction protein, beta 2 |
| ENSMUST00000020161.9 | Arg1 | arginase, liver |
| ENSMUST00000045897.14 | Pth1r | parathyroid hormone 1 receptor |
| ENSMUST00000106925.8 | Ngf | nerve growth factor |
| ENSMUST00000000608.7 | Gm2a | GM2 ganglioside activator protein |
| ENSMUST00000003521.9 | Rps11 | ribosomal protein S11 |
| ENSMUST00000099050.3 | Gm10115 | predicted gene 10115 |
| ENSMUST00000031617.12 | Rpl6 | ribosomal protein L6 |
| ENSMUST00000068408.13 | Rps15 | ribosomal protein S15 |
| ENSMUST00000029515.4 | S100a11 | S100 calcium binding protein A11 |
| ENSMUST00000102696.4 | Rps8 | ribosomal protein S8 |
| ENSMUST00000144826.1 | Noct | nocturnin |
| ENSMUST00000096570.3 | Gm94 | predicted gene 94 |
| ENSMUST00000070892.7 | Dsg3 | desmoglein 3 |
| ENSMUST00000161051.7 | Eif4g2 | eukaryotic translation initiation factor 4, gamma 2 |
| ENSMUST00000108707.2 | U2af2 | U2 small nuclear ribonucleoprotein auxiliary factor (U2AF) 2 |
| ENSMUST00000052248.7 | Eef1g | eukaryotic translation elongation factor 1 gamma |
| ENSMUST00000178079.7 | Rpl31 | ribosomal protein L31 |
| ENSMUST00000111882.8 | Ttn | titin |
| ENSMUST00000029794.10 | Them5 | thioesterase superfamily member 5 |
| ENSMUST00000004072.9 | Rpl8 | ribosomal protein L8 |
| ENSMUST00000081277.8 | Serpinb12 | serine (or cysteine) peptidase inhibitor, clade B (ovalbumin), member 12 |
| ENSMUST00000035077.7 | Ltf | lactotransferrin |
| ENSMUST00000057885.12 | Rpl9 | ribosomal protein L9 |
| ENSMUST00000082405.1 | mt-Nd6 | mitochondrially encoded NADH dehydrogenase 6 |
| ENSMUST00000174745.7 | Rps18 | ribosomal protein S18 |
| ENSMUST00000049251.5 | Cpa4 | carboxypeptidase A4 |
| ENSMUST00000056329.6 | Klk14 | kallikrein related-peptidase 14 |
| ENSMUST00000233892.1 | Vmn2r53 | vomeronasal 2, receptor 53 |
| ENSMUST00000165536.7 | Klf3 | Kruppel-like factor 3 (basic) |
| ENSMUST00000215393.1 | Gm45233 | predicted gene 45233 |
| ENSMUST00000178312.1 | Gm21876 | predicted gene, 21876 |
| ENSMUST00000000804.6 | Ddx3x | DEAD/H (Asp-Glu-Ala-Asp/His) box polypeptide 3, X-linked |
| ENSMUST00000090475.9 | Ehf | ets homologous factor |
| ENSMUST00000005218.14 | Cd44 | CD44 antigen |
| ENSMUST00000027629.9 | Tfcp2l1 | transcription factor CP2-like 1 |
| ENSMUST00000212129.1 | Rpl18a | ribosomal protein L18A |
| ENSMUST00000151346.7 | Macf1 | microtubule-actin crosslinking factor 1 |
| ENSMUST00000047028.8 | Lgalsl | lectin, galactoside binding-like |
| ENSMUST00000219916.1 | Gm45233 | predicted gene 45233 |
| ENSMUST00000023790.4 | Krt1 | keratin 1 |
| ENSMUST00000081017.2 | Defb4 | defensin beta 4 |
| ENSMUST00000080893.6 | Krt17 | keratin 17 |
| ENSMUST00000023720.7 | Krt84 | keratin 84 |
| ENSMUST00000173605.1 | Gm15564 | predicted gene 15564 |
| ENSMUST00000051272.7 | Wfdc12 | WAP four-disulfide core domain 12 |
| ENSMUST00000184437.1 | Mir6236 | microRNA 6236 |
| ENSMUST00000173478.1 | Ly6g6c | lymphocyte antigen 6 complex, locus G6C |
| ENSMUST00000052224.5 | Psapl1 | prosaposin-like 1 |
| ENSMUST00000095576.4 | Scel | sciellin |
| ENSMUST00000016771.12 | Myh9 | myosin, heavy polypeptide 9, non-muscle |
| ENSMUST00000086199.11 | Glul | glutamate-ammonia ligase (glutamine synthetase) |
| ENSMUST00000153941.7 | Slc38a6 | solute carrier family 38, member 6 |
| ENSMUST00000183302.5 | Dst | dystonin |
| ENSMUST00000034989.14 | Me1 | malic enzyme 1, NADP(+)-dependent, cytosolic |
| ENSMUST00000028239.7 | Gsn | gelsolin |
| ENSMUST00000115848.4 | Dsc3 | desmocollin 3 |
| ENSMUST00000094499.10 | Kif1c | kinesin family member 1C |
| ENSMUST00000034796.13 | Elovl4 | elongation of very long chain fatty acids (FEN1/Elo2, SUR4/Elo3, yeast)-like 4 |
| ENSMUST00000078835.2 | Klk1b1 | kallikrein 1-related peptidase b1 |
| ENSMUST00000221482.1 | Gm45233 | predicted gene 45233 |
| ENSMUST00000067512.7 | Smpd3 | sphingomyelin phosphodiesterase 3, neutral |
| ENSMUST00000066834.7 | Klk13 | kallikrein related-peptidase 13 |
| ENSMUST00000102844.3 | Rps27a | ribosomal protein S27A |
| ENSMUST00000025511.10 | Rps14 | ribosomal protein S14 |
| ENSMUST00000044326.4 | 2300002M23Rik | RIKEN cDNA 2300002M23 gene |
| ENSMUST00000029524.3 | Lce1d | late cornified envelope 1D |
| ENSMUST00000032934.11 | Aldoa | aldolase A, fructose-bisphosphate |
| ENSMUST00000076276.4 | Cnfn | cornifelin |
| ENSMUST00000224463.1 | Rps23 | ribosomal protein S23 |
| ENSMUST00000170715.7 | Rps2 | ribosomal protein S2 |
| ENSMUST00000058464.4 | Serpina9 | serine (or cysteine) peptidase inhibitor, clade A (alpha-1 antiproteinase, antitrypsin), member 9 |
| ENSMUST00000117557.7 | Il18r1 | interleukin 18 receptor 1 |
| ENSMUST00000107416.2 | Krt36 | keratin 36 |
| ENSMUST00000036493.7 | Atp1a1 | ATPase, Na+/K+ transporting, alpha 1 polypeptide |
| ENSMUST00000034834.15 | Pkm | pyruvate kinase, muscle |
| ENSMUST00000111529.7 | Ablim1 | actin-binding LIM protein 1 |
| ENSMUST00000016703.7 | H3f3b | H3 histone, family 3B |
| ENSMUST00000003100.9 | Cyp2f2 | cytochrome P450, family 2, subfamily f, polypeptide 2 |
| ENSMUST00000112271.9 | Ace2 | angiotensin I converting enzyme (peptidyl-dipeptidase A) 2 |
| ENSMUST00000167377.2 | Iqgap1 | IQ motif containing GTPase activating protein 1 |
| ENSMUST00000223888.1 | Gm45233 | predicted gene 45233 |
| ENSMUST00000080813.4 | Rps17 | ribosomal protein S17 |
| ENSMUST00000074142.6 | Lce1a1 | late cornified envelope 1A1 |
| ENSMUST00000103146.4 | Rpl23 | ribosomal protein L23 |
| ENSMUST00000109542.2 | Gm17130 | predicted gene 17130 |
| ENSMUST00000212820.1 | Ces2f | carboxylesterase 2F |
| ENSMUST00000032998.12 | Rps3 | ribosomal protein S3 |
| ENSMUST00000179881.1 | Gm11146 | predicted gene 11146 |
| ENSMUST00000049074.12 | Ptprf | protein tyrosine phosphatase, receptor type, F |
| ENSMUST00000090839.11 | Tlr4 | toll-like receptor 4 |
| ENSMUST00000218126.1 | Gm45233 | predicted gene 45233 |
| ENSMUST00000221317.1 | Daam1 | dishevelled associated activator of morphogenesis 1 |
| ENSMUST00000008991.7 | Sptbn2 | spectrin beta, non-erythrocytic 2 |
| ENSMUST00000090871.2 | Igf1os | insulin-like growth factor 1, opposite strand |
| ENSMUST00000059080.6 | Rps21 | ribosomal protein S21 |
| ENSMUST00000017348.2 | Gsdma | gasdermin A |
| ENSMUST00000004554.13 | Rps5 | ribosomal protein S5 |
| ENSMUST00000031017.10 | Fosl2 | fos-like antigen 2 |
| ENSMUST00000220994.1 | Gm45233 | predicted gene 45233 |
| ENSMUST00000090749.12 | Ppia | peptidylprolyl isomerase A |
| ENSMUST00000149566.1 | Rpl7 | ribosomal protein L7 |
| ENSMUST00000118875.7 | Gap43 | growth associated protein 43 |
| ENSMUST00000098911.9 | S100a16 | S100 calcium binding protein A16 |
| ENSMUST00000025561.7 | Anxa1 | annexin A1 |
| ENSMUST00000081619.9 | Sh3pxd2a | SH3 and PX domains 2A |
| ENSMUST00000187351.1 | Gm28967 | predicted gene 28967 |
| ENSMUST00000073868.8 | Naca | nascent polypeptide-associated complex alpha polypeptide |
| ENSMUST00000196248.1 | Ube2d3 | ubiquitin-conjugating enzyme E2D 3 |
| ENSMUST00000017435.10 | Taok1 | TAO kinase 1 |
| ENSMUST00000028135.14 | Fam129b | family with sequence similarity 129, member B |
| ENSMUST00000115231.3 | Rpl39 | ribosomal protein L39 |
| ENSMUST00000018632.10 | Myh4 | myosin, heavy polypeptide 4, skeletal muscle |
| ENSMUST00000219591.1 | Rps26 | ribosomal protein S26 |
| ENSMUST00000046234.4 | Lce3b | late cornified envelope 3B |
| ENSMUST00000029521.4 | Crct1 | cysteine-rich C-terminal 1 |
| ENSMUST00000071555.12 | Actc1 | actin, alpha, cardiac muscle 1 |
| ENSMUST00000086764.5 | Gm10197 | predicted gene 10197 |
| ENSMUST00000048957.10 | Rab19 | RAB19, member RAS oncogene family |
| ENSMUST00000080718.5 | Lypd3 | Ly6/Plaur domain containing 3 |
| ENSMUST00000069960.11 | S100a9 | S100 calcium binding protein A9 (calgranulin B) |
| ENSMUST00000107411.2 | Krt15 | keratin 15 |
| ENSMUST00000224130.1 | Gm45233 | predicted gene 45233 |
| ENSMUST00000041703.9 | Dmkn | dermokine |
| ENSMUST00000005077.6 | Hspb1 | heat shock protein 1 |
| ENSMUST00000029385.8 | Serp1 | stress-associated endoplasmic reticulum protein 1 |
| ENSMUST00000125184.7 | U2af2 | U2 small nuclear ribonucleoprotein auxiliary factor (U2AF) 2 |
| ENSMUST00000000312.11 | Cdh1 | cadherin 1 |
| ENSMUST00000079812.7 | Notch2 | notch 2 |
| ENSMUST00000053298.7 | Rpl27a | ribosomal protein L27A |
| ENSMUST00000055375.5 | Lce3c | late cornified envelope 3C |
| ENSMUST00000038377.8 | Bsg | basigin |
| ENSMUST00000173499.1 | Malat1 | metastasis associated lung adenocarcinoma transcript 1 (non-coding RNA) |
| ENSMUST00000026459.5 | Atp5b | ATP synthase, H+ transporting mitochondrial F1 complex, beta subunit |
| ENSMUST00000055327.7 | Aqp3 | aquaporin 3 |
| ENSMUST00000038760.9 | Lad1 | ladinin |
| ENSMUST00000021062.11 | Ddx5 | DEAD (Asp-Glu-Ala-Asp) box polypeptide 5 |
| ENSMUST00000045870.4 | Rnf225 | ring finger protein 225 |
| ENSMUST00000179982.1 | Gm10837 | predicted gene 10837 |
| ENSMUST00000178695.1 | Flg | filaggrin |
| ENSMUST00000034756.14 | Anxa2 | annexin A2 |
| ENSMUST00000222959.1 | Gm45233 | predicted gene 45233 |
| ENSMUST00000106644.8 | Nrd1 | nardilysin, N-arginine dibasic convertase, NRD convertase 1 |
| ENSMUST00000034766.13 | Rora | RAR-related orphan receptor alpha |
| ENSMUST00000064234.6 | Ezr | ezrin |
| ENSMUST00000106588.7 | Rps15a | ribosomal protein S15A |
| ENSMUST00000108682.8 | Gas7 | growth arrest specific 7 |
| ENSMUST00000172910.2 | Crybg3 | beta-gamma crystallin domain containing 3 |
| ENSMUST00000000756.5 | Rpl13 | ribosomal protein L13 |
| ENSMUST00000090866.1 | Lce1i | late cornified envelope 1I |
| ENSMUST00000142643.1 | Rpl30 | ribosomal protein L30 |
| ENSMUST00000079085.10 | Rpl34 | ribosomal protein L34 |
| ENSMUST00000005279.7 | Klf5 | Kruppel-like factor 5 |
| ENSMUST00000206383.1 | Snhg1 | small nucleolar RNA host gene 1 |
| ENSMUST00000133426.7 | Ddx5 | DEAD (Asp-Glu-Ala-Asp) box polypeptide 5 |
| ENSMUST00000057974.3 | Nccrp1 | non-specific cytotoxic cell receptor protein 1 homolog (zebrafish) |
| ENSMUST00000023226.12 | Plec | plectin |
| ENSMUST00000031175.11 | Tmprss11d | transmembrane protease, serine 11d |
| ENSMUST00000090863.4 | Rwdd2a | RWD domain containing 2A |
| ENSMUST00000025955.7 | Eif3a | eukaryotic translation initiation factor 3, subunit A |
| ENSMUST00000085688.10 | Dmkn | dermokine |
| ENSMUST00000167598.5 | S100a14 | S100 calcium binding protein A14 |
| ENSMUST00000216990.1 | Rpl14 | ribosomal protein L14 |
| ENSMUST00000081940.10 | U2af2 | U2 small nuclear ribonucleoprotein auxiliary factor (U2AF) 2 |
| ENSMUST00000160719.7 | Mfap3l | microfibrillar-associated protein 3-like |
| ENSMUST00000210792.1 | Rpl13a | ribosomal protein L13A |
| ENSMUST00000045303.9 | Spns2 | spinster homolog 2 |
| ENSMUST00000192639.5 | Prdx6 | peroxiredoxin 6 |
| ENSMUST00000233427.1 | Rpl10a | ribosomal protein L10A |
| ENSMUST00000131070.1 | Ide | insulin degrading enzyme |
| ENSMUST00000216591.1 | Ide | insulin degrading enzyme |
| ENSMUST00000078357.4 | Emp2 | epithelial membrane protein 2 |
| ENSMUST00000022519.14 | Anxa8 | annexin A8 |
| ENSMUST00000043584.4 | Tubb4b | tubulin, beta 4B class IVB |
| ENSMUST00000128725.1 | Rps3 | ribosomal protein S3 |
| ENSMUST00000090558.9 | Celsr2 | cadherin, EGF LAG seven-pass G-type receptor 2 |
| ENSMUST00000067318.5 | Lce3a | late cornified envelope 3A |
| ENSMUST00000188966.1 | Gm28265 | predicted gene 28265 |
| ENSMUST00000029049.6 | Chmp4c | charged multivesicular body protein 4C |
| ENSMUST00000179272.1 | Dnmt3aos | DNA methyltransferase 3A, opposite strand |
| ENSMUST00000086701.12 | Serpinb5 | serine (or cysteine) peptidase inhibitor, clade B, member 5 |
| ENSMUST00000205406.1 | Unclassified | Unclassified |
| ENSMUST00000102672.4 | Nfe2l2 | nuclear factor, erythroid derived 2, like 2 |
| ENSMUST00000191436.6 | Ly6e | lymphocyte antigen 6 complex, locus E |
| ENSMUST00000226420.1 | Rmrp | RNA component of mitochondrial RNAase P |
| ENSMUST00000061242.7 | Arf1 | ADP-ribosylation factor 1 |
| ENSMUST00000029527.5 | Pycrl | pyrroline-5-carboxylate reductase-like |
| ENSMUST00000233162.1 | Xdh | xanthine dehydrogenase |
| ENSMUST00000020608.2 | Ppp2ca | protein phosphatase 2 (formerly 2A), catalytic subunit, alpha isoform |
| ENSMUST00000023741.15 | Kmt2d | lysine (K)-specific methyltransferase 2D |
| ENSMUST00000162963.7 | Wdr26 | WD repeat domain 26 |
| ENSMUST00000047055.3 | Lce1c | late cornified envelope 1C |
| ENSMUST00000163893.7 | Eps8l1 | EPS8-like 1 |
| ENSMUST00000000137.7 | Actr2 | ARP2 actin-related protein 2 |
| ENSMUST00000034740.14 | Nedd4 | neural precursor cell expressed, developmentally down-regulated 4 |
| ENSMUST00000021001.9 | Rab10 | RAB10, member RAS oncogene family |
| ENSMUST00000135469.7 | Zak | sterile alpha motif and leucine zipper containing kinase AZK |
| ENSMUST00000030164.7 | Vcp | valosin containing protein |
| ENSMUST00000127305.1 | Epn3 | epsin 3 |
| ENSMUST00000165123.7 | Csnk1a1 | casein kinase 1, alpha 1 |
| ENSMUST00000001184.9 | Mxd1 | MAX dimerization protein 1 |
| ENSMUST00000155805.1 | Rps16 | ribosomal protein S16 |
| ENSMUST00000051521.4 | Lipf | lipase, gastric |
| ENSMUST00000194061.1 | Fmo2 | flavin containing monooxygenase 2 |
| ENSMUST00000048209.15 | Ldha | lactate dehydrogenase A |
| ENSMUST00000026577.12 | Eps8l2 | EPS8-like 2 |
| ENSMUST00000103009.4 | Rps20 | ribosomal protein S20 |
| ENSMUST00000111846.8 | Ttn | titin |
| ENSMUST00000034903.6 | Gsta4 | glutathione S-transferase, alpha 4 |
| ENSMUST00000027565.4 | Casp9 | caspase 9 |
| ENSMUST00000075214.8 | Dsc2 | desmocollin 2 |
| ENSMUST00000029653.6 | Egf | epidermal growth factor |
| ENSMUST00000098886.4 | Lce3e | late cornified envelope 3E |
| ENSMUST00000103186.10 | Cltc | clathrin, heavy polypeptide (Hc) |
| ENSMUST00000037947.14 | Mcl1 | myeloid cell leukemia sequence 1 |
| ENSMUST00000220310.1 | Gm45233 | predicted gene 45233 |
| ENSMUST00000028288.4 | Notch1 | notch 1 |
| ENSMUST00000087435.6 | Bmpr2 | bone morphogenetic protein receptor, type II (serine/threonine kinase) |
| ENSMUST00000033310.8 | Mki67 | antigen identified by monoclonal antibody Ki 67 |
| ENSMUST00000021773.12 | Serpinb9 | serine (or cysteine) peptidase inhibitor, clade B, member 9 |
| ENSMUST00000047153.3 | Lce1f | late cornified envelope 1F |
| ENSMUST00000035840.5 | Zdhhc5 | zinc finger, DHHC domain containing 5 |
| ENSMUST00000219227.1 | Plekhn1 | pleckstrin homology domain containing, family N member 1 |
| ENSMUST00000088621.10 | Srrm2 | serine/arginine repetitive matrix 2 |
| ENSMUST00000033824.7 | Lamp1 | lysosomal-associated membrane protein 1 |
| ENSMUST00000029530.5 | Lce1a2 | late cornified envelope 1A2 |
| ENSMUST00000133266.1 | Ssfa2 | sperm specific antigen 2 |
| ENSMUST00000028386.11 | Nckap1 | NCK-associated protein 1 |
| ENSMUST00000014221.12 | Chp1 | calcineurin-like EF hand protein 1 |
| ENSMUST00000032492.8 | Cd9 | CD9 antigen |
| ENSMUST00000001989.8 | Uba1 | ubiquitin-like modifier activating enzyme 1 |
| ENSMUST00000100171.2 | Hspa5 | heat shock protein 5 |
| ENSMUST00000226722.1 | Itm2b | integral membrane protein 2B |
| ENSMUST00000112172.3 | Tmsb4x | thymosin, beta 4, X chromosome |
| ENSMUST00000050758.4 | Flg | filaggrin |
| ENSMUST00000100799.8 | Rpl15 | ribosomal protein L15 |
| ENSMUST00000017975.6 | Rab5a | RAB5A, member RAS oncogene family |
| ENSMUST00000059980.10 | Rpl37a | ribosomal protein L37a |
| ENSMUST00000076623.7 | Arap2 | ArfGAP with RhoGAP domain, ankyrin repeat and PH domain 2 |
| ENSMUST00000027727.14 | Adipor1 | adiponectin receptor 1 |
| ENSMUST00000040440.6 | Calm2 | calmodulin 2 |
| ENSMUST00000032955.6 | Klk7 | kallikrein related-peptidase 7 (chymotryptic, stratum corneum) |
| ENSMUST00000037182.13 | Hook3 | hook microtubule tethering protein 3 |
| ENSMUST00000236217.1 | Tbx5 | T-box 5 |
| ENSMUST00000156793.1 | Pabpc1 | poly(A) binding protein, cytoplasmic 1 |
| ENSMUST00000029531.3 | Lce1b | late cornified envelope 1B |
| ENSMUST00000233317.1 | Vmn2r76 | vomeronasal 2, receptor 76 |
| ENSMUST00000041956.13 | Spag9 | sperm associated antigen 9 |
| ENSMUST00000052965.7 | Nipbl | Nipped-B homolog (Drosophila) |
| ENSMUST00000049149.14 | Tlr4 | toll-like receptor 4 |
| ENSMUST00000044500.5 | Gm4117 | predicted gene 4117 |
| ENSMUST00000218107.1 | Rps12 | ribosomal protein S12 |
| ENSMUST00000072503.12 | Rpl18 | ribosomal protein L18 |
| ENSMUST00000055745.4 | Nlrp10 | NLR family, pyrin domain containing 10 |
| ENSMUST00000017851.3 | Serinc3 | serine incorporator 3 |
| ENSMUST00000106291.9 | Ube2d3 | ubiquitin-conjugating enzyme E2D 3 |
| ENSMUST00000068853.12 | Fat2 | FAT atypical cadherin 2 |
| ENSMUST00000169681.2 | Eif4b | eukaryotic translation initiation factor 4B |
| ENSMUST00000080926.12 | Eng | endoglin |
| ENSMUST00000152441.1 | Rpl35 | ribosomal protein L35 |
| ENSMUST00000212806.1 | Mt2 | metallothionein 2 |
| ENSMUST00000019076.9 | Map2k3 | mitogen-activated protein kinase kinase 3 |
| ENSMUST00000191428.6 | Fat1 | FAT atypical cadherin 1 |
| ENSMUST00000040023.15 | Iffo2 | intermediate filament family orphan 2 |
| ENSMUST00000088646.11 | Wnk1 | WNK lysine deficient protein kinase 1 |
| ENSMUST00000003912.6 | Calr | calreticulin |
| ENSMUST00000026989.14 | 4833439L19Rik | RIKEN cDNA 4833439L19 gene |
| ENSMUST00000034597.7 | Tmprss13 | transmembrane protease, serine 13 |
| ENSMUST00000060945.11 | Aff4 | AF4/FMR2 family, member 4 |
| ENSMUST00000051477.12 | Cdc42 | cell division cycle 42 |
| ENSMUST00000110082.10 | Calm1 | calmodulin 1 |
| ENSMUST00000020692.6 | Btg2 | B cell translocation gene 2, anti-proliferative |
| ENSMUST00000000939.14 | Hip1r | huntingtin interacting protein 1 related |
| ENSMUST00000121046.1 | Trim50 | tripartite motif-containing 50 |
| ENSMUST00000020637.8 | Canx | calnexin |
| ENSMUST00000136312.1 | U2af2 | U2 small nuclear ribonucleoprotein auxiliary factor (U2AF) 2 |
| ENSMUST00000213235.1 | Taf1d | TATA-box binding protein associated factor, RNA polymerase I, D |
| ENSMUST00000207750.1 | Akap13 | A kinase (PRKA) anchor protein 13 |
| ENSMUST00000092298.5 | Zfp750 | zinc finger protein 750 |
| ENSMUST00000022587.9 | Tsc22d1 | TSC22 domain family, member 1 |
| ENSMUST00000030127.12 | Tmem38b | transmembrane protein 38B |
| ENSMUST00000227322.1 | Scel | sciellin |
| ENSMUST00000170122.3 | Psmd8 | proteasome (prosome, macropain) 26S subunit, non-ATPase, 8 |
| ENSMUST00000022691.13 | Hr | hairless |
| ENSMUST00000007959.13 | Rhoa | ras homolog family member A |
| ENSMUST00000066337.12 | Alas2 | aminolevulinic acid synthase 2, erythroid |
| ENSMUST00000042345.7 | Ctnna1 | catenin (cadherin associated protein), alpha 1 |
| ENSMUST00000165532.2 | Rpl14 | ribosomal protein L14 |
| ENSMUST00000025649.9 | Ddb1 | damage specific DNA binding protein 1 |
| ENSMUST00000025356.3 | Mal2 | mal, T cell differentiation protein 2 |
| ENSMUST00000179343.2 | Purb | purine rich element binding protein B |
| ENSMUST00000146468.3 | Lgals3 | lectin, galactose binding, soluble 3 |
| ENSMUST00000101454.8 | Flna | filamin, alpha |
| ENSMUST00000005017.14 | Hdgf | hepatoma-derived growth factor |

**Appendix Table 3** Enriched gene ontologies (Biological Process) in the mouse host oral metatranscriptome with a Enrichr-calculated combined score greater than 12, in health (blue) and disease (red)

| **Enriched GO Biological Process** | **Enriched Condition** | **P-value** | **Combined Score** | **Genes** |
| --- | --- | --- | --- | --- |
| dendrite extension (GO:0097484) | Health | 0.0010 | 173.7876 | CDKL3;AUTS2;TMEM108 |
| regulation of t-circle formation (GO:1904429) | Health | 0.0010 | 173.7876 | RTEL1;SLX4;ERCC1 |
| negative regulation of protein kinase activity by regulation of protein phosphorylation (GO:0044387) | Health | 0.0017 | 120.2870 | NPM1;ADARB1;CORO1C |
| retrograde axonal transport (GO:0008090) | Health | 0.0002 | 95.4422 | DST;FBXW11;TMEM108;KIF1C;SOD1 |
| regulation of myeloid leukocyte differentiation (GO:0002761) | Health | 0.0027 | 89.5435 | UBASH3B;MTOR;FOXP1 |
| TORC1 signaling (GO:0038202) | Health | 0.0027 | 89.5435 | CLEC16A;MLST8;MTOR |
| regulation of cellular response to growth factor stimulus (GO:0090287) | Health | 0.0003 | 84.1491 | TFAP2B;FST;CASK;SLIT2;FSTL1 |
| auditory receptor cell morphogenesis (GO:0002093) | Health | 0.0135 | 72.3061 | PDZD7;SOD1 |
| auditory receptor cell stereocilium organization (GO:0060088) | Health | 0.0135 | 72.3061 | PDZD7;SOD1 |
| regulation of cardioblast differentiation (GO:0051890) | Health | 0.0135 | 72.3061 | TGFB2;TBX5 |
| cardiac muscle cell proliferation (GO:0060038) | Health | 0.0135 | 72.3061 | NDRG4;TGFB2 |
| striated muscle cell proliferation (GO:0014855) | Health | 0.0135 | 72.3061 | NDRG4;TGFB2 |
| Toll signaling pathway (GO:0008063) | Health | 0.0135 | 72.3061 | PELI3;PELI2 |
| negative regulation of autophagosome maturation (GO:1901097) | Health | 0.0135 | 72.3061 | PHF23;CLEC16A |
| negative regulation of macrophage cytokine production (GO:0010936) | Health | 0.0135 | 72.3061 | CUEDC2;TGFB2 |
| positive regulation of cardioblast differentiation (GO:0051891) | Health | 0.0135 | 72.3061 | TGFB2;TBX5 |
| bone cell development (GO:0098751) | Health | 0.0039 | 69.8619 | THPO;SH2B3;FOXP1 |
| protein prenylation (GO:0018342) | Health | 0.0055 | 56.3311 | FNTB;PGGT1B;RABGGTA |
| regulation of organ growth (GO:0046620) | Health | 0.0055 | 56.3311 | LATS2;WWC1;SOD1 |
| forelimb morphogenesis (GO:0035136) | Health | 0.0055 | 56.3311 | TFAP2B;TBX5;TBX3 |
| adherens junction maintenance (GO:0034334) | Health | 0.0198 | 49.4384 | FERMT2;PLEKHA7 |
| regulation of glucokinase activity (GO:0033131) | Health | 0.0198 | 49.4384 | MIDN;BAD |
| choline catabolic process (GO:0042426) | Health | 0.0198 | 49.4384 | SARDH;ALDH7A1 |
| regulation of membrane tubulation (GO:1903525) | Health | 0.0198 | 49.4384 | ASAP1;DNM2 |
| regulation of microtubule motor activity (GO:2000574) | Health | 0.0198 | 49.4384 | CFAP73;FBXW11 |
| equilibrioception (GO:0050957) | Health | 0.0198 | 49.4384 | USH1G;CLRN1 |
| inner ear auditory receptor cell differentiation (GO:0042491) | Health | 0.0198 | 49.4384 | MYO6;PDZD7 |
| megakaryocyte development (GO:0035855) | Health | 0.0198 | 49.4384 | THPO;SH2B3 |
| negative regulation of chemokine-mediated signaling pathway (GO:0070100) | Health | 0.0198 | 49.4384 | SLIT2;SH2B3 |
| negative regulation of lamellipodium organization (GO:1902744) | Health | 0.0198 | 49.4384 | PLXNB3;SLIT2 |
| protein geranylgeranylation (GO:0018344) | Health | 0.0198 | 49.4384 | PGGT1B;RABGGTA |
| axonal transport (GO:0098930) | Health | 0.0004 | 48.3486 | BLOC1S6;DST;BLOC1S1;FBXW11;  TMEM108;KIF1B;SOD1 |
| purine ribonucleotide transport (GO:0015868) | Health | 0.0034 | 44.1908 | ABCC4;SLC35B2;CALHM1;SLC19A1 |
| cellular modified amino acid catabolic process (GO:0042219) | Health | 0.0034 | 44.1908 | ABHD16A;SARDH;HOGA1;PRODH |
| adenine nucleotide transport (GO:0051503) | Health | 0.0042 | 39.4378 | ABCC4;SLC35B2;CALHM1;SLC19A1 |
| regulation of sequestering of calcium ion (GO:0051282) | Health | 0.0095 | 39.1912 | RYR2;UBASH3B;CASQ2 |
| bleb assembly (GO:0032060) | Health | 0.0270 | 36.4130 | ANO6;MYLK |
| cardiac pacemaker cell development (GO:0060926) | Health | 0.0270 | 36.4130 | TBX5;TBX3 |
| cardiolipin biosynthetic process (GO:0032049) | Health | 0.0270 | 36.4130 | TAMM41;CRLS1 |
| cellular response to caffeine (GO:0071313) | Health | 0.0270 | 36.4130 | RYR2;CASQ2 |
| collagen-activated tyrosine kinase receptor signaling pathway (GO:0038063) | Health | 0.0270 | 36.4130 | COL1A1;UBASH3B |
| cyclic nucleotide transport (GO:0070729) | Health | 0.0270 | 36.4130 | ABCC4;SLC19A1 |
| response to caffeine (GO:0031000) | Health | 0.0270 | 36.4130 | RYR2;CASQ2 |
| histone H3-K14 acetylation (GO:0044154) | Health | 0.0270 | 36.4130 | ING5;BRPF3 |
| negative regulation of DNA repair (GO:0045738) | Health | 0.0270 | 36.4130 | UBR5;TRIP12 |
| negative regulation of protein K63-linked ubiquitination (GO:1900045) | Health | 0.0270 | 36.4130 | UBR5;TRIP12 |
| positive regulation of oxidative stress-induced cell death (GO:1903209) | Health | 0.0270 | 36.4130 | TLR4;SOD1 |
| regulation of telomere maintenance (GO:0032204) | Health | 0.0027 | 35.5515 | RTEL1;ERCC1;TINF2;SIRT6;HDAC8 |
| pore complex assembly (GO:0046931) | Health | 0.0052 | 35.4189 | BAD;TPR;ANO6;PLEKHA7 |
| extracellular transport (GO:0006858) | Health | 0.0120 | 33.4999 | SPAG17;CCDC103;WWP2 |
| negative regulation of small GTPase mediated signal transduction (GO:0051058) | Health | 0.0076 | 29.0270 | TGFB2;MFN2;SPRY1;SLIT2 |
| regulation of response to endoplasmic reticulum stress (GO:1905897) | Health | 0.0148 | 28.9891 | NR1H2;LPCAT3;NUPR1 |
| ribonucleoside monophosphate biosynthetic process (GO:0009156) | Health | 0.0148 | 28.9891 | ADSL;ADK;GART |
| mismatch repair (GO:0006298) | Health | 0.0045 | 28.4893 | PCNA;LIG1;ERCC1;RPA1;PMS1 |
| regulation of chemokine-mediated signaling pathway (GO:0070099) | Health | 0.0351 | 28.1387 | SLIT2;SH2B3 |
| regulation of protein ADP-ribosylation (GO:0010835) | Health | 0.0351 | 28.1387 | KAT2B;TINF2 |
| regulation of wound healing, spreading of epidermal cells (GO:1903689) | Health | 0.0351 | 28.1387 | FERMT2;MTOR |
| integrin activation (GO:0033622) | Health | 0.0351 | 28.1387 | FN1;FERMT2 |
| self proteolysis (GO:0097264) | Health | 0.0351 | 28.1387 | CAPN7;CAPN1 |
| negative regulation of mRNA polyadenylation (GO:1900364) | Health | 0.0351 | 28.1387 | RNF20;NELFE |
| plasma membrane phospholipid scrambling (GO:0017121) | Health | 0.0351 | 28.1387 | ANO6;ANO7 |
| progesterone receptor signaling pathway (GO:0050847) | Health | 0.0351 | 28.1387 | UBR5;UBE3A |
| TRIF-dependent toll-like receptor signaling pathway (GO:0035666) | Health | 0.0052 | 26.5855 | TBK1;TRAF3;UBE2D3;IKBKG;TLR4 |
| limb morphogenesis (GO:0035108) | Health | 0.0090 | 26.4563 | TFAP2B;TGFB2;TBX5;TBX3 |
| MyD88-independent toll-like receptor signaling pathway (GO:0002756) | Health | 0.0060 | 24.8593 | TBK1;TRAF3;UBE2D3;IKBKG;TLR4 |
| transcription initiation from RNA polymerase I promoter (GO:0006361) | Health | 0.0069 | 23.2889 | TAF1B;TAF1C;POLR1C;UBTF;TAF1 |
| anoikis (GO:0043276) | Health | 0.0440 | 22.4900 | IKBKG;MTOR |
| regulation of complement-dependent cytotoxicity (GO:1903659) | Health | 0.0440 | 22.4900 | TGFB2;CR1L |
| collagen-activated signaling pathway (GO:0038065) | Health | 0.0440 | 22.4900 | COL1A1;UBASH3B |
| embryonic forelimb morphogenesis (GO:0035115) | Health | 0.0440 | 22.4900 | TBX5;TBX3 |
| epithelial cell apoptotic process (GO:1904019) | Health | 0.0440 | 22.4900 | RYR2;HIPK1 |
| maintenance of apical/basal cell polarity (GO:0035090) | Health | 0.0440 | 22.4900 | CRB2;ANK1 |
| maintenance of epithelial cell apical/basal polarity (GO:0045199) | Health | 0.0440 | 22.4900 | CRB2;ANK1 |
| negative regulation of mRNA 3'-end processing (GO:0031441) | Health | 0.0440 | 22.4900 | RNF20;NELFE |
| negative regulation of protein-containing complex disassembly (GO:0043242) | Health | 0.0440 | 22.4900 | PHF23;CLEC16A |
| phosphatidylglycerol biosynthetic process (GO:0006655) | Health | 0.0440 | 22.4900 | TAMM41;CRLS1 |
| positive regulation of phagocytosis, engulfment (GO:0060100) | Health | 0.0440 | 22.4900 | ABCA7;ANO6 |
| positive regulation of epithelial to mesenchymal transition (GO:0010718) | Health | 0.0050 | 22.3586 | CRB2;COL1A1;TIAM1;TGFB2;MTOR;  FERMT2 |
| regulation of interleukin-1 production (GO:0032652) | Health | 0.0215 | 22.3487 | MEFV;TLR4;FOXP1 |
| negative regulation of telomere maintenance (GO:0032205) | Health | 0.0215 | 22.3487 | RTEL1;SLX4;ERCC1 |
| regulation of Ras protein signal transduction (GO:0046578) | Health | 0.0016 | 21.4470 | STARD13;TGFB2;SHC1;MFN2;STK19;  ARHGAP17;DENND3;SPRY1;RAF1;  RASGRP4 |
| transcription by RNA polymerase I (GO:0006360) | Health | 0.0090 | 20.5444 | TAF1B;TAF1C;POLR1C;UBTF;TAF1 |
| negative regulation of endocytosis (GO:0045806) | Health | 0.0142 | 20.4736 | LRSAM1;NR1H2;PACSIN3;ABCA7 |
| regulation of cell killing (GO:0031341) | Health | 0.0254 | 19.8544 | TGFB2;CR1L;DVL2 |
| regulation of ion transmembrane transport (GO:0034765) | Health | 0.0101 | 19.3410 | CALHM1;AKAP9;CASQ2;ANO6;WWP2 |
| hippo signaling (GO:0035329) | Health | 0.0162 | 18.9130 | TEAD4;LATS2;WWC1;DVL2 |
| negative regulation of macroautophagy (GO:0016242) | Health | 0.0162 | 18.9130 | PHF23;CLEC16A;NUPR1;MTOR |
| organelle fission (GO:0048285) | Health | 0.0162 | 18.9130 | MTFR1L;MFF;CORO1C;DNM2 |
| protein O-linked glycosylation via serine (GO:0018242) | Health | 0.0536 | 18.4305 | POGLUT1;GALNT1 |
| cellular biogenic amine catabolic process (GO:0042402) | Health | 0.0536 | 18.4305 | SARDH;ALDH7A1 |
| cellular response to purine-containing compound (GO:0071415) | Health | 0.0536 | 18.4305 | RYR2;CASQ2 |
| inner ear receptor cell stereocilium organization (GO:0060122) | Health | 0.0536 | 18.4305 | PDZD7;SOD1 |
| telomeric loop disassembly (GO:0090657) | Health | 0.0536 | 18.4305 | RTEL1;SLX4 |
| myelin maintenance (GO:0043217) | Health | 0.0536 | 18.4305 | EPB41L3;SOD1 |
| negative regulation of cholesterol storage (GO:0010887) | Health | 0.0536 | 18.4305 | NR1H2;PPARD |
| peptidyl-lysine acetylation (GO:0018394) | Health | 0.0536 | 18.4305 | KAT2B;BLOC1S1 |
| positive regulation of establishment of protein localization to telomere (GO:1904851) | Health | 0.0536 | 18.4305 | DKC1;CCT8 |
| positive regulation of keratinocyte migration (GO:0051549) | Health | 0.0536 | 18.4305 | MTOR;MAP4K4 |
| positive regulation of transcription of nucleolar large rRNA by RNA polymerase I (GO:1901838) | Health | 0.0536 | 18.4305 | IPPK;MTOR |
| regulation of oxidative stress-induced intrinsic apoptotic signaling pathway (GO:1902175) | Health | 0.0296 | 17.7522 | BAG5;UBQLN1;SOD1 |
| establishment of vesicle localization (GO:0051650) | Health | 0.0296 | 17.7522 | CCDC186;IKBKG;DNM2 |
| regulation of telomere maintenance via telomere lengthening (GO:1904356) | Health | 0.0296 | 17.7522 | RTEL1;SLX4;TINF2 |
| mitochondrial fission (GO:0000266) | Health | 0.0296 | 17.7522 | MTFR1L;MFF;DNM2 |
| nucleotide biosynthetic process (GO:0009165) | Health | 0.0296 | 17.7522 | ADSL;NMNAT1;GART |
| anterograde axonal transport (GO:0008089) | Health | 0.0128 | 17.2131 | BLOC1S6;BLOC1S1;KIF1C;KIF1B;SOD1 |
| response to ketone (GO:1901654) | Health | 0.0210 | 16.2639 | TGFB2;UBE3A;CSN1S1;SLIT2 |
| negative regulation of response to stimulus (GO:0048585) | Health | 0.0085 | 15.6539 | COL3A1;UBASH3B;NR1H2;LPCAT3;  CASK;SLIT2;SH2B3 |
| regulation of macroautophagy (GO:0016241) | Health | 0.0038 | 15.4328 | TBK1;CLEC16A;MLST8;UBQLN1;  CAPN1;IKBKG;ATP6V0D1;LAMTOR3;  ATP6V1C1;MTOR;EXOC1 |
| protein localization to phagophore assembly site (GO:0034497) | Health | 0.0639 | 15.3986 | MFN2;WDR45 |
| cell-cell junction maintenance (GO:0045217) | Health | 0.0639 | 15.3986 | CSF1R;FERMT2 |
| regulation of establishment of protein localization to telomere (GO:0070203) | Health | 0.0639 | 15.3986 | DKC1;CCT8 |
| regulation of protein localization to Cajal body (GO:1904869) | Health | 0.0639 | 15.3986 | DKC1;CCT8 |
| formation of extrachromosomal circular DNA (GO:0001325) | Health | 0.0639 | 15.3986 | SLX4;ERCC1 |
| t-circle formation (GO:0090656) | Health | 0.0639 | 15.3986 | SLX4;ERCC1 |
| telomere maintenance via telomere trimming (GO:0090737) | Health | 0.0639 | 15.3986 | SLX4;ERCC1 |
| negative regulation of centrosome duplication (GO:0010826) | Health | 0.0639 | 15.3986 | KAT2B;NPM1 |
| nucleus localization (GO:0051647) | Health | 0.0639 | 15.3986 | FBXW11;MTOR |
| positive regulation of protein localization to Cajal body (GO:1904871) | Health | 0.0639 | 15.3986 | DKC1;CCT8 |
| telomere maintenance via telomerase (GO:0007004) | Health | 0.0236 | 15.1338 | RTEL1;DKC1;TINF2;RPA1 |
| branched-chain amino acid catabolic process (GO:0009083) | Health | 0.0391 | 14.4238 | AUH;BCKDHB;HSD17B10 |
| branched-chain amino acid metabolic process (GO:0009081) | Health | 0.0391 | 14.4238 | AUH;BCKDHB;HSD17B10 |
| cardiac epithelial to mesenchymal transition (GO:0060317) | Health | 0.0391 | 14.4238 | TGFB2;SPRY1;TBX3 |
| myeloid cell development (GO:0061515) | Health | 0.0391 | 14.4238 | THPO;SH2B3;FOXP1 |
| regulation of epithelial to mesenchymal transition (GO:0010717) | Health | 0.0084 | 14.2280 | CRB2;COL1A1;TIAM1;TGFB2;SPRY1;  TBX5;MTOR;FERMT2 |
| alpha-amino acid catabolic process (GO:1901606) | Health | 0.0264 | 14.1111 | SARDH;HOGA1;PRODH;HSD17B10 |
| RNA-dependent DNA biosynthetic process (GO:0006278) | Health | 0.0264 | 14.1111 | RTEL1;DKC1;TINF2;RPA1 |
| termination of RNA polymerase I transcription (GO:0006363) | Health | 0.0264 | 14.1111 | TAF1B;TAF1C;POLR1C;UBTF |
| regulation of intrinsic apoptotic signaling pathway (GO:2001242) | Health | 0.0140 | 14.0741 | SGMS1;BAD;RTKN2;PLAUR;UBQLN1;  NUPR1 |
| plasma membrane organization (GO:0007009) | Health | 0.0109 | 14.0335 | BIN3;EPB41L3;OTOF;PACSIN3;ANO6;ABCA7;FER1L6 |
| heart morphogenesis (GO:0003007) | Health | 0.0153 | 13.4891 | RYR2;TGFB2;ACTC1;CHD7;EPHB4;  TBX3 |
| regulation of developmental growth (GO:0048638) | Health | 0.0294 | 13.1826 | MBD5;LATS2;DNM2;SOD1 |
| regulation of transmembrane receptor protein serine/threonine kinase signaling pathway (GO:0090092) | Health | 0.0294 | 13.1826 | TFAP2B;LATS2;FST;FSTL1 |
| peptide cross-linking (GO:0018149) | Health | 0.0294 | 13.1826 | FLG;COL3A1;FN1;ABCA7 |
| regulation of double-strand break repair (GO:2000779) | Health | 0.0213 | 13.1311 | RTEL1;UBR5;SPIRE2;SIRT6;TRIP12 |
| purinergic nucleotide receptor signaling pathway (GO:0035590) | Health | 0.0444 | 13.0915 | ANO6;SUGT1;MEFV |
| response to progesterone (GO:0032570) | Health | 0.0444 | 13.0915 | TGFB2;UBE3A;CSN1S1 |
| nucleotide-excision repair, DNA incision, 3'-to lesion (GO:0006295) | Health | 0.0444 | 13.0915 | ERCC1;RPA1;ERCC5 |
| nucleotide-excision repair, preincision complex stabilization (GO:0006293) | Health | 0.0444 | 13.0915 | ERCC1;RPA1;ERCC5 |
| purine nucleoside monophosphate biosynthetic process (GO:0009127) | Health | 0.0748 | 13.0651 | ADSL;GART |
| purine nucleotide biosynthetic process (GO:0006164) | Health | 0.0748 | 13.0651 | ADSL;GART |
| carnitine metabolic process (GO:0009437) | Health | 0.0748 | 13.0651 | CPT1A;ALDH9A1 |
| regulation of keratinocyte migration (GO:0051547) | Health | 0.0748 | 13.0651 | MTOR;MAP4K4 |
| megakaryocyte differentiation (GO:0030219) | Health | 0.0748 | 13.0651 | THPO;SH2B3 |
| mesoderm morphogenesis (GO:0048332) | Health | 0.0748 | 13.0651 | CRB2;TBX3 |
| negative regulation of vascular permeability (GO:0043116) | Health | 0.0748 | 13.0651 | SLIT2;FERMT2 |
| positive regulation of cardiocyte differentiation (GO:1905209) | Health | 0.0748 | 13.0651 | TGFB2;TBX5 |
| positive regulation of protein localization to chromosome, telomeric region (GO:1904816) | Health | 0.0748 | 13.0651 | DKC1;CCT8 |
| positive regulation of telomere maintenance (GO:0032206) | Health | 0.0327 | 12.3369 | RTEL1;SLX4;ERCC1;SIRT6 |
| actin filament organization (GO:0007015) | Health | 0.0057 | 12.2883 | SVIL;TPM3;ARHGAP17;RHOF;  SHROOM1;CORO1C;MYO1B;BIN3;ACTC1;DPYSL3;MYO6;SPIRE2;CLRN1 |
| DNA-templated transcription, initiation (GO:0006352) | Health | 0.0053 | 12.1226 | TEAD4;NR1H2;TBX5;ESR2;KAT2B;  NR6A1;TAF1B;POLR2B;TAF1C;  POLR1C;UBTF;TAF4B;PPARD;TAF1 |
| CD40 signaling pathway (GO:0023035) | Disease | 0.0017 | 61.5113 | CD86;ITGB1;RNF31;TNIP2;PHB2 |
| apoptotic chromosome condensation (GO:0030263) | Disease | 0.0127 | 50.4663 | TOP2A;DFFB;ACIN1 |
| negative regulation of protein polyubiquitination (GO:1902915) | Disease | 0.0127 | 50.4663 | GPS2;PPIA;TRIM44 |
| negative regulation of T cell migration (GO:2000405) | Disease | 0.0127 | 50.4663 | CD200R1;APOD;CD200 |
| positive regulation of MHC class I biosynthetic process (GO:0045345) | Disease | 0.0127 | 50.4663 | CIITA;HSPH1;NLRC5 |
| positive regulation of toll-like receptor 9 signaling pathway (GO:0034165) | Disease | 0.0127 | 50.4663 | RSAD2;PTPN22;RTN4 |
| regulation of nucleotide-binding oligomerization domain containing signaling pathway (GO:0070424) | Disease | 0.0127 | 50.4663 | SLC15A2;XIAP;BIRC2 |
| SRP-dependent cotranslational protein targeting to membrane, translocation (GO:0006616) | Disease | 0.0127 | 50.4663 | SEC61A2;SEC61A1;ZFAND2B |
| succinyl-CoA metabolic process (GO:0006104) | Disease | 0.0127 | 50.4663 | SUCLA2;DLST;SUCLG2 |
| cellular response to UV-B (GO:0071493) | Disease | 0.0083 | 36.9024 | CDKN1A;STK11;HYAL3;CRIP1 |
| regulation of pattern recognition receptor signaling pathway (GO:0062207) | Disease | 0.0083 | 36.9024 | SLC15A2;XIAP;CD36;BIRC2 |
| coenzyme A biosynthetic process (GO:0015937) | Disease | 0.0051 | 33.9282 | PANK4;PANK2;DCAKD;PPCDC;ACAT1 |
| purine nucleoside bisphosphate biosynthetic process (GO:0034033) | Disease | 0.0051 | 33.9282 | PANK4;PANK2;DCAKD;PPCDC;ACAT1 |
| ribonucleoside bisphosphate biosynthetic process (GO:0034030) | Disease | 0.0051 | 33.9282 | PANK4;PANK2;DCAKD;PPCDC;ACAT1 |
| COPII-coated vesicle cargo loading (GO:0090110) | Disease | 0.0030 | 33.5280 | SEC13;SEC23A;TBC1D20;SEC24D;  SEC23B;SEC24C |
| sulfur compound transport (GO:0072348) | Disease | 0.0007 | 30.9596 | SLC25A26;SLC6A6;SLC27A1;SLC44A4;RACGAP1;SLC25A30;LRRC8D;  SLC26A11;SLC25A14;SLC26A6 |
| aminophospholipid transport (GO:0015917) | Disease | 0.0232 | 28.9870 | ATP8A1;TMEM30B;ATP11B |
| B cell chemotaxis (GO:0035754) | Disease | 0.0232 | 28.9870 | CH25H;HSD3B7;PIK3CD |
| cholesterol import (GO:0070508) | Disease | 0.0232 | 28.9870 | SCARB1;STARD5;CD36 |
| imitative learning (GO:0098596) | Disease | 0.0232 | 28.9870 | NRXN1;HTT;NRXN2 |
| IMP biosynthetic process (GO:0006188) | Disease | 0.0232 | 28.9870 | AMPD2;AMPD3;PFAS |
| intracellular pH elevation (GO:0051454) | Disease | 0.0232 | 28.9870 | CLN3;CFTR;SLC26A6 |
| melanosome assembly (GO:1903232) | Disease | 0.0232 | 28.9870 | TRAPPC6A;ABCB6;HPS4 |
| positive regulation of endothelial cell development (GO:1901552) | Disease | 0.0232 | 28.9870 | S1PR2;F11R;ADD1 |
| positive regulation of establishment of endothelial barrier (GO:1903142) | Disease | 0.0232 | 28.9870 | S1PR2;F11R;ADD1 |
| postsynaptic density assembly (GO:0097107) | Disease | 0.0232 | 28.9870 | NRXN1;ZDHHC12;NRXN2 |
| protein K27-linked ubiquitination (GO:0044314) | Disease | 0.0232 | 28.9870 | UBE2D4;UBE2S;RNF6 |
| regulation of endoplasmic reticulum tubular network organization (GO:1903371) | Disease | 0.0232 | 28.9870 | ATL3;TMEM33;RAB3GAP1 |
| regulation of extracellular exosome assembly (GO:1903551) | Disease | 0.0232 | 28.9870 | PDCD6IP;SDC1;STAM |
| regulation of hematopoietic stem cell proliferation (GO:1902033) | Disease | 0.0232 | 28.9870 | THPO;KAT7;PDCD2 |
| response to manganese ion (GO:0010042) | Disease | 0.0232 | 28.9870 | LRRK2;ADAM9;D2HGDH |
| snoRNA localization (GO:0048254) | Disease | 0.0232 | 28.9870 | NOP58;ZNHIT3;PRPF31 |
| sterol import (GO:0035376) | Disease | 0.0232 | 28.9870 | SCARB1;STARD5;CD36 |
| vocal learning (GO:0042297) | Disease | 0.0232 | 28.9870 | NRXN1;HTT;NRXN2 |
| regulation of RNA splicing (GO:0043484) | Disease | 0.0001 | 27.4348 | MBNL1;MBNL2;KHDRBS3;CELF1;  YTHDC1;RRP1B;HNRNPLL;PQBP1;  PTBP3;CLK2;CLK1;PTBP1;HNRNPK;  SON;ESRP2;CWC22;SRSF3;HNRNPH3;GRSF1;SF1;ZPR1 |
| endoplasmic reticulum membrane organization (GO:0090158) | Disease | 0.0079 | 26.6542 | ATL1;LPCAT3;STX18;MOSPD3;RTN4 |
| protein localization to synapse (GO:0035418) | Disease | 0.0079 | 26.6542 | MPP4;DLG4;NRXN1;ZDHHC12;NRXN2 |
| cellular response to muramyl dipeptide (GO:0071225) | Disease | 0.0136 | 26.4796 | TRIM41;PTPN22;ARHGEF2;VIM |
| negative regulation of bone resorption (GO:0045779) | Disease | 0.0136 | 26.4796 | P2RX7;UBASH3B;GPR137;TNFAIP3 |
| protein K6-linked ubiquitination (GO:0085020) | Disease | 0.0136 | 26.4796 | UBE2D4;UBE2S;RNF6;BRCA1 |
| actin filament capping (GO:0051693) | Disease | 0.0038 | 25.0901 | SVIL;CAPZB;CAPZA2;CAPG;ADD3;  ADD1;ADD2 |
| barbed-end actin filament capping (GO:0051016) | Disease | 0.0038 | 25.0901 | SVIL;CAPZB;CAPZA2;CAPG;ADD3;  ADD1;ADD2 |
| negative regulation of GTPase activity (GO:0034260) | Disease | 0.0030 | 23.8889 | ARFGEF1;BCAS3;FICD;RASA4;LRRK2;  RRP1B;F11R;GMIP |
| response to ionizing radiation (GO:0010212) | Disease | 0.0004 | 21.8607 | IKBIP;BRSK1;CDKN1A;BRAT1;RNF8;  AEN;BRCA1;USP28;TANK;CLK2;TICRR;RNF168;RAD51AP1;STK11;KAT5;  UIMC1;ATM;TOPBP1;RAD9A |
| response to UV-B (GO:0010224) | Disease | 0.0116 | 21.4550 | CDKN1A;STK11;HYAL3;CRIP1;RELA |
| acetyl-CoA biosynthetic process (GO:0006085) | Disease | 0.0207 | 19.9244 | PDHA1;DIP2A;PDHB;ACAT1 |
| lipoprotein transport (GO:0042953) | Disease | 0.0207 | 19.9244 | VMP1;PRKCB;CD36;ZDHHC17 |
| membrane lipid catabolic process (GO:0046466) | Disease | 0.0207 | 19.9244 | SMPD2;SGPL1;FUCA1;NAGA |
| negative regulation of leukocyte migration (GO:0002686) | Disease | 0.0207 | 19.9244 | CD200R1;EMILIN1;SLIT2;CD200 |
| peptidyl-amino acid modification (GO:0018193) | Disease | 0.0207 | 19.9244 | FKBP10;METAP1D;TTLL4;METAP2 |
| regulation of macrophage migration (GO:1905521) | Disease | 0.0207 | 19.9244 | CD200R1;MTUS1;EMILIN1;CD200 |
| retinoic acid receptor signaling pathway (GO:0048384) | Disease | 0.0207 | 19.9244 | RXRB;TBX1;SNW1;RARB |
| interstrand cross-link repair (GO:0036297) | Disease | 0.0010 | 19.8897 | DCLRE1C;WDR48;FANCM;ERCC6L2;  FANCL;MCM9;RNF8;RPA1;FAAP24;  FANCC;FANCG;RNF168;RAD51AP1;  RAD51AP2;SLX4 |
| cardiolipin biosynthetic process (GO:0032049) | Disease | 0.0372 | 19.0157 | PGS1;SLC27A1;PTPMT1 |
| dense core granule cytoskeletal transport (GO:0099519) | Disease | 0.0372 | 19.0157 | KIF5B;KIF1C;TANC2 |
| histone H3-K14 acetylation (GO:0044154) | Disease | 0.0372 | 19.0157 | BRPF3;PHF14;KAT7 |
| iron coordination entity transport (GO:1901678) | Disease | 0.0372 | 19.0157 | SLC48A1;ABCB6;LTF |
| mitochondrion-endoplasmic reticulum membrane tethering (GO:1990456) | Disease | 0.0372 | 19.0157 | VMP1;AHCYL1;PACS2 |
| positive regulation of histone H4 acetylation (GO:0090240) | Disease | 0.0372 | 19.0157 | AUTS2;KAT7;BRCA1 |
| positive regulation of membrane depolarization (GO:1904181) | Disease | 0.0372 | 19.0157 | MLLT11;KDR;TRPM4 |
| posttranslational protein targeting to membrane, translocation (GO:0031204) | Disease | 0.0372 | 19.0157 | SEC61A2;SEC61A1;SEC61G |
| protein catabolic process in the vacuole (GO:0007039) | Disease | 0.0372 | 19.0157 | CLN3;VPS13A;TCIRG1 |
| regulation of MHC class I biosynthetic process (GO:0045343) | Disease | 0.0372 | 19.0157 | CIITA;HSPH1;NLRC5 |
| vascular wound healing (GO:0061042) | Disease | 0.0372 | 19.0157 | MCAM;KDR;NDNF |
| protein quality control for misfolded or incompletely synthesized proteins (GO:0006515) | Disease | 0.0054 | 18.9641 | ATXN3;UBE2W;TOR1A;ZER1;KLHL15;  YME1L1;UGGT1;RNF185 |
| negative regulation of innate immune response (GO:0045824) | Disease | 0.0028 | 18.4375 | CNOT7;STAT2;DHX58;NLRC5;TYRO3;  TNFAIP3;FAM3A;SERPING1;NMI;  SAMHD1;USP18 |
| cardiolipin metabolic process (GO:0032048) | Disease | 0.0164 | 17.5968 | HADHB;PGS1;HADHA;SLC27A1;  PTPMT1 |
| coenzyme A metabolic process (GO:0015936) | Disease | 0.0164 | 17.5968 | PANK4;PANK2;DCAKD;PPCDC;ACAT1 |
| N-terminal protein amino acid modification (GO:0031365) | Disease | 0.0164 | 17.5968 | METAP1D;AANAT;NAA10;NAA35;  METAP2 |
| positive regulation of transmembrane transport (GO:0034764) | Disease | 0.0164 | 17.5968 | P2RX7;C2CD5;AZIN2;AZIN1;SLC26A6 |
| regulation of IRE1-mediated unfolded protein response (GO:1903894) | Disease | 0.0164 | 17.5968 | FICD;DDRGK1;TMEM33;BAX;BFAR |
| sulfate transport (GO:0008272) | Disease | 0.0164 | 17.5968 | RACGAP1;SLC25A30;SLC26A11;  SLC25A14;SLC26A6 |
| mRNA splice site selection (GO:0006376) | Disease | 0.0052 | 17.3917 | SF3A1;CELF1;SFSWAP;YTHDC1;CELF2;CELF4;LUC7L;LUC7L2;SF1 |
| positive regulation of DNA repair (GO:0045739) | Disease | 0.0024 | 17.3439 | PRKCG;DHX9;EYA2;RNF8;XRCC1;  BRCA1;RNF168;FAM168A;UIMC1;  RPS3;SPIRE2;ERCC8;ERCC6 |
| regulation of DNA repair (GO:0006282) | Disease | 0.0040 | 15.5380 | RNF168;DHX9;EYA2;UIMC1;RNF8;  RPS3;UBE2V2;ERCC8;KAT7;BRCA1;  SIRT7;ERCC6 |
| cell differentiation in spinal cord (GO:0021515) | Disease | 0.0296 | 15.4993 | SOX13;MDGA2;SOX6;MDGA1 |
| lipoprotein localization (GO:0044872) | Disease | 0.0296 | 15.4993 | VMP1;PRKCB;CD36;ZDHHC17 |
| negative regulation of bone remodeling (GO:0046851) | Disease | 0.0296 | 15.4993 | P2RX7;UBASH3B;GPR137;TNFAIP3 |
| positive regulation of DNA-dependent DNA replication (GO:2000105) | Disease | 0.0296 | 15.4993 | STOML2;ATRX;CDC7;E2F7 |
| regulation of toll-like receptor 9 signaling pathway (GO:0034163) | Disease | 0.0296 | 15.4993 | RSAD2;PTPN22;RAB7B;RTN4 |
| UDP-N-acetylglucosamine biosynthetic process (GO:0006048) | Disease | 0.0296 | 15.4993 | GNPDA2;PGM3;GNPNAT1;RENBP |
| regulation of RNA metabolic process (GO:0051252) | Disease | 0.0023 | 15.2841 | MBNL1;MBNL2;CELF1;RRP1B;HNRNPLL;RC3H2;PQBP1;PTBP3;CLK2;CLK1;  PTBP1;SON;ESRP2;IGF2BP3;HNRNPH3;GRSF1 |
| regulation of mRNA processing (GO:0050684) | Disease | 0.0060 | 15.1723 | AHCYL1;KHDRBS3;HNRNPK;SON;  DHX9;YTHDC1;CWC22;SRSF3;  CDK11B;SF1 |
| positive regulation of histone H3-K4 methylation (GO:0051571) | Disease | 0.0223 | 14.6492 | SNW1;AUTS2;CTR9;WDR61;BRCA1 |
| positive regulation of response to cytokine stimulus (GO:0060760) | Disease | 0.0223 | 14.6492 | DDX58;DHX9;AXL;AGPAT1;TRIM44 |
| carbohydrate derivative catabolic process (GO:1901136) | Disease | 0.0166 | 14.5886 | MANBA;FUCA1;NAGA;BPGM;  SAMHD1;FBXO44 |
| negative regulation of RNA metabolic process (GO:0051253) | Disease | 0.0166 | 14.5886 | NCOR2;ZC3H10;PTBP1;NCOR1;ESR1;  PTBP3 |
| posttranscriptional regulation of gene expression (GO:0010608) | Disease | 0.0048 | 14.5517 | RBM3;ZC3H10;PER1;ATXN2;LARP1;  ZC3H7A;YTHDC1;PCIF1;NOLC1;TSC1;MATR3;PUM3 |
| regulation of plasma membrane bounded cell projection organization (GO:0120035) | Disease | 0.0113 | 13.8241 | BRSK1;NTNG2;CCDC88A;BRSK2;  HOMER1;FRMD7;FCGR2B;CAMK2G |
| regulation of telomere maintenance via telomerase (GO:0032210) | Disease | 0.0050 | 13.6182 | ACD;PINX1;TINF2;NAT10;SMG5;  PKIB;TCP1;FBXO4;PRKCQ;ATM;  HNRNPC;MAP2K7;CCT4 |
| establishment of protein localization to peroxisome (GO:0072663) | Disease | 0.0545 | 13.4445 | PEX19;PEX1;PEX12 |
| natural killer cell differentiation (GO:0001779) | Disease | 0.0545 | 13.4445 | AXL;PIK3CD;KAT7 |
| negative regulation of macrophage migration (GO:1905522) | Disease | 0.0545 | 13.4445 | CD200R1;EMILIN1;CD200 |
| positive regulation of cell migration by vascular endothelial growth factor signaling pathway (GO:0038089) | Disease | 0.0545 | 13.4445 | MYO1C;KDR;PIK3CD |
| positive regulation of fibroblast migration (GO:0010763) | Disease | 0.0545 | 13.4445 | ITGB1;PRKCE;ARHGEF7 |
| regulation of chemokine-mediated signaling pathway (GO:0070099) | Disease | 0.0545 | 13.4445 | SLIT2;HIF1A;SH2B3 |
| regulation of dendritic spine maintenance (GO:1902950) | Disease | 0.0545 | 13.4445 | PRNP;HOMER1;FCGR2B |
| regulation of Golgi to plasma membrane protein transport (GO:0042996) | Disease | 0.0545 | 13.4445 | CLN3;CSK;ATP2C1 |
| regulation of protein ADP-ribosylation (GO:0010835) | Disease | 0.0545 | 13.4445 | TINF2;XRCC1;PUM3 |
| regulation of protein K63-linked ubiquitination (GO:1900044) | Disease | 0.0545 | 13.4445 | GPS2;PTPN22;BIRC2 |
| regulation of toll-like receptor 3 signaling pathway (GO:0034139) | Disease | 0.0545 | 13.4445 | FLOT1;TNFAIP3;PTPN22 |
| ribosome disassembly (GO:0032790) | Disease | 0.0545 | 13.4445 | GFM2;MTIF2;MTIF3 |
| positive regulation of protein targeting to membrane (GO:0090314) | Disease | 0.0156 | 13.2170 | PRNP;TCAF2;C2CD5;ITGAM;MYO1C;  CIB1;MFF |
| protein K48-linked deubiquitination (GO:0071108) | Disease | 0.0156 | 13.2170 | ATXN3;USP37;USP27X;USP5;USP34;  TNFAIP3;BAP1 |
| regulation of mRNA splicing, via spliceosome (GO:0048024) | Disease | 0.0027 | 13.0645 | SF3B4;ZC3H10;MBNL1;MBNL2;  RBFOX2;KHDRBS3;CELF1;SFSWAP;  YTHDC1;CELF2;CELF4;RBM3;PTBP1;  HNRNPK;SNW1;SON;CWC22;SRSF3;  SRSF7;SF1 |
| regulation of interleukin-2 production (GO:0032663) | Disease | 0.0069 | 12.7993 | CD86;PRNP;STOML2;KAT5;IRF4;  TRAF6;RPS3;TNFAIP3;TRIM27;HDAC7;CCR2;HAVCR2 |
| cellular response to misfolded protein (GO:0071218) | Disease | 0.0214 | 12.6975 | ATXN3;UBE2W;TOR1A;KLHL15;  RNF185;UGGT1 |
| amino sugar biosynthetic process (GO:0046349) | Disease | 0.0405 | 12.3581 | GNPDA2;PGM3;GNPNAT1;RENBP |
| negative regulation of gene silencing by miRNA (GO:0060965) | Disease | 0.0405 | 12.3581 | NCOR2;ZC3H10;NCOR1;ESR1 |
| negative regulation of production of miRNAs involved in gene silencing by miRNA (GO:1903799) | Disease | 0.0405 | 12.3581 | NCOR2;ZC3H10;NCOR1;ESR1 |
| nuclear body organization (GO:0030575) | Disease | 0.0405 | 12.3581 | DYRK3;USPL1;ETS1;ZPR1 |
| negative regulation of type I interferon-mediated signaling pathway (GO:0060339) | Disease | 0.0295 | 12.3441 | CNOT7;STAT2;NLRC5;SAMHD1;USP18 |
| organelle disassembly (GO:1903008) | Disease | 0.0125 | 12.1678 | GOLGA2;DYRK3;GFM2;DDRGK1;KIF5B;GBF1;MTIF2;ULK1;MTIF3 |
| protein localization to chromosome (GO:0034502) | Disease | 0.0125 | 12.1678 | ACD;PARP3;MCM9;PINX1;ATRX;TINF2;RPA1;CENPA;ESR1 |

**Appendix Table 4** Gene ontologies related to immune functions and pathways in health (blue) and disease (red) among the Enrichr generated GOs (based on a combined score threshold of 12) and their associated upregulated mouse host genes.

| **Immune linked Gene Ontology** | **Corresponding genes** |
| --- | --- |
| regulation of t-circle formation (GO:1904429) | RTEL1;SLX4;ERCC1 |
| regulation of myeloid leukocyte differentiation (GO:0002761) | UBASH3B;MTOR;FOXP1 |
| Toll signaling pathway (GO:0008063) | PELI3;PELI2 |
| negative regulation of autophagosome maturation (GO:1901097) | PHF23;CLEC16A |
| negative regulation of macrophage cytokine production (GO:0010936) | CUEDC2;TGFB2 |
| negative regulation of chemokine-mediated signaling pathway (GO:0070100) | SLIT2;SH2B3 |
| regulation of chemokine-mediated signaling pathway (GO:0070099) | SLIT2;SH2B3 |
| TRIF-dependent toll-like receptor signaling pathway (GO:0035666) | TBK1;TRAF3;UBE2D3;IKBKG;TLR4 |
| MyD88-independent toll-like receptor signaling pathway (GO:0002756) | TBK1;TRAF3;UBE2D3;IKBKG;TLR4 |
| regulation of complement-dependent cytotoxicity (GO:1903659) | TGFB2;CR1L |
| regulation of interleukin-1 production (GO:0032652) | MEFV;TLR4;FOXP1 |
| negative regulation of macroautophagy (GO:0016242) | PHF23;CLEC16A;NUPR1;MTOR |
| CD40 signaling pathway (GO:0023035) | CD86;ITGB1;RNF31;TNIP2;PHB2 |
| negative regulation of T cell migration (GO:2000405) | CD200R1;APOD;CD200 |
| positive regulation of MHC class I biosynthetic process (GO:0045345) | CIITA;HSPH1;NLRC5 |
| positive regulation of toll-like receptor 9 signaling pathway (GO:0034165) | RSAD2;PTPN22;RTN4 |
| regulation of pattern recognition receptor signaling pathway (GO:0062207) | SLC15A2;XIAP;CD36;BIRC2 |
| B cell chemotaxis (GO:0035754) | CH25H;HSD3B7;PIK3CD |
| negative regulation of leukocyte migration (GO:0002686) | CD200R1;EMILIN1;SLIT2;CD200 |
| regulation of macrophage migration (GO:1905521) | CD200R1;MTUS1;EMILIN1;CD200 |
| regulation of MHC class I biosynthetic process (GO:0045343) | CIITA;HSPH1;NLRC5 |
| negative regulation of innate immune response (GO:0045824) | CNOT7;STAT2;DHX58;NLRC5;TYRO3;TNFAIP3;  FAM3A;SERPING1;NMI;SAMHD1;USP18 |
| regulation of toll-like receptor 9 signaling pathway (GO:0034163) | RSAD2;PTPN22;RAB7B;RTN4 |
| positive regulation of response to cytokine stimulus (GO:0060760) | DDX58;DHX9;AXL;AGPAT1;TRIM44 |
| natural killer cell differentiation (GO:0001779) | AXL;PIK3CD;KAT7 |
| negative regulation of macrophage migration (GO:1905522) | CD200R1;EMILIN1;CD200 |
| positive regulation of fibroblast migration (GO:0010763) | ITGB1;PRKCE;ARHGEF7 |
| regulation of chemokine-mediated signaling pathway (GO:0070099) | SLIT2;HIF1A;SH2B3 |
| regulation of toll-like receptor 3 signaling pathway (GO:0034139) | FLOT1;TNFAIP3;PTPN22 |
| regulation of interleukin-2 production (GO:0032663) | CD86;PRNP;STOML2;KAT5;IRF4;TRAF6;RPS3;  TNFAIP3;TRIM27;HDAC7;CCR2;HAVCR2 |

**Appendix Table 5** KEGG IDs, their annotated descriptions and associated pathways of the 29 differentially expressed bacterial genes in health (blue) and disease (red) with FC>2. The genes have been listed in decreasing order of fold change and the asterisks refer to the genes that were present only in the indicated condition and completely absent otherwise.

| **KEGG** | **KEGG DESCRIPTION** | **PATHWAYS DESCRIPTION** |
| --- | --- | --- |
| K03189 * | ureG; urease accessory protein | Protein processing |
| K07011 * | wbbL; rhamnosyltransferase | Function unknown |
| K00052 * | leuB, IMDH; 3-isopropylmalate dehydrogenase | C5-Branched dibasic acid metabolism\|Valine, leucine and isoleucine biosynthesis |
| K02007 * | cbiM; cobalt/nickel transport system permease protein | ABC transporters \|Transporters |
| K03092 * | rpoN; RNA polymerase sigma-54 factor | Two-component system\|Biofilm formation - Vibrio cholerae \|Transcription machinery |
| K01408 * | IDE, ide; insulysin | Alzheimer disease \|Peptidases |
| K16153 * | glycogen phosphorylase/synthase | Starch and sucrose metabolism \|Glycosyltransferases |
| K05523 | hchA; D-lactate dehydratase / protein deglycase | Pyruvate metabolism \|Peptidases |
| K02171 | blaI; BlaI family transcriptional regulator, penicillinase repressor | beta-Lactam resistance \|Transcription factors \|Antimicrobial resistance genes |
| K01186 | NEU1; sialidase-1 | Sphingolipid metabolism\|Other glycan degradation \|Lysosome \|Bacterial toxins |
| K03495 | gidA, mnmG, MTO1; tRNA uridine 5-carboxymethylaminomethyl modification enzyme | Transfer RNA biogenesis \|Chromosome and associated proteins |
| K04744 | lptD, imp, ostA; LPS-assembly protein | Transporters |
| K00091 * | dihydroflavonol-4-reductase | Enzymes with EC numbers |
| K00163 * | aceE; pyruvate dehydrogenase E1 component | Glycolysis / Gluconeogenesis \|Citrate cycle (TCA cycle) \|Pyruvate metabolism |
| K00508 * | linoleoyl-CoA desaturase | Linoleic acid metabolism \|Lipid biosynthesis proteins |
| K01653 * | ilvH, ilvN; acetolactate synthase I/III small subunit | Butanoate metabolism \|C5-Branched dibasic acid metabolism \|Valine, leucine and isoleucine biosynthesis \|Pantothenate and CoA biosynthesis |
| K02083 * | allC; allantoate deiminase | Purine metabolism \|Peptidases |
| K02405 * | fliA; RNA polymerase sigma factor for flagellar operon FliA | Two-component system \|Biofilm formation - Vibrio cholerae \|Biofilm formation - Pseudomonas aeruginosa \|Biofilm formation - Escherichia coli \|Flagellar assembly \|Transcription machinery \|Bacterial motility proteins |
| K03118 * | tatC; sec-independent protein translocase protein TatC | Protein export \|Bacterial secretion system \|Secretion system |
| K03187 * | ureE; urease accessory protein | Protein processing |
| K15629 * | Fatty Acid peroxygenase | Enzymes with EC numbers |
| K10118 | msmF; raffinose/stachyose/melibiose transport system permease protein | ABC transporters \|Transporters |
| K00005 | gldA; glycerol dehydrogenase | Propanoate metabolism \|Glycerolipid metabolism |
| K08156 | araJ; MFS transporter, DHA1 family, arabinose polymer utilization protein | Transporters |
| K00260 | gudB, rocG; glutamate dehydrogenase | Nitrogen metabolism \|Alanine, aspartate and glutamate metabolism \|Arginine biosynthesis \|Taurine and hypotaurine metabolism |
| K09022 | ridA, tdcF, RIDA; 2-iminobutanoate/2-iminopropanoate deaminase | Enzymes with EC numbers |
| K00240 | sdhB, frdB; succinate dehydrogenase / fumarate reductase, iron-sulfur subunit | Citrate cycle (TCA cycle) \|Butanoate metabolism \|Oxidative phosphorylation \|Carbon fixation pathways in prokaryotes |
| K03781 | katE, CAT, catB, srpA; catalase | Glyoxylate and dicarboxylate metabolism \|Tryptophan metabolism \|MAPK signalling pathway - plant \|MAPK signalling pathway - yeast \|FoxO signalling pathway \|Peroxisome \|Longevity regulating pathway \|Longevity regulating pathway - worm\| Longevity regulating pathway - multiple species \|Amyotrophic lateral sclerosis (ALS) |
| K03148 | thiF; sulfur carrier protein ThiS adenylyltransferase | Thiamine metabolism \|Sulfur relay system |

**Appendix Table 6** Enriched gene ontologies from the top three sub-categories of biological processes – cellular process, metabolic process and biological regulation – in the mouse host oral metatranscriptome, in health (blue) and disease (red). The top twenty candidates in each sub-category based on their Enrichr-calculated combined score have been indicated along with their corresponding differentially expressed genes.

| **Enriched GO** | **Biological Process** | **Enriched Condition** | **Genes** | **Combined Score** | **P-value** |
| --- | --- | --- | --- | --- | --- |
| negative regulation of protein kinase activity by regulation of protein phosphorylation (GO:0044387) | Cellular Process | Health | NPM1;ADARB1;CORO1C | 366.97 | 0.00018 |
| retrograde axonal transport (GO:0008090) | Cellular Process | Health | DST;FBXW11;TMEM108;KIF1C;SOD1 | 311.35 | 0.00001 |
| regulation of myeloid leukocyte differentiation (GO:0002761) | Cellular Process | Health | UBASH3B;MTOR;FOXP1 | 278.04 | 0.00028 |
| TORC1 signaling (GO:0038202) | Cellular Process | Health | CLEC16A;MLST8;MTOR | 278.04 | 0.00028 |
| regulation of cellular response to growth factor stimulus (GO:0090287) | Cellular Process | Health | TFAP2B;FST;CASK;SLIT2;FSTL1 | 277.47 | 0.00001 |
| regulation of cardioblast differentiation (GO:0051890) | Cellular Process | Health | TGFB2;TBX5 | 219.29 | 0.00295 |
| cardiac muscle cell proliferation (GO:0060038) | Cellular Process | Health | NDRG4;TGFB2 | 219.29 | 0.00295 |
| striated muscle cell proliferation (GO:0014855) | Cellular Process | Health | NDRG4;TGFB2 | 219.29 | 0.00295 |
| Toll signaling pathway (GO:0008063) | Cellular Process | Health | PELI3;PELI2 | 219.29 | 0.00295 |
| negative regulation of autophagosome maturation (GO:1901097) | Cellular Process | Health | PHF23;CLEC16A | 219.29 | 0.00295 |
| positive regulation of cardioblast differentiation (GO:0051891) | Cellular Process | Health | TGFB2;TBX5 | 219.29 | 0.00295 |
| regulation of organ growth (GO:0046620) | Cellular Process | Health | LATS2;WWC1;SOD1 | 180.72 | 0.00058 |
| forelimb morphogenesis (GO:0035136) | Cellular Process | Health | TFAP2B;TBX5;TBX3 | 180.72 | 0.00058 |
| axonal transport (GO:0098930) | Cellular Process | Health | BLOC1S6;DST;BLOC1S1;FBXW11;TMEM108;  KIF1B;SOD1 | 177.82 | 0.00000 |
| regulation of microtubule motor activity (GO:2000574) | Cellular Process | Health | CFAP73;FBXW11 | 153.34 | 0.00437 |
| dendrite extension (GO:0097484) | Cellular Process | Health | CDKL3;TMEM108 | 153.34 | 0.00437 |
| regulation of t-circle formation (GO:1904429) | Cellular Process | Health | SLX4;ERCC1 | 153.34 | 0.00437 |
| negative regulation of chemokine-mediated signaling pathway (GO:0070100) | Cellular Process | Health | SLIT2;SH2B3 | 153.34 | 0.00437 |
| negative regulation of lamellipodium organization (GO:1902744) | Cellular Process | Health | PLXNB3;SLIT2 | 153.34 | 0.00437 |
| protein geranylgeranylation (GO:0018344) | Cellular Process | Health | PGGT1B;RABGGTA | 153.34 | 0.00437 |
| CD40 signaling pathway (GO:0023035) | Cellular Process | Disease | CD86;ITGB1;RNF31;TNIP2;PHB2 | 220.10 | 0.00005 |
| SRP-dependent cotranslational protein targeting to membrane, translocation (GO:0006616) | Cellular Process | Disease | SEC61A2;SEC61A1;ZFAND2B | 173.25 | 0.00143 |
| COPII-coated vesicle cargo loading (GO:0090110) | Cellular Process | Disease | SEC13;SEC23A;TBC1D20;SEC24D;SEC23B;  SEC24C | 131.46 | 0.00005 |
| coenzyme A biosynthetic process (GO:0015937) | Cellular Process | Disease | PANK4;PANK2;DCAKD;PPCDC;ACAT1 | 128.93 | 0.00016 |
| purine nucleoside bisphosphate biosynthetic process (GO:0034033) | Cellular Process | Disease | PANK4;PANK2;DCAKD;PPCDC;ACAT1 | 128.93 | 0.00016 |
| ribonucleoside bisphosphate biosynthetic process (GO:0034030) | Cellular Process | Disease | PANK4;PANK2;DCAKD;PPCDC;ACAT1 | 128.93 | 0.00016 |
| actin filament capping (GO:0051693) | Cellular Process | Disease | SVIL;CAPZB;CAPZA2;CAPG;ADD3;ADD1;ADD2 | 105.48 | 0.00004 |
| barbed-end actin filament capping (GO:0051016) | Cellular Process | Disease | SVIL;CAPZB;CAPZA2;CAPG;ADD3;ADD1;ADD2 | 105.48 | 0.00004 |
| aminophospholipid transport (GO:0015917) | Cellular Process | Disease | ATP8A1;TMEM30B;ATP11B | 103.99 | 0.00275 |
| positive regulation of endothelial cell development (GO:1901552) | Cellular Process | Disease | S1PR2;F11R;ADD1 | 103.99 | 0.00275 |
| positive regulation of establishment of endothelial barrier (GO:1903142) | Cellular Process | Disease | S1PR2;F11R;ADD1 | 103.99 | 0.00275 |
| regulation of extracellular exosome assembly (GO:1903551) | Cellular Process | Disease | PDCD6IP;SDC1;STAM | 103.99 | 0.00275 |
| mRNA splice site selection (GO:0006376) | Cellular Process | Disease | SF3A1;CELF1;SFSWAP;YTHDC1;CELF2;CELF4;  LUC7L;LUC7L2;SF1 | 82.66 | 0.00002 |
| coenzyme A metabolic process (GO:0015936) | Cellular Process | Disease | PANK4;PANK2;DCAKD;PPCDC;ACAT1 | 72.88 | 0.00059 |
| dense core granule cytoskeletal transport (GO:0099519) | Cellular Process | Disease | KIF5B;KIF1C;TANC2 | 71.13 | 0.00462 |
| positive regulation of membrane depolarization (GO:1904181) | Cellular Process | Disease | MLLT11;KDR;TRPM4 | 71.13 | 0.00462 |
| posttranslational protein targeting to membrane, translocation (GO:0031204) | Cellular Process | Disease | SEC61A2;SEC61A1;SEC61G | 71.13 | 0.00462 |
| protein catabolic process in the vacuole (GO:0007039) | Cellular Process | Disease | CLN3;VPS13A;TCIRG1 | 71.13 | 0.00462 |
| peptidyl-serine modification (GO:0018209) | Cellular Process | Disease | BRSK1;DYRK3;MAST4;LRRK2;NDNF;RPS6KA4;IKBKB;RPS6KA5;TBK1;MKNK1;AKT3;RPS6KA1;STK38;MARK3;MARK2;PRKCG;CHUK;NAA10;PRKCB;PRKCE;CSNK2A2;CDC7;MAPK12;CDK2;PRKCQ;ULK1;ATM;SGK3;SGK1 | 64.75 | 0.00000 |
| peptidyl-serine phosphorylation (GO:0018105) | Cellular Process | Disease | BRSK1;DYRK3;MAST4;LRRK2;RPS6KA4;IKBKB;RPS6KA5;TBK1;MKNK1;AKT3;RPS6KA1;STK38;MARK3;MARK2;PRKCG;CHUK;PRKCB;PRKCE;CSNK2A2;CDC7;MAPK12;CDK2;PRKCQ;ULK1;ATM;SGK3;SGK1 | 61.91 | 0.00000 |
| regulation of myeloid leukocyte differentiation (GO:0002761) | Metabolic Process | Health | UBASH3B;MTOR;FOXP1 | 556.10 | 0.00006 |
| regulation of cardioblast differentiation (GO:0051890) | Metabolic Process | Health | TGFB2;TBX5 | 433.52 | 0.00107 |
| Toll signaling pathway (GO:0008063) | Metabolic Process | Health | PELI3;PELI2 | 433.52 | 0.00107 |
| negative regulation of autophagosome maturation (GO:1901097) | Metabolic Process | Health | PHF23;CLEC16A | 433.52 | 0.00107 |
| positive regulation of cardioblast differentiation (GO:0051891) | Metabolic Process | Health | TGFB2;TBX5 | 433.52 | 0.00107 |
| regulation of organ growth (GO:0046620) | Metabolic Process | Health | LATS2;WWC1;SOD1 | 366.59 | 0.00013 |
| forelimb morphogenesis (GO:0035136) | Metabolic Process | Health | TFAP2B;TBX5;TBX3 | 366.59 | 0.00013 |
| regulation of t-circle formation (GO:1904429) | Metabolic Process | Health | SLX4;ERCC1 | 306.17 | 0.00160 |
| protein geranylgeranylation (GO:0018344) | Metabolic Process | Health | PGGT1B;RABGGTA | 306.17 | 0.00160 |
| cardiac pacemaker cell development (GO:0060926) | Metabolic Process | Health | TBX5;TBX3 | 232.39 | 0.00223 |
| cardiolipin biosynthetic process (GO:0032049) | Metabolic Process | Health | TAMM41;CRLS1 | 232.39 | 0.00223 |
| histone H3-K14 acetylation (GO:0044154) | Metabolic Process | Health | ING5;BRPF3 | 232.39 | 0.00223 |
| negative regulation of DNA repair (GO:0045738) | Metabolic Process | Health | UBR5;TRIP12 | 232.39 | 0.00223 |
| negative regulation of protein K63-linked ubiquitination (GO:1900045) | Metabolic Process | Health | UBR5;TRIP12 | 232.39 | 0.00223 |
| negative regulation of protein kinase activity by regulation of protein phosphorylation (GO:0044387) | Metabolic Process | Health | NPM1;ADARB1 | 232.39 | 0.00223 |
| limb morphogenesis (GO:0035108) | Metabolic Process | Health | TFAP2B;TGFB2;TBX5;TBX3 | 202.73 | 0.00007 |
| regulation of protein ADP-ribosylation (GO:0010835) | Metabolic Process | Health | KAT2B;TINF2 | 184.74 | 0.00295 |
| TORC1 signaling (GO:0038202) | Metabolic Process | Health | CLEC16A;MTOR | 184.74 | 0.00295 |
| negative regulation of mRNA polyadenylation (GO:1900364) | Metabolic Process | Health | RNF20;NELFE | 184.74 | 0.00295 |
| anoikis (GO:0043276) | Metabolic Process | Health | IKBKG;MTOR | 151.70 | 0.00376 |
| regulation of complement-dependent cytotoxicity (GO:1903659) | Metabolic Process | Health | TGFB2;CR1L | 151.70 | 0.00376 |
| embryonic forelimb morphogenesis (GO:0035115) | Metabolic Process | Health | TBX5;TBX3 | 151.70 | 0.00376 |
| negative regulation of mRNA 3'-end processing (GO:0031441) | Metabolic Process | Health | RNF20;NELFE | 151.70 | 0.00376 |
| negative regulation of protein-containing complex disassembly (GO:0043242) | Metabolic Process | Health | PHF23;CLEC16A | 151.70 | 0.00376 |
| phosphatidylglycerol biosynthetic process (GO:0006655) | Metabolic Process | Health | TAMM41;CRLS1 | 151.70 | 0.00376 |
| SRP-dependent cotranslational protein targeting to membrane, translocation (GO:0006616) | Metabolic Process | Disease | SEC61A2;SEC61A1;ZFAND2B | 340.85 | 0.00037 |
| coenzyme A biosynthetic process (GO:0015937) | Metabolic Process | Disease | PANK4;PANK2;DCAKD;PPCDC;ACAT1 | 263.55 | 0.00002 |
| purine nucleoside bisphosphate biosynthetic process (GO:0034033) | Metabolic Process | Disease | PANK4;PANK2;DCAKD;PPCDC;ACAT1 | 263.55 | 0.00002 |
| ribonucleoside bisphosphate biosynthetic process (GO:0034030) | Metabolic Process | Disease | PANK4;PANK2;DCAKD;PPCDC;ACAT1 | 263.55 | 0.00002 |
| mRNA splice site selection (GO:0006376) | Metabolic Process | Disease | SF3A1;CELF1;SFSWAP;YTHDC1;CELF2;CELF4;  LUC7L;LUC7L2;SF1 | 182.32 | 0.00000 |
| regulation of RNA splicing (GO:0043484) | Metabolic Process | Disease | KHDRBS3;CELF1;YTHDC1;RRP1B;HNRNPLL;  PQBP1;PTBP3;CLK2;CLK1;PTBP1;HNRNPK;  ESRP2;CWC22;SRSF3;HNRNPH3;GRSF1;SF1 | 180.78 | 0.00000 |
| coenzyme A metabolic process (GO:0015936) | Metabolic Process | Disease | PANK4;PANK2;DCAKD;PPCDC;ACAT1 | 153.58 | 0.00007 |
| protein polyubiquitination (GO:0000209) | Metabolic Process | Disease | RNF31;PSMD13;CCNF;RNF8;TNFAIP3;RNF6;  BRCA1;FBXO44;PSMD8;PSMD6;RNF114;  FBXO4;TRIM27;ARIH1;FBXO9;RNF20;UBE2H;AREL1;PPIL2;SIAH2;AKTIP;HUWE1;MIB2;  UBE4B;BFAR;TRAF1;RNF144A;RNF168;  RNF167;UBE2W;PSMC6;UBE2S;TRAF6;  PSME1;UBE2V2;PSME2;ERCC8;NHLRC1;  ANAPC5;FBXL5;BIRC2 | 134.11 | 0.00000 |
| UDP-N-acetylglucosamine biosynthetic process (GO:0006048) | Metabolic Process | Disease | GNPDA2;PGM3;GNPNAT1;RENBP | 130.57 | 0.00035 |
| protein ubiquitination (GO:0016567) | Metabolic Process | Disease | CCNF;BRCA1;PSMD8;PSMD6;RNF114;FBXO4;TRIM27;ARIH1;FBXO9;RNF43;AREL1;USP5;  AKTIP;UBE4B;TRPC4AP;RNF168;RNF144A;  RNF167;TRAF6;PSME1;UBE2V2;PSME2;  NHLRC1;PDZRN3;BIRC2;RNF31;CUL5;UHRF2;PSMD13;CTR9;RNF8;DTX1;WDR61;RNF6;  HIF1A;FBXO44;PCNP;RNF20;TRIM40;UBE2H;PPIL2;CRBN;FANCL;SIAH2;HUWE1;MIB2;  BFAR;PEX12;CNOT4;UBE2W;PSMC6;UBE2S;  RNF181;ERCC8;UBA1;FBXL5;RNF185 | 120.92 | 0.00000 |
| ubiquitin-dependent protein catabolic process (GO:0006511) | Metabolic Process | Disease | KLHL15;PSMD13;ZFAND2B;RNF8;TNFAIP3;  RNF6;PSMD8;PCNP;ATXN3;PSMD6;RNF114;  ARIH1;UBL7;RNF20;RNF43;UBE2H;AREL1;  CRBN;SIAH2;HUWE1;UBE4B;BFAR;NTAN1;  TRPC4AP;RNF144A;RNF168;CNOT4;RNF167;COPS3;UBE2W;PSMC6;UBE2S;TOLLIP;PSME1;PSME2;SPOPL;ERCC8;NHLRC1;ANAPC5;  UBA1;RNF185;BIRC2 | 109.79 | 0.00000 |
| peptidyl-serine modification (GO:0018209) | Metabolic Process | Disease | BRSK1;DYRK3;MAST4;LRRK2;NDNF;RPS6KA4;IKBKB;RPS6KA5;TBK1;MKNK1;AKT3;RPS6KA1;STK38;PRKCG;CHUK;NAA10;PRKCB;PRKCE;  CDC7;CDK2;PRKCQ;ULK1;ATM;SGK3;SGK1 | 109.07 | 0.00000 |
| amino sugar biosynthetic process (GO:0046349) | Metabolic Process | Disease | GNPDA2;PGM3;GNPNAT1;RENBP | 108.80 | 0.00052 |
| regulation of pattern recognition receptor signaling pathway (GO:0062207) | Metabolic Process | Disease | XIAP;CD36;BIRC2 | 107.96 | 0.00190 |
| regulation of protein K63-linked ubiquitination (GO:1900044) | Metabolic Process | Disease | GPS2;PTPN22;BIRC2 | 107.96 | 0.00190 |
| peptidyl-serine phosphorylation (GO:0018105) | Metabolic Process | Disease | PRKCG;BRSK1;DYRK3;MAST4;CHUK;PRKCB;  PRKCE;LRRK2;CDC7;RPS6KA4;IKBKB;RPS6KA5;TBK1;MKNK1;AKT3;RPS6KA1;STK38;CDK2;  PRKCQ;ULK1;ATM;SGK3;SGK1 | 99.96 | 0.00000 |
| phosphorylation (GO:0016310) | Metabolic Process | Disease | BRSK1;RNASEL;BRSK2;PANK2;DYRK3;DGKB;  LRRK2;PIK3CD;STK3;HK1;IKBKB;RPS6KA4;  PPP4R1;TBK1;RPS6KA5;MKNK1;AKT3;STK38;PAK6;JAK3;PRKCG;ACVR1;BRD2;GK;CHUK;  PRKCB;RYK;PRKCE;DGKZ;CDK11B;CLK2;  TOLLIP;WNK2;CDK2;COQ8B;ULK1;ATM;SGK3;ERG;TNIK;SIK2;SGK1;CDK14;FGFR1 | 95.82 | 0.00000 |
| purine ribonucleotide biosynthetic process (GO:0009152) | Metabolic Process | Disease | PANK4;PANK2;IMPDH1;DCAKD;AMPD2;  AMPD3;ADCY7;PAPSS2;PPCDC;ACAT1 | 95.26 | 0.00000 |
| modification-dependent protein catabolic process (GO:0019941) | Metabolic Process | Disease | KLHL15;PSMD13;RNF8;RNF6;ATXN3;RNF114;ARIH1;UBL7;RNF20;RNF43;UBE2H;AREL1;  SIAH2;UBE4B;BFAR;NTAN1;TRPC4AP;  RNF144A;RNF168;CNOT4;RNF167;COPS3;  UBE2S;TOLLIP;ANAPC5;UBA1;RNF185 | 94.79 | 0.00000 |
| AMP metabolic process (GO:0046033) | Metabolic Process | Disease | AK4;AMPD2;AMPD3;XDH | 92.36 | 0.00072 |
| UDP-N-acetylglucosamine metabolic process (GO:0006047) | Metabolic Process | Disease | GNPDA2;PGM3;GNPNAT1;RENBP | 92.36 | 0.00072 |
| regulation of cellular response to growth factor stimulus (GO:0090287) | Biological Regulation | Health | TFAP2B;FST;CASK;SLIT2;FSTL1 | 679.80 | 0.00000 |
| regulation of myeloid leukocyte differentiation (GO:0002761) | Biological Regulation | Health | UBASH3B;MTOR;FOXP1 | 657.94 | 0.00004 |
| TORC1 signaling (GO:0038202) | Biological Regulation | Health | CLEC16A;MLST8;MTOR | 657.94 | 0.00004 |
| regulation of cardioblast differentiation (GO:0051890) | Biological Regulation | Health | TGFB2;TBX5 | 511.53 | 0.00084 |
| cardiac muscle cell proliferation (GO:0060038) | Biological Regulation | Health | NDRG4;TGFB2 | 511.53 | 0.00084 |
| striated muscle cell proliferation (GO:0014855) | Biological Regulation | Health | NDRG4;TGFB2 | 511.53 | 0.00084 |
| negative regulation of autophagosome maturation (GO:1901097) | Biological Regulation | Health | PHF23;CLEC16A | 511.53 | 0.00084 |
| positive regulation of cardioblast differentiation (GO:0051891) | Biological Regulation | Health | TGFB2;TBX5 | 511.53 | 0.00084 |
| forelimb morphogenesis (GO:0035136) | Biological Regulation | Health | TFAP2B;TBX5;TBX3 | 435.00 | 0.00009 |
| negative regulation of chemokine-mediated signaling pathway (GO:0070100) | Biological Regulation | Health | SLIT2;SH2B3 | 362.01 | 0.00125 |
| negative regulation of lamellipodium organization (GO:1902744) | Biological Regulation | Health | PLXNB3;SLIT2 | 362.01 | 0.00125 |
| cardiac pacemaker cell development (GO:0060926) | Biological Regulation | Health | TBX5;TBX3 | 275.29 | 0.00173 |
| histone H3-K14 acetylation (GO:0044154) | Biological Regulation | Health | ING5;BRPF3 | 275.29 | 0.00173 |
| limb morphogenesis (GO:0035108) | Biological Regulation | Health | TFAP2B;TGFB2;TBX5;TBX3 | 243.00 | 0.00005 |
| regulation of chemokine-mediated signaling pathway (GO:0070099) | Biological Regulation | Health | SLIT2;SH2B3 | 219.24 | 0.00230 |
| regulation of protein ADP-ribosylation (GO:0010835) | Biological Regulation | Health | KAT2B;TINF2 | 219.24 | 0.00230 |
| plasma membrane phospholipid scrambling (GO:0017121) | Biological Regulation | Health | ANO6;ANO7 | 219.24 | 0.00230 |
| hippo signaling (GO:0035329) | Biological Regulation | Health | TEAD4;LATS2;WWC1;DVL2 | 185.14 | 0.00009 |
| negative regulation of macroautophagy (GO:0016242) | Biological Regulation | Health | PHF23;CLEC16A;NUPR1;MTOR | 185.14 | 0.00009 |
| anoikis (GO:0043276) | Biological Regulation | Health | IKBKG;MTOR | 180.32 | 0.00294 |
| bone cell development (GO:0098751) | Biological Regulation | Health | SH2B3;FOXP1 | 180.32 | 0.00294 |
| regulation of complement-dependent cytotoxicity (GO:1903659) | Biological Regulation | Health | TGFB2;CR1L | 180.32 | 0.00294 |
| embryonic forelimb morphogenesis (GO:0035115) | Biological Regulation | Health | TBX5;TBX3 | 180.32 | 0.00294 |
| negative regulation of protein-containing complex disassembly (GO:0043242) | Biological Regulation | Health | PHF23;CLEC16A | 180.32 | 0.00294 |
| actin filament capping (GO:0051693) | Biological Regulation | Disease | SVIL;CAPZB;CAPZA2;CAPG;ADD3;ADD1;ADD2 | 278.80 | 0.00000 |
| barbed-end actin filament capping (GO:0051016) | Biological Regulation | Disease | SVIL;CAPZB;CAPZA2;CAPG;ADD3;ADD1;ADD2 | 278.80 | 0.00000 |
| aminophospholipid transport (GO:0015917) | Biological Regulation | Disease | ATP8A1;TMEM30B;ATP11B | 255.86 | 0.00047 |
| positive regulation of endothelial cell development (GO:1901552) | Biological Regulation | Disease | S1PR2;F11R;ADD1 | 255.86 | 0.00047 |
| positive regulation of establishment of endothelial barrier (GO:1903142) | Biological Regulation | Disease | S1PR2;F11R;ADD1 | 255.86 | 0.00047 |
| peptidyl-serine phosphorylation (GO:0018105) | Biological Regulation | Disease | BRSK1;DYRK3;MAST4;LRRK2;RPS6KA4;IKBKB;RPS6KA5;MKNK1;AKT3;RPS6KA1;STK38;  MARK3;MARK2;PRKCG;CHUK;PRKCB;PRKCE;CSNK2A2;CDC7;MAPK12;CDK2;PRKCQ;ULK1;ATM;SGK3;SGK1 | 193.90 | 0.00000 |
| protein phosphorylation (GO:0006468) | Biological Regulation | Disease | DYRK3;MAST4;LRRK2;PIK3CD;IKBKB;RPS6KA4;PPP3CB;RPS6KA5;AKT3;RPS6KA1;KDR;JAK3;EPHB4;PRKCG;ACVR1;BRD2;CHUK;PRKCB;  PRKCE;IGFBP3;CSNK2A2;DAPK3;TYRO3;  PRKCQ;ULK1;SGK3;ERG;TNIK;SIK2;SGK1;  BRSK1;CSF1R;BRSK2;STK3;PRDX4;MKNK1;  STK38;PAK6;CSK;MARK3;MARK2;RYK;CDC7;  CDK11B;MAPK12;CLK2;MOS;TBCK;FES;WNK2;CDK2;ATM;CDK14;FGFR3;FGFR1 | 172.53 | 0.00000 |
| peptidyl-serine modification (GO:0018209) | Biological Regulation | Disease | BRSK1;DYRK3;MAST4;LRRK2;RPS6KA4;IKBKB;RPS6KA5;MKNK1;AKT3;RPS6KA1;STK38;  MARK3;MARK2;PRKCG;CHUK;PRKCB;PRKCE;CSNK2A2;CDC7;MAPK12;CDK2;PRKCQ;ULK1;ATM;SGK3;SGK1 | 164.21 | 0.00000 |
| regulation of axonogenesis (GO:0050770) | Biological Regulation | Disease | TIAM2;TIAM1;BRSK1;BRSK2;SIPA1L1;PLXND1;SLITRK1;CHN1;PLXNB1;SLIT2;MARK2 | 143.13 | 0.00000 |
| regulation of transcription by RNA polymerase II (GO:0006357) | Biological Regulation | Disease | ATF2;PRDM4;MYT1L;RAX;ZBTB20;JMJD1C;  YEATS4;RORA;IKZF1;IKZF2;IKZF3;ETS1;SPIB;  RPS6KA4;RPS6KA5;RPS6KA1;EPC1;TRIM27;  PITX2;CCNL1;SOX6;PITX1;ACVR1;SMARCC2;  PRKCB;SOX13;EBF1;ARID5A;RFX3;HNF1A;  HCFC1;ZSCAN20;RFX5;ZSCAN25;ERG;HOMEZ;CSF1R;DLX1;ENY2;CTR9;CREM;HIF1A;  MED12L;MTDH;GLIS2;MIER3;MIER2;ALX1;  SKIL;BPTF;TFAP2A;DR1;TFAP2D;PTCH1;PBX3;TRERF1;ZFHX4;NR2F6;NFKB2;MED13L;  DRAP1;ZBTB9;DLG1;HNRNPK;TADA2A;  MRGBP;CNOT2;SP3;PHF14;ATM;TCF4;  ZSCAN2;LHX8;BARHL1;GMEB1;CIITA;BRCA1;HOXC10;PHF8;IKBKB;PPP3CB;MED13;  MECOM;HEY1;SIN3B;MYBL2;TGIF1;BRD2;  CHUK;NCOA5;TCF12;ARID1A;ZFX;ETV5;PAX1;ETV6;PHF20L1;NCOR2;CREB3;MED25;NCOR1;ELF2;IRF4;ELF4;TRAF6;IRF2;RARB;ARHGEF2;LCORL;VGLL1;JDP2;SMARCD2;KDM3A;  ZBTB49;DBX2;GPS2;ZBTB45;BARX1;DLL1;  RELA;ARNTL2;RXRB;MAPK7;BRD9;E2F6;E2F7;SPDEF;TBX1;SPEN;TCF7L1;SKOR2;NFYA;  STAT2;GTF2H2;MEIS3;GRHL2;TBX19;USF1;  BATF;PER1;GFI1B;MEIS1;TNIP2;BAX;PAXBP1;LPIN2;TNRC6B | 141.69 | 0.00000 |
| regulation of transcription, DNA-templated (GO:0006355) | Biological Regulation | Disease | CD86;ATF2;MYT1L;RAX;ZBTB20;JMJD1C;  YEATS4;RORA;IKZF1;IKZF2;IKZF3;ETS1;CCAR1;SPIB;RPS6KA4;RPS6KA5;GPBP1;RPS6KA1;  EPC1;PITX2;CCNL1;SOX6;PITX1;ACVR1;  RBFOX2;SMARCC2;PRKCB;DAPK3;SOX13;  EBF1;ARID5A;RFX3;HNF1A;HCFC1;ZSCAN20;  RFX5;PRKCQ;ZSCAN25;ERG;HOMEZ;DLX1;  ENY2;CTR9;CREM;HIF1A;MED12L;MTDH;  GLIS2;SBNO1;ALX1;MAP2K7;BPTF;TFAP2A;  TFAP2D;PBX3;CDC6;TRERF1;ZFHX4;NR2F6;  NFKB2;MED13L;MLLT11;DRAP1;ZBTB9;  HNRNPK;TADA2A;CNOT7;MRGBP;CNOT2;  SP3;PHF14;TCF4;CDK14;BAP1;ZSCAN2;LHX8;BARHL1;GMEB1;CIITA;BRCA1;HOXC10;PHF8;  IKBKB;PPP3CB;MED13;MECOM;HEY1;UIMC1;HMG20A;MYBL2;TGIF1;BRD2;CHUK;TCF12;  CBFA2T2;ARID1A;ZFX;CBFA2T3;ETV5;PAX1;  ETV6;PHF20L1;NCOR2;CREB3;NCOR1;ELF2;  IRF4;ELF4;TRAF6;IRF2;LCORL;VGLL1;PFDN5;  JDP2;SMARCD2;KDM3A;MYCBP;ZBTB49;  SRRT;DBX2;ZBTB45;RNF6;BARX1;RELA;  ARNTL2;RXRB;PAK6;BRD9;E2F6;E2F7;SPDEF;TBX1;SPEN;TCF7L1;SKOR2;NFYA;STAT2;  GTF2H2;MEIS3;GRHL2;TBX19;CDK11B;USF1;BATF;PER1;GFI1B;MEIS1 | 134.22 | 0.00000 |
| phosphorylation (GO:0016310) | Biological Regulation | Disease | BRSK1;BRSK2;DYRK3;DGKB;LRRK2;PIK3CD;  STK3;HK1;IKBKB;RPS6KA4;PPP3CB;RPS6KA5;MKNK1;AKT3;STK38;PAK6;CSK;JAK3;MARK3;MARK2;PRKCG;ACVR1;BRD2;CHUK;PRKCB;  RYK;PRKCE;IGFBP3;DAPK3;DGKZ;CDK11B;  CLK2;TBCK;WNK2;CDK2;ULK1;ATM;SGK3;  ERG;TNIK;SIK2;SGK1;CDK14;FGFR1 | 134.04 | 0.00000 |
| regulation of chemokine-mediated signaling pathway (GO:0070099) | Biological Regulation | Disease | SLIT2;HIF1A;SH2B3 | 133.78 | 0.00125 |
| regulation of Golgi to plasma membrane protein transport (GO:0042996) | Biological Regulation | Disease | CLN3;CSK;ATP2C1 | 133.78 | 0.00125 |
| regulation of pattern recognition receptor signaling pathway (GO:0062207) | Biological Regulation | Disease | XIAP;CD36;BIRC2 | 133.78 | 0.00125 |
| positive regulation of GTPase activity (GO:0043547) | Biological Regulation | Disease | BCAR3;ITGB1;TBC1D9B;DOCK9;F11R;  RASGRP2;DOCK10;SIPA1L1;TBC1D30;  TBC1D10C;CCL25;CCL22;ARHGEF16;  ARHGEF19;TSC1;ARHGAP27;GMIP;TIAM2;  TIAM1;TBCK;TBC1D4;SGSM3;SGSM2;  RAPGEF1;PLXNB1;MAPRE2;RAPGEF3;  RAB3GAP1 | 125.26 | 0.00000 |
| histone H3-K27 demethylation (GO:0071557) | Biological Regulation | Disease | KDM6A;PHF8 | 107.03 | 0.00805 |
| kidney morphogenesis (GO:0060993) | Biological Regulation | Disease | LRRK2;WNT7B | 107.03 | 0.00805 |
| lipid transport across blood-brain barrier (GO:1990379) | Biological Regulation | Disease | SLC27A1;CD36 | 107.03 | 0.00805 |
| negative regulation of autophagosome maturation (GO:1901097) | Biological Regulation | Disease | RUBCN;PHF23 | 107.03 | 0.00805 |
| positive regulation of Golgi to plasma membrane protein transport (GO:0042998) | Biological Regulation | Disease | CLN3;ATP2C1 | 107.03 | 0.00805 |
| positive regulation of macrophage proliferation (GO:0120041) | Biological Regulation | Disease | CSF1R;IL34 | 107.03 | 0.00805 |
| positive regulation of mitochondrial depolarization (GO:0051901) | Biological Regulation | Disease | MLLT11;KDR | 107.03 | 0.00805 |
| regulation of nucleotide-binding oligomerization domain containing signaling pathway (GO:0070424) | Biological Regulation | Disease | XIAP;BIRC2 | 107.03 | 0.00805 |
| regulation of voltage-gated sodium channel activity (GO:1905150) | Biological Regulation | Disease | FGF14;FGF11 | 107.03 | 0.00805 |
| toll-like receptor 2 signaling pathway (GO:0034134) | Biological Regulation | Disease | TNIP2;LGALS9 | 107.03 | 0.00805 |

**Appendix Table 7** Enriched gene ontologies (Molecular Function) in the mouse host oral metatranscriptome with a Enrichr-calculated combined score greater than 12, in health (blue) and disease (red)

| **Enriched GO Molecular Function** | **Enriched Condition** | **P-value** | **Combined Score** | **Genes** |
| --- | --- | --- | --- | --- |
| RNA polymerase I core promoter sequence-specific DNA binding (GO:0001164) | Health | 0.0027 | 89.543 | TAF1B;TAF1C;UBTF |
| RNA polymerase I transcription regulatory region sequence-specific DNA binding (GO:0001163) | Health | 0.0027 | 89.543 | TAF1B;TAF1C;UBTF |
| protein geranylgeranyltransferase activity (GO:0004661) | Health | 0.0135 | 72.306 | PGGT1B;RABGGTA |
| core promoter sequence-specific DNA binding (GO:0001046) | Health | 0.0004 | 45.691 | KLF10;NPM1;TAF1B;TAF1C;UBTF;FOXP1;TAF1 |
| voltage-gated channel activity (GO:0022832) | Health | 0.0270 | 36.413 | CALHM1;ANO6 |
| aldehyde dehydrogenase (NAD+) activity (GO:0004029) | Health | 0.0120 | 33.500 | ALDH2;ALDH7A1;ALDH9A1 |
| phosphatidylinositol-3,4,5-trisphosphate binding (GO:0005547) | Health | 0.0019 | 32.724 | MYO1B;ZFYVE16;PLEKHB2;ANXA8;ZFYVE1;  FERMT2 |
| translation elongation factor activity (GO:0003746) | Health | 0.0148 | 28.989 | EEF1B2;GTPBP2;GTPBP1 |
| 1-phosphatidylinositol binding (GO:0005545) | Health | 0.0180 | 25.343 | ZFYVE16;WDFY3;ZFYVE1 |
| aldehyde dehydrogenase [NAD(P)+] activity (GO:0004030) | Health | 0.0180 | 25.343 | ALDH2;ALDH7A1;ALDH9A1 |
| cytidylyltransferase activity (GO:0070567) | Health | 0.0440 | 22.490 | PCYT1A;TAMM41 |
| glucosyltransferase activity (GO:0046527) | Health | 0.0440 | 22.490 | POGLUT1;GBA2 |
| ATPase activity, coupled to transmembrane movement of ions, rotational mechanism (GO:0044769) | Health | 0.0215 | 22.349 | TCIRG1;ATP6V0D1;ATP6V1C1 |
| proton-transporting ATPase activity, rotational mechanism (GO:0046961) | Health | 0.0215 | 22.349 | TCIRG1;ATP6V0D1;ATP6V1C1 |
| intracellular calcium activated chloride channel activity (GO:0005229) | Health | 0.0254 | 19.854 | TTYH3;ANO6;ANO7 |
| intracellular chloride channel activity (GO:0061778) | Health | 0.0254 | 19.854 | TTYH3;ANO6;ANO7 |
| ion gated channel activity (GO:0022839) | Health | 0.0162 | 18.913 | CALHM1;TTYH3;ANO6;ANO7 |
| Tat protein binding (GO:0030957) | Health | 0.0536 | 18.430 | NPM1;CTDP1 |
| UDP-glucosyltransferase activity (GO:0035251) | Health | 0.0536 | 18.430 | POGLUT1;UGGT1 |
| enoyl-CoA hydratase activity (GO:0004300) | Health | 0.0536 | 18.430 | AUH;ECHDC2 |
| ubiquitin-like protein conjugating enzyme activity (GO:0061650) | Health | 0.0143 | 16.270 | UBE2F;AKTIP;UBE2D3;UBE2G1;TAF1 |
| intracellular ligand-gated ion channel activity (GO:0005217) | Health | 0.0639 | 15.399 | RYR2;RASA3 |
| nuclear export signal receptor activity (GO:0005049) | Health | 0.0639 | 15.399 | CSE1L;XPO6 |
| platelet-derived growth factor binding (GO:0048407) | Health | 0.0639 | 15.399 | COL1A1;COL3A1 |
| purine ribonucleotide transmembrane transporter activity (GO:0005346) | Health | 0.0639 | 15.399 | SLC35B2;SLC19A1 |
| protein serine/threonine kinase activity (GO:0004674) | Health | 0.0008 | 14.936 | TRIO;MAST2;ILK;STK19;MYLK;TBK1;IRAK2;  STK36;TLK1;RIOK1;CAMK2G;MAP4K4;CDKL3;  RIOK3;CASK;CSNK1E;HIPK1;MTOR;CLK1;LATS2;  RPS6KB2;AAK1;SIK2;RAF1;CDK16;TAF1 |
| antiporter activity (GO:0015297) | Health | 0.0391 | 14.424 | SLC35C2;SLC35E4;SLC35D2 |
| transcription coactivator binding (GO:0001223) | Health | 0.0391 | 14.424 | MED25;ZBTB49;PPARD |
| microtubule plus-end binding (GO:0051010) | Health | 0.0391 | 14.424 | CLIP1;DST;FBXW11 |
| lysophospholipase activity (GO:0004622) | Health | 0.0444 | 13.091 | ABHD16A;PNPLA6;PLB1 |
| motor activity (GO:0003774) | Health | 0.0128 | 13.073 | DNAH17;KIF13A;MYO6;KIF1C;KIF24;KIF1B;KIF27 |
| RNA stem-loop binding (GO:0035613) | Health | 0.0748 | 13.065 | RC3H1;MYH10 |
| adenine nucleotide transmembrane transporter activity (GO:0000295) | Health | 0.0748 | 13.065 | SLC35B2;SLC19A1 |
| transmembrane receptor protein tyrosine kinase adaptor activity (GO:0005068) | Health | 0.0748 | 13.065 | SHC1;SH2B3 |
| neuroligin family protein binding (GO:0097109) | Disease | 0.0008 | 220.175 | DLG4;NRXN1;NRXN3;NRXN2 |
| RNA polymerase II general transcription initiation factor binding (GO:0001091) | Disease | 0.0127 | 50.466 | DRAP1;TCF4;ESR1 |
| Lys63-specific deubiquitinase activity (GO:0061578) | Disease | 0.0207 | 19.924 | ATXN3;USP27X;TNFAIP3;STAMBP |
| transcription corepressor binding (GO:0001222) | Disease | 0.0040 | 19.133 | SUZ12;PER1;CTBP2;CTBP1;NEK6;CNOT2;RORA;  ESR1;EZH2 |
| aminophospholipid flippase activity (GO:0015247) | Disease | 0.0372 | 19.016 | ATP8A1;TMEM30B;ATP11A |
| ferroxidase activity (GO:0004322) | Disease | 0.0372 | 19.016 | HEPH;FTH1;CYB561D2 |
| oxidoreductase activity, acting on metal ions, oxygen as acceptor (GO:0016724) | Disease | 0.0372 | 19.016 | HEPH;FTH1;CYB561D2 |
| phosphatidylinositol phosphate 4-phosphatase activity (GO:0034596) | Disease | 0.0372 | 19.016 | INPP4A;INPP4B;INPP5F |
| ribosomal protein S6 kinase activity (GO:0004711) | Disease | 0.0372 | 19.016 | RPS6KA4;RPS6KA3;RPS6KA1 |
| mannosyltransferase activity (GO:0000030) | Disease | 0.0094 | 16.792 | ALG8;DPY19L1;POMT1;ALG2;TMTC3;PIGZ;ALG1 |
| phosphotransferase activity, for other substituted phosphate groups (GO:0016780) | Disease | 0.0296 | 15.499 | SAMD8;PIGO;SGMS1;PIGN |
| carboxy-lyase activity (GO:0016831) | Disease | 0.0122 | 14.863 | UROD;GAD1;UXS1;AZIN2;CSAD;AZIN1;PPCDC |
| DNA replication origin binding (GO:0003688) | Disease | 0.0122 | 14.863 | ORC4;MCM7;DHX9;MCM9;ORC2;KAT7;CDC6 |
| guanyl-nucleotide exchange factor activity (GO:0005085) | Disease | 0.0007 | 14.686 | DOCK4;DOCK9;ARHGEF28;ITSN1;MYCBP2;  RASGRP2;RCBTB2;DOCK10;CYTH3;PREX1;RINL;  DENND5A;RALGPS1;ARFGEF1;ARHGEF16;HPS4;  RAB3IL1;VAV1;TIAM1;CCDC88A;FRMD7;ELMO1;RAPGEF1;PREB;ARHGEF2;ARHGEF7;DOCK2;  LAMTOR3;MON1A;RAPGEF3;RAB3GAP1 |
| GTPase activator activity (GO:0005096) | Disease | 0.0003 | 13.464 | DOCK4;ARHGAP8;DOCK9;LRRK2;ITSN1;RCBTB2;DOCK10;PREX1;RGS2;RINL;SIPA1L1;TBC1D30;  TBC1D10C;DENND5A;RALGPS1;ARHGEF16;  RAB3IL1;GMIP;VAV1;ARFGAP1;TIAM1;FRMD7;  TBC1D20;TBC1D4;RASA4;RAPGEF1;ELMO1;  PREB;ARHGEF2;ARHGEF7;DOCK2;LLGL1;  RAPGEF3;SEC23A;TBC1D9B;ARHGEF28;MYCBP2;ARHGAP17;RASGRP2;CYTH3;ARHGAP20;  RACGAP1;CHN1;BNIP2;RGS20;SEC23B;ARFGEF1;STARD13;RANBP3;HPS4;ARHGAP27;ADAP1;  CCDC88A;TBCK;ARHGAP30;SGSM3;SGSM2;  LAMTOR3;MON1A;RAB3GAP1 |
| carbon-nitrogen ligase activity, with glutamine as amido-N-donor (GO:0016884) | Disease | 0.0545 | 13.444 | CAD;NADSYN1;PFAS |
| G protein-coupled glutamate receptor binding (GO:0035256) | Disease | 0.0545 | 13.444 | NECAB2;PRNP;HOMER1 |
| phosphatidylinositol phosphate 5-phosphatase activity (GO:0034595) | Disease | 0.0545 | 13.444 | INPP5F;PTPMT1;SYNJ2 |
| procollagen-proline dioxygenase activity (GO:0019798) | Disease | 0.0545 | 13.444 | P4HA2;P3H2;P3H3 |
| RNA polymerase II C-terminal domain binding (GO:0099122) | Disease | 0.0545 | 13.444 | PCIF1;SCAF1;SCAF4 |
| tubulin-glutamic acid ligase activity (GO:0070740) | Disease | 0.0545 | 13.444 | TTLL5;TTLL4;TTLL11 |
| transcription coregulator binding (GO:0001221) | Disease | 0.0060 | 12.844 | SUZ12;CTBP2;CTBP1;ZBTB49;NEK6;RORA;ESR1;  RELA;CIT;PER1;MED25;CNOT2;EZH2 |
| acylglycerol O-acyltransferase activity (GO:0016411) | Disease | 0.0214 | 12.698 | DGAT2;DGAT1;LPCAT2;ABHD5;AGPAT1;AGPAT4 |
| transcription coactivator binding (GO:0001223) | Disease | 0.0214 | 12.698 | MED25;ZBTB49;RORA;ESR1;RELA;CIT |
| mRNA methyltransferase activity (GO:0008174) | Disease | 0.0405 | 12.358 | METTL14;RNMT;PCIF1;CMTR1 |
| 3'-5' DNA helicase activity (GO:0043138) | Disease | 0.0295 | 12.344 | HELQ;FANCM;MCM7;DHX9;RECQL |

**Appendix Table 8** Loss of alveolar bone levels (expressed in mm) calculated by subtracting the mean CEJ-ABC distance of each mouse from the mean CEJ-ABC distance of the control mice, which was used as a zero baseline. The negative value indicates bone loss, and each value corresponds to an individual mouse in each group.

| **Health** | **Disease** |
| --- | --- |
| 0.0114087 | -0.01784855 |
| 0.00748357 | -0.03822899 |
| 0.00615507 | -0.01187029 |
| -0.00059312 | -0.03282008 |
| -0.01368188 | -0.04760399 |
| -0.01476884 | -0.04728696 |
| 0.00389058 | -0.0185279 |
|  | -0.03463168 |
